# Nine New Antibacterial Diterpenes and Steroids from the South China Sea Soft Coral *Lobophytum catalai* Tixier-Durivault

**DOI:** 10.3390/md22010050

**Published:** 2024-01-20

**Authors:** Sheng-Hui Zhu, Yuan-Min Chang, Ming-Zhi Su, Li-Gong Yao, Song-Wei Li, Hong Wang, Yue-Wei Guo

**Affiliations:** 1 School of Medicine, Shanghai University, Shanghai 200444, China; zhushenghui0115@163.com; 2 Shandong Laboratory of Yantai Drug Discovery, Bohai Rim Advanced Research Institute for Drug Discovery, Yantai 264117, China; 13153672940@163.com (Y.-M.C.); smz0310@163.com (M.-Z.S.); yaoligong@simm.ac.cn (L.-G.Y.); 3 Collaborative Innovation Center of Yangtze River Delta Region Green Pharmaceuticals and College of Pharmaceutical Science, Zhejiang University of Technology, Hangzhou 310014, China

**Keywords:** *Lobophytum catalai*, antibacterial activity, soft coral, structural elucidation

## Abstract

Five new cembrane-type diterpenes, lobocalines A–E (**1**–**5**), and four new steroids, lobocaloids A–D (**9**–**12**), along with six known related compounds (**6**–**8** and **13**–**15**) were isolated from the Yalong Bay soft coral *Lobophytum catalai* Tixier-Durivault. The structures of the new compounds were elucidated by extensive spectroscopic analysis, NMR calculation with DP4+ analysis, time-dependent density functional theory–electronic circular dichroism (TDDFT-ECD) calculations, X-ray diffraction analyses and comparison with the reported spectroscopic data of known compounds. Further, with the aid of X-ray diffraction analysis, the structure of lobocrasol B (**15**) was firmly revised as **15a**. In in vitro bioassays, compound **2** showed moderate antibacterial activities against fish pathogenic bacteria *Streptococcus parauberis* KSP28 and *Phoyobacterium damselae* FP2244 with minimum inhibitory concentration (MIC) values of 8.7 and 17.3 µg/mL, respectively. All the steroids exhibited antibacterial activities against the *S. parauberis* KSP28 with MIC values ranging from 12.3 to 53.6 µg/mL. Compounds **2**, **7** and **14** have remarkable inhibitory effects on the hemolysin production of *Staphylococcus aureus*, while compounds **8**–**12** have medium inhibitory effects on the pyocyanin production in *Pseudomonas aeruginosa*.

## 1. Introduction

Aquaculture is a crucial component of agriculture, the foundational element of agricultural economic growth and a crucial source of food. With the rapid and intensive development of aquaculture, many problems related to aquatic animal diseases have gradually been exposed. The main pathogens of aquatic animals include bacteria, viruses, parasites, etc. It is estimated that economic loss of aquatic animals caused by numerous bacteria (such as *Streptococcus parauberis* and *Aeromonas salmonicida*) accounts for 58%, which is the most serious factor leading to the economic loss of aquaculture [1,2,3]. As antibiotic resistance and drug residues in aquaculture have become global focus, the search for new and effective antibacterial compounds against fish pathogenic bacteria has become more important and urgent. In addition, bacteria *Staphylococcus aureus* and *Pseudomonas aeruginosa* could also cause a broad range of life-threatening diseases in humans. For example, *S. aureus* cause infectious disease by the production of virulence factors such as hemolysins [4]. Pyocyanin, the secondary metabolite produced by *P. aeruginosa*, is considered to play an essential role of oxidative stress in the infection [5].

Marine invertebrates are important sources of natural products and have been recognized to be the rich source of bioactive secondary metabolites with structural diversity [6,7,8,9,10]. Therefore, the marine invertebrates have enormous potential for exploring new marine drugs. In particular, soft corals of the genus *Lobophytum* (order Alcyonacea, family Alcyoniidae) have become an intensive research subject, which produced varieties of structurally intriguing and biologically interesting molecules [11,12]. More recently, two breakthrough studies have been reported toward the biosynthesis of terpenes in the soft corals and the identification of coral-encoded terpene cyclase genes enabled the enzyme that construct the coral-exclusive terpene scaffolds [13,14], which attract significant attentions to conduct the metabolic patterns of marine soft coral metabolites. In addition, a survey of the extensive literature revealed that abundant *Lobophytum* soft corals are inhabiting in the South China Sea, and the titled species *L. catalai* has been rarely studied. Until now, only a total of four studies have been reported on the chemical investigations of this species, leading to the discovery of several terpenes [15,16,17] and steroids [18] with anti-inflammatory [17] and/or cytotoxic [17] activities.

**Figure 1:**
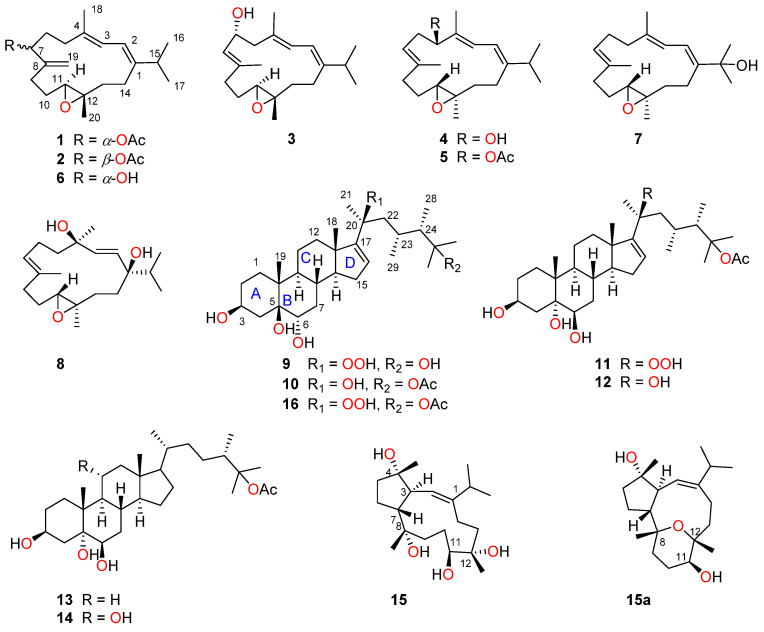
Chemical structures of compounds **1**–**16**.

In the course of our continuing effort to explore chemically fascinating and biologically active secondary metabolites of South China Sea marine invertebrates [12], we recently carried out a chemical investigation on the soft coral *L. catalai* collected off Yalong Bay, Hainan Province, China, resulting in the isolation and characterization of nine new compounds, namely lobocalines A–I (**1**–**5** and **9**–**12**), together with six known compounds (**6**–**8** and **13**–**15**) (Figure 1). The structural difference between the five new diterpenes **1**–**5** is reflected in the different positions of substituents. Moreover, the structural diversities of six steroids **9**–**14** are mainly attributed to the different configurations of C-5 and C-6 in ring B of steroidal nucleus and the variations in functional groups on the side chains. What is more is that the structural determinations of new compounds are also a challenging problem, and X-ray diffraction analysis as a powerful tool [19] was applied in this study. Herein, we report the detailed isolation, planar structural elucidation, stereochemistry determination, and antibacterial activity evaluation of these isolated compounds.

## 2. Results

Samples of *L. catalai* were frozen immediately to –20 ℃ after collection and stored at that temperature before they were exhaustively extracted by acetone. The Et_2_O-soluble portion of the acetone extract was subjected to repeated column chromatography (CC) (silica gel, Sephadex LH-20 and reversed-phase HPLC) to yield five new cembrane-type diterpenes lobocalines A–E (**1**–**5**) and four new steroids lobocaloids A–D (**9**–**12**), along with six known analogues (**6**–**8** and **13**–**15**). The known compounds were rapidly characterized as sarcoboettgerol D (**6**) [20], 11,12-epoxy-1*E*,3*E*,7*E*-cembratrien-15-ol (**7**) [21], sarcophytrol L (**8**) [22], 24*ξ*-methylcholestane-3*β*,5*α*,6*β*,25-tetrol-25-monoacetate (**13**) [23], acutumosterol B (**14**) [24], and lobophytrol B (**15**) [25], respectively, by the comparison of their NMR data and optical rotation [*α*]_D_ values with those reported in the literature.

**Figure 2:**
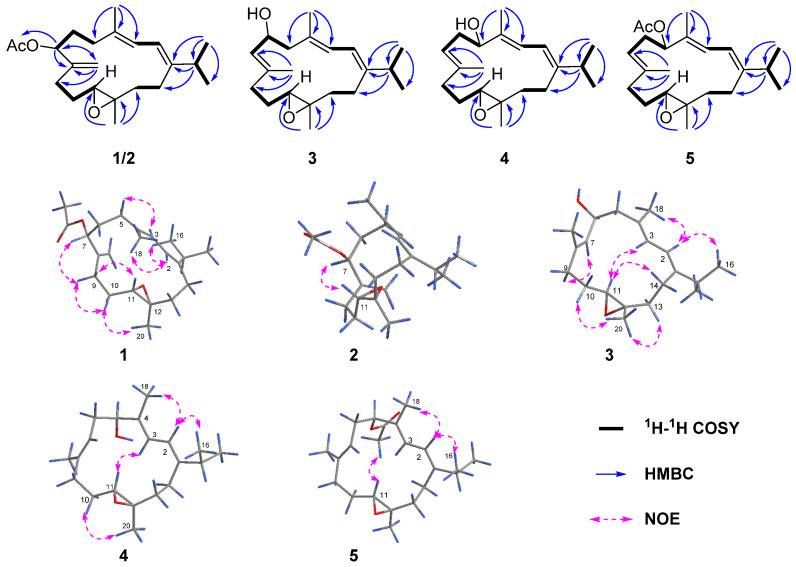
The ^1^H–^1^H COSY, selected key HMBC and ROESY correlations of compounds **1**–**5**.

Compound **1**, namely lobocaline A, was isolated as colorless crystals and gave the molecular formula of C_22_H_34_O_3_ as established by HRESIMS from the protonated molecular ion peak observed at *m*/*z* 347.2580 [M + H]^+^ (calcd. for C_22_H_35_O_3_, 347.2581), implying six degrees of unsaturation. The characteristic peak at ν_max_ 1737 cm^−1^ in its IR spectrum showed the presence of ester carbonyl group, which was further confirmed by the diagnostic ^13^C NMR chemical shift at *δ*_C_ 170.6. The ^1^H and ^13^C NMR data (Table 1 and Table 2) of **1** along with the assistance of DEPT spectrum revealed the presence of two trisubstituted double bonds [*δ*_H_ 6.06 (1H, d, *J* = 10.8 Hz, H-2), *δ*_C_ 118.6 (CH, C-2) and 146.9 (qC, C-1); *δ*_H_ 5.97 (1H, d, *J* = 10.9 Hz, H-3), *δ*_C_ 135.1 (qC, C-4) and 121.7 (CH, C-3)], one exocyclic double bond [*δ*_H_ 5.15 (1H, s, H-19) and *δ*_H_ 5.18 (1H, s, H-19), *δ*_C_ 146.8 (qC, C-8) and 113.0 (CH_2_, C-19)] and one epoxy ring [*δ*_H_ 2.86 (1H, dd, *J* = 7.5, 4.6Hz), *δ*_C_ 61.0 (CH, C-11) and *δ*_C_ 61.6 (qC, C-12)]. The above-mentioned moieties accounted for five of the six degrees of unsaturation, indicating the monocyclic carbon framework for **1**. A comparison of the NMR data of **1** with those of the co-occurring known compound sarcoboettgerol D (**6**), which had been recently isolated from soft coral *Sarcophyton boettgeri* and its absolute configuration was determined by the TDDFT-ECD calculation method [20], which revealed that they were structural analogues, with the only difference being that the hydroxyl group at C-7 in **6** was substituted by an acetyloxy group at C-7 in **1**, in agreement with the 42 mass unit difference in their molecular weights. The position of the acetyloxy group at C-7, as evidenced by the observation of the upfield shift of C-6 from *δ*_C_ 32.9 in **6** to *δ*_C_ 30.0 in **1**. This assignment was further confirmed by strong HMBC correlations (Figure 2) from H-7 (*δ*_H_ 5.16) to C-19 (*δ*_C_ 113.0), C-8 (*δ*_C_ 146.8) and carbonyl carbon (*δ*_C_ 170.6). Thus, the planar structure of **1** was determined as shown in Figure 2.

**Figure 3:**
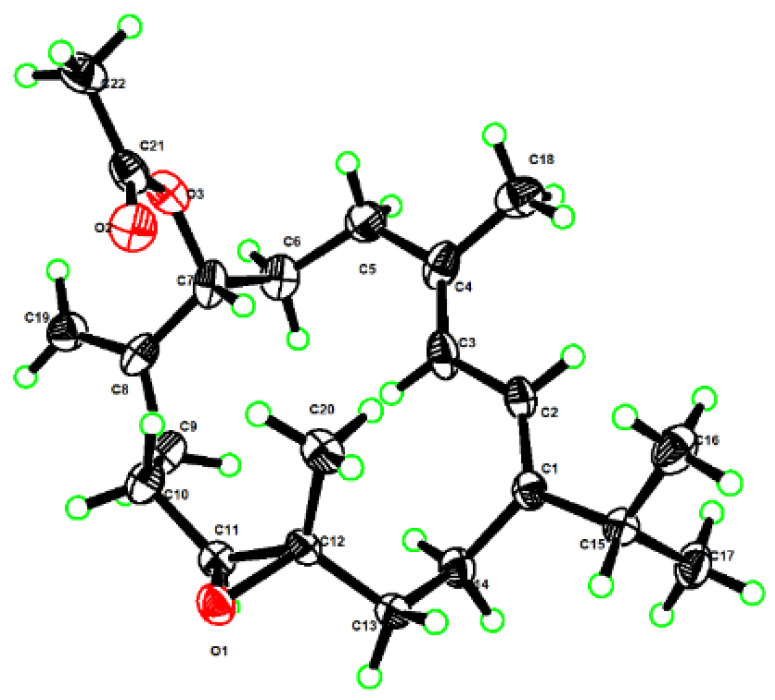
Perspective ORTEP drawing of the X-ray structure of **1** (Carbon atoms are represented by black ellipsoids, oxygen atoms are represented by red ellipsoids, and hydrogen atoms are represented by green spheres).

The relative configuration of **1** was established mainly by the analysis of NOESY correlations (Figure 2). The clear NOE correlations of H-2 (*δ*_H_ 6.06)/H_3_-16 (*δ*_H_ 1.10), H-2/H_3_-18 (*δ*_H_ 1.78) and H-3 (*δ*_H_ 5.97)/H-5*α* (*δ*_H_ 2.24) indicated the “1*E*, 3*E*” geometry of the two double bonds Δ^1,2^ and Δ^3,4^. The highly similar NOE correlations of H-7/H-9*β*, H-9*β*/H-10*β* and H-10*β*/H_3_-20 and H-9α/H-11 between compounds **1** and **6** suggested that these two compounds shared the same stereochemistry. Furthermore, the suitable single crystals of **1** in MeOH were obtained. The X-ray crystallographic analysis using Cu Kα radiation (λ = 1.54178 Å) firmly disclosed the absolute configuration of **1** to be 7*R*, 11*S*, 12*S* with the Flack parameter of 0.00 (10) (Figure 3, CCDC 2290865). What is more is the acetylation of **6** yielding **1** further confirmed the structure and stereo-configuration of **1**. The chemical structure of **1** was thus established as shown in Figure 1.

Lobocaline B (**2**), a colorless oil, gave the same molecular formula as **1** on basis of its HRESIMS ion peak at *m*/*z* 347.2581 [M + H]^+^ (calcd. for C_22_H_35_O_3_, 347.2581). Overall, the ^1^H and ^13^C NMR data of **2** (Table 1 and Table 2) were reminiscent of **1**. Careful comparison of their NMR data revealed they possessed the same planar structure and only chemical shifts at C-7 (δ_C_ 72.5 in **1** vs. δ_C_ 75.9 in **2**) were different, suggesting that **2** was simply the C-7 epi-isomer of **1**, thus suggesting the assignment of 7S*, 11S*, 12S* configuration of **2**. Further analysis of 2D NMR spectra and the obvious NOESY correlations of H-7/H-11 confirmed this hypothesis (Figure 2). Subsequently, the absolute configuration of **2** was then assigned to be 7*S*, 11*S*, 12*S* by the comparison of similar experimental ECD spectra of compounds **1** (Figure S1.9) and **2** (Figure S2.9). Accordingly, the structure of **2** was determined as depicted in Figure 1.

**Figure 4:**
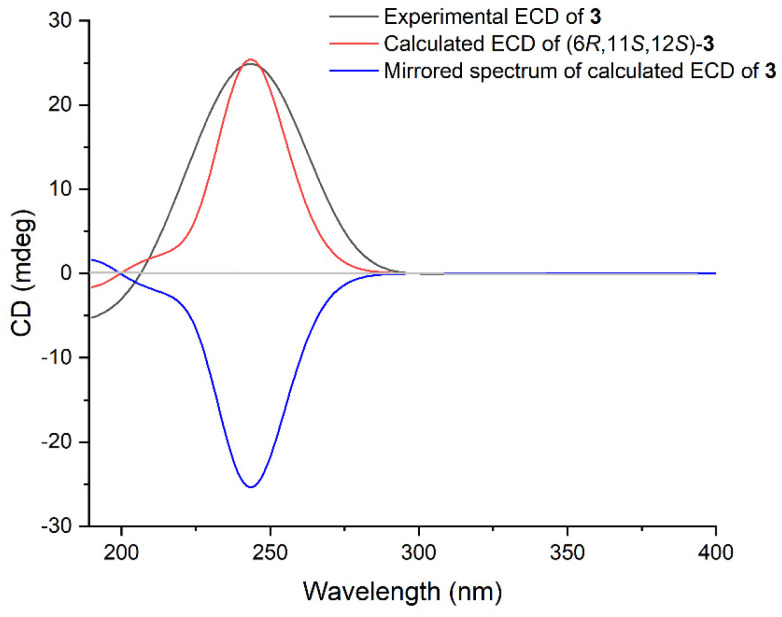
Experimental ECD spectrum of **3** (black), calculated ECD spectra of the enantiomers (6*R*, 11*S*, 12*S*)-**3** (red) and (6*S*, 11*R*, 12*R*)-**3** (blue).

Lobocaline C (**3**) was isolated as a colorless oil, and its molecular formula was assigned as C_20_H_32_O_2_ by HRESIMS ion peak at *m*/*z* 327.2295 [M + Na]^+^ (calcd. for C_20_H_32_O_2_Na, 327.2295), indicating five degrees of unsaturation. The 1D NMR data of **3** (Table 1 and Table 2) showed great similarities to those of l*E*,3*E*,7*E*,11:12-epoxy-l,3,7-cembratriene, a known cembranoid isolated from the South Andaman Coast soft coral *Lobophytum* sp. [26], with the only difference being the presence of an additional hydroxyl group at C-6 in **3**, in agreement with the 16 mass unit difference between these two compounds. This assignment was further confirmed by the strong ^1^H–^1^H COSY correlations of H-6 (δ_H_ 4.54)/H-7(δ_H_ 5.30) (Figure 2). As mentioned above, the planar structure of **3** was determined as shown in Figure 2. The geometries of the double bonds at Δ^1,2^, Δ^3,4^ and Δ^7,8^ were both assigned to be *E* by the NOESY correlations of H-2 (δ_H_ 5.94)/H_3_-16 (δ_H_ 1.05), H-2/H_3_-18 (δ_H_ 1.78) and H-7 (δ_H_ 5.30)/H-9α (δ_H_ 2.30) (Figure 2), respectively. The relative configurations of C-11 and C-12 in **3** were proven to be the same as those of **1** due to the diagnostic NOESY correlations of H_3_-20 (δ_H_ 1.25)/H-10b (δ_H_ 1.46), H_3_-20/H-13b (δ_H_ 2.00), H-11 (δ_H_ 2.82)/H-3 (δ_H_ 5.92) and H-11/H-14a (δ_H_ 2.36). To figure out the relative configuration of **3**, the QM-NMR calculation was performed to give the best match of more than 99% probability in DP4+ with the 6R*, 11S*, 12S* isomer (see the details in the Supplementary Materials). The absolute configuration of **3** was determined by time-dependent density functional theory (TDDFT) ECD calculation. The theoretical ECD spectrum of **3** was calculated by the DFT method at the B3LYP/6-311G(d,p) level. As a result, the Boltzmann-averaged ECD spectrum of (6*R*, 11*S*, 12*S*)-**3** highly matched the experimental ECD spectrum of **3**, while the calculated ECD spectrum of enantiomer showed a completely opposite curve (Figure 4). Consequently, the absolute configuration of **3** was established to be 6*R*, 11*S*, 12*S*.

**Figure 5:**
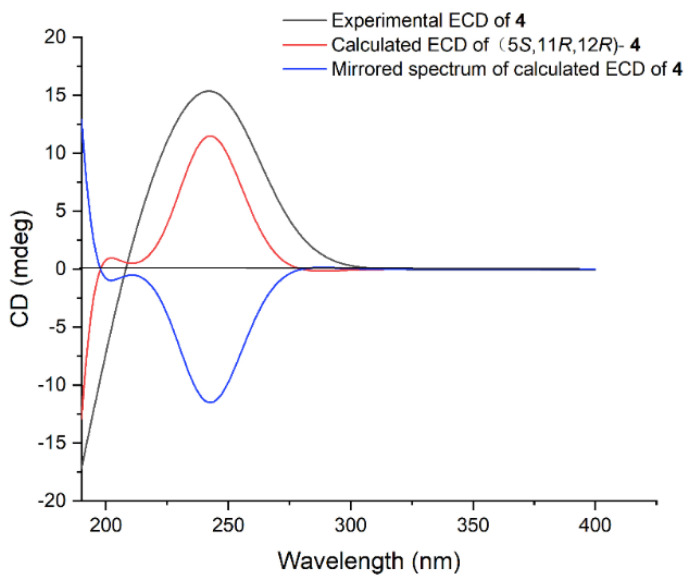
Experimental ECD spectrum of **4** (black), calculated ECD spectra of the enantiomers (5*S*, 11*R*, 12*R*)-**4** (red) and (5*R*, 11*S*, 12*S*)-**4** (blue).

Lobocaline D (**4**), a colorless oil, has the same molecular formula (C_20_H_32_O_2_) as that of **3**, implying they are isomers. Comparison of ^1^H and ^13^C chemical shifts of **4** and **3** (Table 1 and Table 2) followed by a detailed analysis of 2D NMR data revealed that the structure of **4** was similar to that of **3** with a different substituted position of the hydroxyl group. This conclusion was further supported by HMBC correlations from H_3_-18 (δ_H_ 1.74) to C-5 (δ_C_ 78.1) and ^1^H–^1^H COSY correlations of H-5 (δ_H_ 4.12)/H-6α (δ_H_ 2.39)/H-7 (δ_H_ 5.19). Thus, the planar structure of **4** was determined. The relative configurations of C-11 and C-12 in **4** were assigned to be the same as those of **1** due to the similar ^13^C NMR data. Similarly, the relative configuration of **4** was also elucidated via QM-NMR calculations using the DP4+ protocol. The best match was observed for the 5R*, 11S*, 12S* relative configuration with a DP4+ probability over 99%. Moreover, the absolute configuration of **4** was further determined by the TDDFT-ECD approach. As shown in Figure 5, the Boltzmann-averaged ECD curve calculated for the (5*S*, 11*R*, 12*R*)-**4** enantiomer matched very well with the experimental ECD spectrum of **4**. Accordingly, the absolute configuration of **4** was determined to be 5*S*, 11*R*, 12*R*.

**Figure 6:**
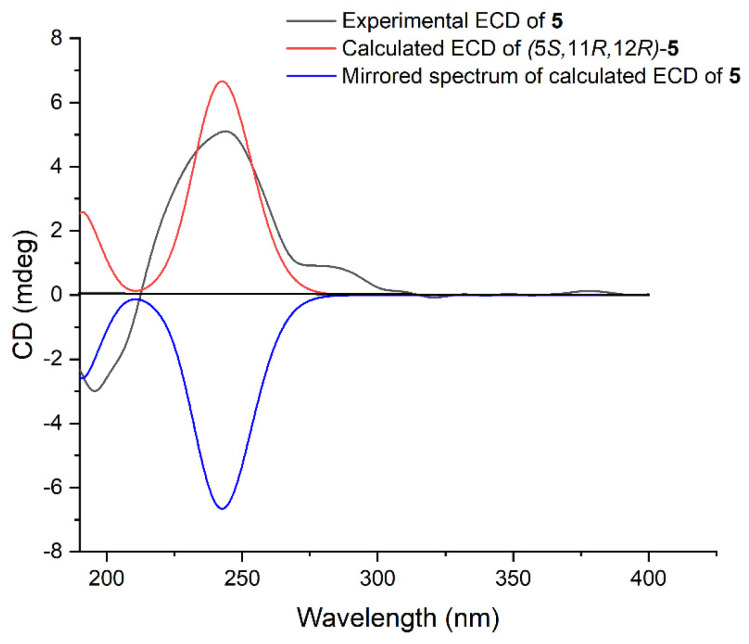
Experimental ECD spectrum of **5** (black), calculated ECD spectra of the enantiomers (5*S*, 11*R*, 12*R*)–**5** (red) and (5*R*, 11*S*, 12*S*)–**5** (blue).

Lobocaline E (**5**) was obtained as a colorless oil. Its molecular formula, C_22_H_34_O_3_, was deduced from the ion peak observed at *m*/*z* 369.2397 [M + Na]^+^ (calcd. for C_22_H_34_O_3_Na, 369.2400) in its HRESIMS spectrum. The ^1^H and ^13^C NMR data (Table 1 and Table 2) of **5** were extremely similar to those of the co-occurring compound **4**, indicating that they are structural analogues. In fact, the only difference between these two compounds is the replacement of the hydroxyl group at C-5 in **4** by the acetyl group at C-5 in **5**, in agreement with the 42 mass unit difference between compounds **4** and **5**. To determine the relative configuration of **5**, the theoretical and experimental NMR data were correlated and their corresponding DP4+ probabilities were estimated. Consequently, the candidate structure **5a** (Figure S11.3) showed the dominant probability of 99.52%, indicating the 5R*, 11S*, 12S* relative configuration of **5**. In this case, the TDDFT-ECD calculation was also employed to determine the absolute stereochemistry of **5**. As shown in Figure 6, the calculated ECD spectrum of (5*S*, 11*R*, 12*R*)-**5** fairly matched with the experimental one, allowing us to assign the absolute configuration of **5** to be 5*S*, 11*R*, 12*R*. Moreover, the hydrolysis reaction of **5** was also performed, but unfortunately, no product was detected due to the low number of isolated compounds. The chemical structure of **5** was thus established as depicted in Figure 1.

**Figure 7:**
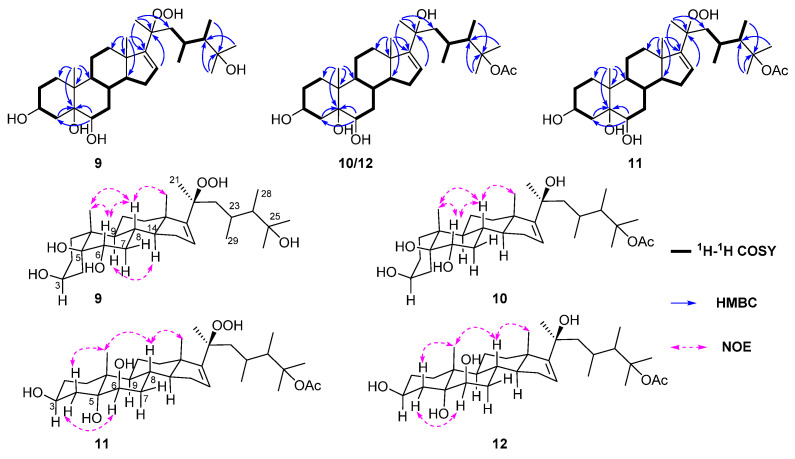
The ^1^H–^1^H COSY, selected key HMBC and NOESY correlations of compounds **9**–**12**.

Lobocaloid A (**9**) was isolated as a white powder. Its molecular formula was determined as C_29_H_50_O_6_ by HRESIMS *m*/*z* 517.3504 [M + Na]^+^ (calcd. for C_29_H_50_O_6_Na, 517.35), appropriate for five degrees of unsaturation. The ^1^H NMR data of **9** (Table 3) showed the general characters of polyhydroxylated sterols, including an olefinic proton resonating at δ_H_ 5.70 (1H, dd, *J* = 3.5, 1.5 Hz, H-16), two oxymethines at δ_H_ 4.27(1H, s, H-3), 3.82 (1H, dd, *J* = 12.2, 4.9 Hz, H-6), a hydroperoxyl group signal at δ_H_ 9.20 (br s), and seven methyls at δ_H_ 0.94 (3H, s, Me-18), 0.95 (3H, s, Me-19), 1.30 (3H, s, Me-21), 1.20 (3H, s, Me-26), 1.19 (3H, s, Me-27), 0.86 (3H, d, *J* = 7.3 Hz, Me-28), 0.93 (3H, d, *J* = 7.1 Hz, Me-29). Moreover, the ^13^C NMR data (Table 3) and DEPT spectrum of **9** indicated the presence of 29 signals, which comprised seven methyls, eight methylenes, eight methines (including one olefinic at δ_C_ 126.9, and seven sp^3^ hybridized at δ_C_ 67.9, 72.0, 32.5, 43.5, 58.0, 30.2, 50.3) and six quaternary carbons (including an olefinic at δ_C_ 158.3 and five sp^3^ hybridized at δ_C_ 78.2, 41.3, 47.5, 85.7, 75.6). These above data suggest the presence of a trisubstituted double bond, which accounted for one degree of unsaturation. The remaining four degrees of unsaturation were attributed to a tetracyclic system in the molecule. In the ^1^H–^1^H COSY spectrum, it was possible to identify three different structural units extending from C-1 to C-4; from C-6 to both C-12 and C-16 through C-8; and from C-22 to both C-28 and C-29 through C-23 (Figure 7). From the HMBC spectrum, the correlations from H_3_-19 to C-1, C-5, C-9 and C-10; from H_3_-18 to C-12, C-13, C-14 and C-17; from H-6 to C-4 and C-5; from H-16 to C-20; from H_3_-21 to C-17, C-20 and C-22; from both H_3_-26 and H_3_-27 to C-24; and from H_3_-28 to C-25 permitted the establishment of the carbon skeleton of a 23,24-dimethycholestane (Figure 7). The hydroperoxyl group substituted at C-20 was confirmed by the HMBC correlation of the hydroperoxyl proton δ_H_ 9.20 (br s) to the oxygenated carbon at δ_C_ 86.1 (C-20). An extensive survey of the literature revealed that the structure of **9** closely resembled that of michosterols A (**16**), a polyoxygenated steroid isolated from the soft coral *Lobophytum michaelae* [27]. A comparison of the NMR data of **9** with **16**, revealed that they were structural analogues, and the only difference occurred at the position C-25, where the acetyloxy group in **16** was replaced by a hydroxyl group in **9**, in agreement with the 42 mass unit difference between compounds **9** and **16**. As mentioned above, the planar structure of **9** was thus established.

The relative configuration of **9** was established mainly by analysis of the NOESY correlations. As depicted in Figure 7, it was found that the NOE interactions displayed by both H_3_-18/H-8, H_3_-19/H-8, H_3_-19/H-6 and H-6/H-8, assuming the β-orientation of H_3_-19, H-6, H-8 and H_3_-18. Moreover, the NOESY correlation of H-9/H-14 assigned the α-orientation of H-9 and H-14. Further, the chemical shifts of C-20, C-21, C-22, C-23, C-28 and C-29 on the side chain are similar to that of **16**, suggested the same relative configuration of the chair centers on the side chain. In addition, the stereochemistry of C-5 and C-6 was assigned to be 5S*, 6S* by the comparison of chemical shifts of C-5 (δ_C_ 78.4) and C-6 (δ_C_ 73.2) with the model known compound 5β-cholestane-3β,5,6α-triol, which was previously isolated from the soft coral *Sinularia* sp. [28,29]. Consequently, the relative configuration of **9** was determined. Furthermore, based on the biogenetic consideration of the biosynthetic pathway of steroids, Me-18 and Me-19 should be positioned on the *β*-orientation; thus, the absolute configuration of **9** was elucidated as shown in Figure 1.

Lobocaloid B (**10**) was isolated as a white powder with the molecular formula of C_31_H_52_O_6_ on the basis of the HRESIMS ion peak at *m*/*z* 543.3655 [M + Na]^+^ (calcd. for C_31_H_52_O_6_Na, 543.3656), corresponding to six degrees of unsaturation. The ^13^C NMR and DEPT spectra of **10** displayed 31 carbon signals, including eight methyls, eight methylenes, eight methines (including one olefinic at δ_C_ 123.6) and seven quaternary carbons (including an olefinic at δ_C_ 161.2 and one carbonyl at δ_C_ 170.6), accounting for two out of six degrees of unsaturation, thus requiring four extra rings in the molecule structure of **10**. The ^13^C NMR data (Table 3) of **10** closely resembled that of michosterols A (**16**), suggesting that they were also structural analogues. In fact, the only difference between **10** and **16** appeared at C-20, where the hydroperoxyl group in **16** was substituted by a hydroxyl group in **10**, in agreement with a 16 mass unit difference between their molecular weights. This assignment was supported by not only the carbon chemical shift of C-20 significantly upfield shifted from δ_C_ 85.6 ppm to δ_C_ 75.7 ppm, but also the chemical shift of the distinctive olefinic proton H-16 shifted from δ_H_ 5.70 (d, *J* = 2.0 Hz) to δ_H_ 5.49 (dd, *J* = 3.4, 1.5 Hz). The relative stereochemistry of **10** was determined by the analysis of NOE correlations (Figure 7) and by comparison of NMR spectroscopic data with those of model molecule sarcophytosterol, a known steroid isolated from the Dongsha atoll soft coral *Lobophytum sarcophytoides*, whose structure has been unambiguously determined by X-ray diffraction analysis [30]. On the basis of the above findings, the structure of compound **10** was determined as shown in Figure 1.

The molecular formula of lobocaloid C (**11**) was the same as michosterols A (**16**), which was determined by HRESIMS ion peak at *m*/*z* 559.3609 [M + Na]^+^ (calcd. for C_31_H_52_O_7_Na, 559.3605). The proton and carbon resonances of **11** showed a high degree of similarity to those of **16**. By comparison of the ^1^H and ^13^C NMR data (Table 4) of **11** and **16**, the differences were found in the chemical shifts of carbons in rings A and B [C-1 (δ_C_ 32.3), C-2 (δ_C_ 31.0), C-4 (δ_C_ 40.8), C-5 (δ_C_ 76.3) and C-6 (δ_C_ 76.2) in **11** and C-1 (δ_C_ 25.2), C-2 (δ_C_ 27.7), C-4 (δ_C_ 30.0), C-5 (δ_C_ 78.1) and C-6 (δ_C_ 71.8) in **16**)]. The following detailed analysis of ^1^H–^1^H COSY and HMBC correlations assigned the planar structure of **11**, the same as **16**, which suggested that **11** was the isomer of **16**. Literature checking revealed that the NMR data of rings A and B in **11** were strongly reminiscent of the known compound 23,24-dimethylcholest-16-ene-3β,5α,6β,11α,20(*R*)-pentol-3-monoacetate [31], indicating that the rings’ structures were identical. Thus, the absolute stereochemistry of C-3, C-5, and C-6 in **11** was speculatively assigned as 3*S*, 5*R*, 6*R*.

Lobocaloid D (**12**), which was obtained as a white powder, gave the molecular formula C_31_H_52_O_6_ on the basis of its HRESIMS ion peak at *m*/*z* 543.3653 [M + Na]^+^ (calcd. for C_31_H_52_O_6_Na, 543.3656), 16 mass units less than that of **11**. A comparison of the NMR data of **12** with **11** (Table 4), revealed that they were structural analogues, with the only difference being the presence of a hydroxyl group substituted at C-20 on the side chain of **12** instead of a hydroperoxyl group in **11**, in agreement with the 16 mass unit difference between compounds **11** and **12**. The hydroxyl group was substituted at C-20, as evidenced by the observation of the upfield shifting of C-20 from δ_C_ 86.1 in **11** to δ_C_ 75.8 in **12**. Similarly, the relative configuration of **12** was determined by analyzing NOE correlations and comparing its NMR data with those of compounds **10** and **11**. As mentioned above, the structure of **12** was assigned as shown in Figure 1.

**Figure 8:**
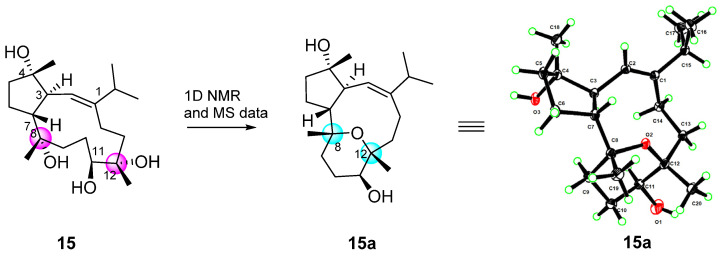
Revised structure (**15a**) and originally structure of lobophytrol B (**15**) (Carbon atoms are represented by black ellipsoids, oxygen atoms are represented by red ellipsoids, and hydrogen atoms are represented by green spheres).

Compound **15** was readily identified as lobophytrol B, a capnosane-type diterpenoid previously reported to be isolated from *Lobophytum* sp. by our group [23]. By comparing the nuclear magnetic and optical rotation data, they proved to be exactly the same. In this study, the suitable crystals of **15** were eventually obtained, and they were then sent for X-ray crystallographic analysis using Cu Ka radiation (λ = 1.54178 Å). Analysis of the X-ray data unambiguously determined the planar structure and absolute configuration of **15** with the Flack parameter of 0.12 (7) (CCDC 2290866). Surprisingly, the X-ray result disclosed that the correct structure of lobophytrol B is **15a**, instead of **15** (Figure 8). Compared to the mass spectrum, the molecular weight is 18 more than before, probably due to the presence of different molecular fragmentation peaks. We carefully and analyzed in depth the data to determine why the structure of **15** was incorrectly assigned, realizing that the error was triggered by the chemical shift of carbon substituted by the hydroxyl group and the same oxygen ring established at positions C-8 and C-12, as well as the existence of various molecular fragment peaks in the mass spectrum. Careful re-examination of the HRESIMS spectrum revealed wrong identification of the molecular ion peak at *m*/*z* HREIMS 363.2517 [M + Na]^+^ (calcd for C_20_H_36_O_4_Na, 363.2506), while the cluster of quasi-molecular ion peaks centered at *m*/*z* 323.2581 [M + H]^+^ (calcd for C_20_H_35_O_3_, 323.2581) was overlooked (Figure S10.1).

In the in vitro bioassay, the isolated compounds were tested for their antibacterial, cytotoxic and anti-inflammatory effects. In the antibacterial bioassays (Table 5), compound **2** showed moderate antibacterial activities against the fish pathogenic bacteria *Streptococcus parauberis* KSP28 (MIC 8.7 μg/mL) and *Phoyobacterium damselae* FP2244 (MIC 17.3 μg/mL). Compounds **7**–**9** showed weak antibacterial activities against *S. parauberis* KSP28 (MIC 30.4, 32.2, 49.4 μg/mL) and *P. damselae* FP2244 (MIC 30.4, 16.1, 49.4 μg/mL). All the steroids exhibited antibacterial activities against *S. parauberis* KSP28 with MIC values ranging from 12.3 to 53.6 µg/mL. Furthermore, compounds **10** and **13** exhibited significant antibacterial activities against a variety of strains; compound **13** especially displayed potent inhibitory activity against *P. damselae* FP2244, *S. parauberis* SPOF3K and *S. agalactiae* WR10 with MIC value of 6.2 µg/mL, 12.3 µg/mL and 12.3 µg/mL, respectively. Furthermore, compound **13** showed the growth inhibitory activities against the vancomycin-resistant *Enterococcus faecium* G1 and G8 with the MIC values of 12.3 μg/mL and 12.3 μg/mL, respectively, comparable with that of positive control levofloxacin hydrochloride (MIC > 39.78 µg/mL), ampicillin sodium (MIC > 37.14 µg/mL) and vancomycin hydrochloride (MIC > 297 µg/mL and 74.25 µg/mL).

**Figure 9:**
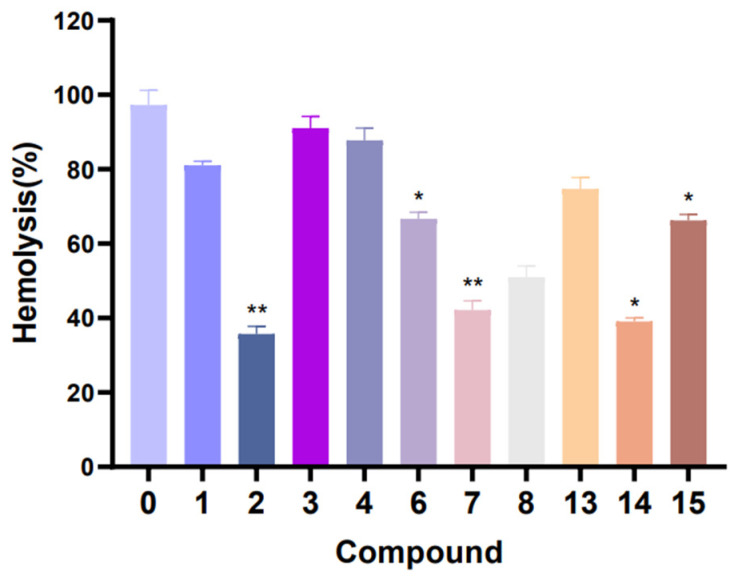
Efficacy of compounds **1**–**4**, **6**–**8** and **13**–**15** in inhibiting the hemolytic activity of *S. aureus*. ** *p* < 0.01, * *p* < 0.05. The assays were independently repeated at least three times.

**Figure 10:**
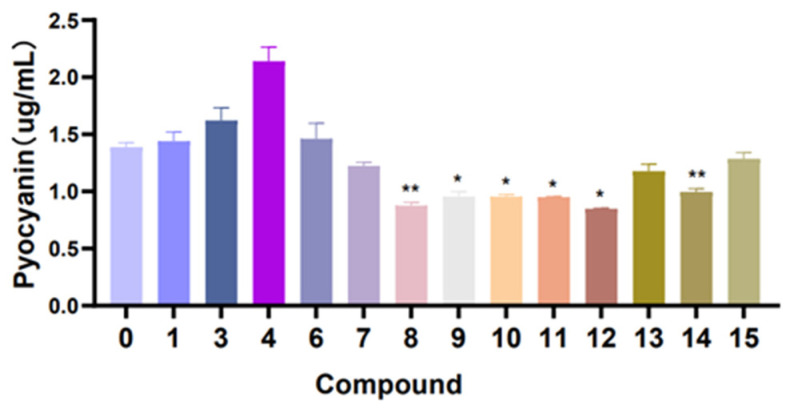
Efficacy of compounds **1**, **3**–**4** and **6**–**15** in the pyocyanin production of *P. aeruginosa*. ** *p* < 0.01, * *p* < 0.05. The assays were independently repeated at least three times.

In hemolytic activity, the results showed that the hemolytic activity of *S. aureus* treated with compounds **1**–**4**, **6**–**8** and **13**–**15** were obviously reduced when compared with the control (without compound treatment). In particular, the hemolysis percentage of compound **2**, **7** and **14** test groups reached about 35.7%, 42.2% and 39.1% (Figure 9). These results suggest that compounds **2**, **7** and **14** have a strong inhibitory effect on the hemolysin production of *S. aureus*. Furthermore, in the pyocyanin quantitation assay experiment (Figure 10), compounds **8**–**12** have medium inhibition effects on the pyocyanin production in *P. aeruginosa*, compared with the control (without compound treatment).

## 3. Materials and Methods

### 3.1. General Experimental Procedures

Melting points were measured on an X-4 digital micro melting point apparatus. Optical rotations were measured on a Perkin-Elmer 241MC polarimeter (PerkinElner, Fremont, CA, USA). IR spectra were measured on a Nicolet 6700 spectrometer (Thermo Scientific, Waltham, MA, USA), and peaks are reported in cm^−1^. NMR spectra were measured in CDCl_3_ with a Bruker DRX 400, 500, 600 and 800 MHz spectrometer (Bruker Biospin AG, Fällanden, Germany). Chemical shifts are reported in parts per million (δ) in CDCl_3_ (δ_H_ reported referred to CHCl_3_ at 7.26 ppm; δ_C_ reported referred to CDCl_3_ at 77.2 ppm), and coupling constants (*J*) are expressed in Hz. HRESIMS spectra were recorded on an Agilent 1290-6545 UHPLC-QTOF mass spectrometer. Commercial silica gel (Qingdao Haiyang Chemical Group Co., Ltd., Qingdao, China, 200–300 and 300–400 mesh) and Sephadex LH-20 gel (Amersham Biosciences, Amersham, UK) were used for column chromatography. Precoated silica gel plates (Yan Tai Zi Fu Chemical Group Co., Yantai, China, G60 F-254) were used for analytical TLC. Reversed-phase (RP) HPLC was performed on an Agilent 1260 series liquid chromatography equipped with a DAD G1315D detector at 210 and 254 nm. A semi-preparative ODS-HG-5 column [5 µm, 250 × 9.4 mm] was employed for the purifications. All solvents used for CC and HPLC were of analytical grade (Shanghai Chemical Reagents Co., Ltd., Shanghai, China) and chromatographic grade (Dikma Technologies Inc., Foothill Ranch, CA, USA), respectively. X-ray crystallographic analysis was carried out on a Bruker D8 Venture diffractometer with Cu Kα radiation (λ = 1.54178 Å) at 100 K. The collected data integration and reduction were processed with SAINT V8.37A software, and multi-scan absorption corrections were performed using the SADABS program. The structure was solved with the SHELXT structure solution program using intrinsic phasing and refined with the SHELXL refinement package using least-squares minimization. Copies of these data can be obtained free of charge via www.ccdc.cam.ac.uk. or from the Cambridge Crystallographic Data Centre, 12 Union Road, Cambridge CB21EZ, UK. [Fax: (+44) 1223-336-033. E-mail: deposit@ccdc.cam.ac.uk].

### 3.2. Animal Materials

Specimens of *L. catalai*, identified by Prof. Xiu-Bao Li from Hainan University, were collected by scuba along the coast off Yalong Bay (18°15′ N, 109°30′ E), Hainan Province, China, in 2006, at a depth of −15 m. A voucher specimen (YAL-65) was deposited and is available for inspection at the Bohai Rim Advanced Research Institute for Drug Discovery.

### 3.3. Extraction and Isolation

The frozen animals (260.5 g, dry weight after extraction) were cut into pieces and extracted exhaustively with acetone at room temperature (2.0 L × 4). The organic extract was evaporated to give a brown residue, which was then partitioned between Et_2_O and H_2_O. The Et_2_O solution was evaporated to give a dark brown residue (4.0 g). The obtained residue was subjected to gradient silica gel (200–300 mesh) column chromatography (CC) [Et_2_O/petroleum ether (PE) 0→100%] and yielded nine fractions (Fr. 1–9). Fr. 2 was divided into three subfractions (Fr. 2A–2C) by Sephadex LH-20 CC (PE/CH_2_Cl_2_/MeOH, 2:1:1). Following two-stage purification including Sephadex LH-20 CC (CH_2_Cl_2_, 100%) and silica gel CC (300–400 mesh, PE/Et_2_O 100:1→10:1), the subfraction Fr. 2C13 was purified by semi-preparative HPLC (MeCN, 100%, 3.0 mL/min) and analytical HPLC (MeOH/H_2_O, 80:20, 1.0 mL/min) to yield compounds **1** (2.0 mg, t_R_ = 16.4 min), **2** (0.3 mg, t_R_ = 18.4 min) and **4** (1.0 mg, t_R_ = 15.3 min), respectively. Fr. 6 was split by Sephadex LH-20 CC (PE/CH_2_Cl_2_/MeOH, 2:1:1) to give two subfractions (Fr. 6A and Fr. 6B). Next, Fr. 6B was purified by Sephadex LH-20 CC (CH_2_Cl_2_, 100%), yielding subfraction Fr. 6BA. Final purification of Fra. 6BA was achieved by semi-preparative HPLC (MeOH/H_2_O, 80:20, 2.8 mL/min) to afford compounds **7** (0.5 mg, t_R_ = 24.5 min) and **6** (1.0 mg, t_R_ = 22.0 min) and the mixture of compounds **3** and **5**. The mixture was further purified by analytical HPLC (MeCN/H_2_O, 60:40, 1.0 mL/min) to yield pure **3** (1.1 mg, t_R_ = 13.0 min) and **5** (1.0 mg, t_R_ = 14.6 min), respectively. Fr. 9 was subjected to a column of Sephadex LH-20 eluted with CH_2_Cl_2_/MeOH, 1:1, to yield two subfractions (Fr. 9A and 9B). Fr. 9A was first split by Sephadex LH-20 column chromatography (PE/CH_2_Cl_2_/MeOH, 2:1:1) to give five subfractions (Fr. 9AA–Fr. 9AE). Fr. 9AE was purified by RP-HPLC (MeCN/H_2_O, 60:40, 3.0 mL/min), yielding a subfraction (Fr. 9AEC, t_R_ = 8.0 min). Since there are two points observed on the thin-layer chromatography (TLC), Fr. 9AEC was purified by silica gel CC (300–400 mesh, CH_2_Cl_2_/MeOH, 96:4) to give compounds **12** (1.0 mg) and **14** (0.5 mg). Fr. 9AD was purified with silica gel CC (300–400 mesh, Et_2_O/PE, 1:1), followed by semi-preparative HPLC (MeCN/H_2_O, 60:40, 3.0 mL/min) to afford **13** (5.4mg, t_R_ = 2.1 min), **11** (3.2mg, t_R_ = 8.8 min) and fraction 9ADFG (t_R_ = 13.7min). In a similar manner, Fr. 9ADFG was purified by silica gel CC (300–400 mesh, CH_2_Cl_2_/MeOH, 98:2) to give compound **10** (2.6 mg). Moreover, compound **9** (2.7 mg, t_R_ = 7.3 min) was obtained from Fr. 9B through Sephadex LH-20 CC (PE/CH_2_Cl_2_/MeOH, 2:1:1) followed by RP-HPLC (MeCN/H_2_O, 60:40, 3.0 mL/min), while compound **15** (2.7 mg, t_R_ = 7.3 min) was obtained from the Fr. 9B through Sephadex LH-20 CC (PE/CH_2_Cl_2_/MeOH, 2:1:1), silica gel CC (200–300 mesh, Et_2_O/PE 50%→100%), followed by RP-HPLC (MeCN/H_2_O, 60:40, 3.0 mL/min).

#### 3.3.1. Lobocaline A (**1**)

Colorless crystal, m.p. 68.9–71.6 °C; ${\left[\alpha \right]}_{D}^{20}$ +97.3 (c 0.20, CHCl_3_); IR (KBr): ν_max_ 3446, 2956, 2924, 2854, 1737, 1458, 1369, 1238, 1026 cm^−1^; For ^1^H and ^13^C NMR spectroscopic data, see Table 1 and Table 2; HRESIMS *m*/*z* 347.2580 [M + H]^+^ (calcd. for C_22_H_35_O_3_, 347.2581).

#### 3.3.2. Lobocaline B (**2**)

Colorless oil, ${\left[\alpha \right]}_{D}^{20}$ +3.9 (c 0.03, MeOH); IR (KBr): ν_max_ 3444, 2956, 2917, 2849, 1731, 1030 cm^−1^; For ^1^H and ^13^C NMR spectroscopic data, see Table 1 and Table 2; HRESIMS *m*/*z* 347.2581 [M + H]^+^ (calcd. for C_22_H_35_O_3_, 347.2581).

#### 3.3.3. Lobocaline C (**3**)

Colorless oil, ${\left[\alpha \right]}_{D}^{20}$ +139.5 (c 0.10, MeOH); IR (KBr): ν_max_ 3438, 2959, 2926, 1457, 1382, 1087, 1022 cm^−1^; For ^1^H and ^13^C NMR spectroscopic data, see Table 1 and Table 2; HRESIMS *m*/*z* 327.2295 [M + Na]^+^ (calcd. for C_20_H_32_O_2_Na, 327.2295).

#### 3.3.4. Lobocaline D (**4**)

Colorless oil, ${\left[\alpha \right]}_{D}^{20}$ +162.7 (c 0.10, CHCl_3_); IR (KBr): ν_max_ 3438, 2959, 2926, 1457, 1382, 1087, 1022 cm^−1^; For ^1^H and ^13^C NMR spectroscopic data, see Table 1 and Table 2; HRESIMS *m*/*z* 327.2293 [M + Na]^+^ (calcd. for C_20_H_32_O_2_Na, 327.2295).

#### 3.3.5. Lobocaline E (**5**)

Colorless oil, ${\left[\alpha \right]}_{D}^{20}$ +17.2 (c 0.10, CHCl_3_); IR (KBr): ν_max_ 3359, 2922, 2851, 1736, 1238, 1141, 1031 cm^−1^; For ^1^H and ^13^C NMR spectroscopic data, see Table 1 and Table 2; HRESIMS *m*/*z* 369.2397 [ M+ Na]^+^ (calcd. for C_22_H_34_O_3_Na, 369.2400).

#### 3.3.6. Lobocaloid A (**9**)

White amorphous powder, ${\left[\alpha \right]}_{D}^{20}$ +9.8 (c 0.27, MeOH); IR (KBr): ν_max_ 3436, 2929, 2871, 1375, 1089, 1050 cm^−1^; For ^1^H and ^13^C NMR spectroscopic data, see Table 3; HRESIMS *m*/*z* 517.3504 [M + Na]^+^ (calcd. for C_29_H_50_O_6_Na, 517.35).

#### 3.3.7. Lobocaloid B (**10**)

White amorphous powder, ${\left[\alpha \right]}_{D}^{20}$ −8.3 (c 0.26, MeOH); IR (KBr): ν_max_ 3444, 2930, 1730, 1369, 1260, 1050, 800 cm^−1^; For ^1^H and ^13^C NMR spectroscopic data, see Table 3; HRESIMS *m*/*z* 543.3655 [M + Na]^+^ (calcd. for C_31_H_52_O_6_Na, 543.3656).

#### 3.3.8. Lobocaloid C (**11**)

White amorphous powder, ${\left[\alpha \right]}_{D}^{20}$ −16.7 (c 0.32, MeOH); IR (KBr): ν_max_ 3444, 2931, 2864, 1374, 1030 cm^−1^; For ^1^H and ^13^C NMR spectroscopic data, see Table 4; HRESIMS *m*/*z* 559.3609 [M + Na]^+^ (calcd. for C_31_H_52_O_7_Na, 559.3605).

#### 3.3.9. Lobocaloid D (**12**)

White amorphous powder; ${\left[\alpha \right]}_{D}^{20}$ −43.3 (c 0.10, MeOH); IR (KBr): ν_max_ 3443, 2928, 2851, 1712, 1368, 1036 cm^−1^; For ^1^H and ^13^C NMR spectroscopic data, see Table 4; HRESIMS *m*/*z* 543.3653 [M + Na]^+^ (calcd. for C_31_H_52_O_6_Na, 543.3656).

### 3.4. Calculation Section

Conformational search was performed by using the torsional sampling (MCMM) approach and OPLS_2005 force field within an energy window of 21 kJ/mol. Conformers above 1% Boltzmann populations were re-optimized at the B3LYP/6-311G(d,p) level with the IEFPCM solvent model for chloroform. Frequency analysis was also carried out to confirm that the re-optimized geometries were at the energy minima. Subsequently, NMR calculations were performed at the PCM/mPW1PW91/6-31G(d) level, as recommended for DP4+. NMR shielding constants were calculated using the GIAO method. Finally, shielding constants were averaged over the Boltzmann distribution obtained for each stereoisomer and correlated with the experimental data. ECD spectra were obtained by TDDFT calculations with the B3LYP/6-311G(d,p) level with the IEFPCM solvent model for CH_3_CN. At last, the Boltzmann-averaged ECD spectra of the compounds were obtained with SpecDis (Version 1.71).

### 3.5. Acetylation of Compound **6**

Compound **6** (0.5 mg) was dissolved in dry pyridine (2.0 mL) and mixed with 50 mL of Ac_2_O. The mixtures were stirred at room temperature overnight, and the reaction was detected on the TLC by heating after spraying with vanillin H_2_SO_4_ reagent. The crude acetylated product, after evaporating the solvent in vacuo, was purified by silica gel CC (petroleum ether/ether, 9:1) to afford a colorless crystal compound **1a** (0.5 mg, 87% yield), which was identical to the natural sample of **1** in all respects (Figure S1.10).

### 3.6. X-ray Crystallographic Analysis for Compounds **1** and **15a**

Lobocaline A (**1**): colorless crystals, m.p. 68.9–71.6 °C; monoclinic, 2(C_22_H_34_O_3_), Mr = 692.98, crystal size: 0.11 × 0.05 × 0.04 mm^3^, space group P2_1_, a = 10.6135(3) Å, b = 16.2226(5) Å, c = 11.8888(4) Å, V = 2044.73(11) Å^3^, Z = 2, ρ_calc_ = 1.126 g/cm^3^, F (000) = 760.0. Independent reflections: 7619 with R_int_ = 0.0385, Rsigma = 0.0475. Final R_1_ = 0.0479, wR_2_ = 0.1174 reflections with I ≥ 2σ (I), R_1_ = 0.0523 (wR_2_ = 0.1215) for all unique data, Flack parameter: 0.00(10). The crystals of **1** were recrystallized from MeOH at room temperature. Crystallographic data for **1** were deposited at the Cambridge Crystallographic Data Centre (Deposition nos. CCDC 2290865).

Lobophytrol B (**15a**): colorless crystals, m.p. 119.1–177.0 °C; monoclinic, C_20_H_36_O_4_, Mr = 340.49, crystal size 0.15 × 0.08 × 0.05 mm^3^, space group I4, a = 14.6887(3) Å, b = 14.6887(3) Å, c = 37.2232(11) Å, V = 8031.2(4) Å^3^, Z = 16, ρ_calc_ = 1.126 g/cm^3^, F(000) = 3008.0, 57940 collected reflections, 8240 independent reflections (R_int_ = 0.0620, Rsigma = 0.0327), final R_1_ = 0.0399 (wR_2_ = 0.1022) reflections with I ≥ 2σ (I), R_1_ = 0.0431, wR_2_ = 0.1051 for all unique data, Flack parameter: 0.12(7). The crystals of **15a** were crystallized from acetone at room temperature. Crystallographic data for **15a** were deposited at the Cambridge Crystallographic Data Centre (Deposition nos. CCDC 2290866).

### 3.7. Antibacterial Activity Bioassays

The strains *Streptococcus parauberis* KSP28, *Streptococcus parauberis* SPOF3K, *Phoyoba cteriumdamselae* FP2244, *Aeromonas salmonicida* AS42, *Photobacterium halotolerans* LMG 22194T, *Enterococcus faecium* 5270 MDR8 and *Lactococcus garvieae* FP MP5245 were provided by National Fisheries Research & Development Institute, Korea. The vancomycin-resistant *Enterococcus faecium* bacteria G1, G4, G7, G8 and G13 were provided by Ruijin Hospital, Shanghai Jiao Tong University School of Medicine. The strain *Streptococcus agalactiae* WR10 and *Edwardsiella piscicida* TH1 were provided by Chinese Academy of Tropical Agricultural Sciences. The MIC values for all antimicrobial agents were measured by the 96-well micro-dilution method. Mueller–Hinton II broth (cation-adjusted, BD 212322) was used for MIC value determination. Generally, compounds were dissolved with DMSO to 20 mM as stock solutions. All samples were diluted with culture broth to 500 µM as the initial concentration. Further 1:2 serial dilutions were performed by addition of culture broth to reach concentrations ranging from 500 µM to 0.24 µM. A total of 100 µL of each dilution was distributed in 96-well plates, as well as sterile controls, growth controls (containing culture broth plus DMSO, without compounds) and positive controls (containing culture broth plus control antibiotics, such as tetracycline). Each test and growth control well was inoculated with 5 µL of an exponential-phase bacterial suspension (about 10^5^ CFU/well). The 96-well plates were incubated at 37 ℃ for 24 h. MIC values of these compounds were defined as the lowest concentration to inhibit the bacterial growth completely. All MIC values were interpreted according to recommendations of the Clinical and Laboratory Standards Institute (CLSI).

### 3.8. Antihemolytic Activity Bioassays

Blood was collected from the eye sockets of SD rats and stood for 30 to 60 min. After centrifugation (800 rpm, 5 min), the red blood cells were cleaned twice with 0.9% normal saline and then added to create a 4% red blood cells suspension. *S. aureus* suspension was incubated with compounds (100 µM) in a centrifuge tube for 12 h at 37 ℃. After centrifugation (6000 rpm, 15 min), 500 µL of the supernatant was taken from each tube, filtered by a 0.2 μm filter membrane and incubated with 500 µL of freshly red blood cells suspension at 37 ℃ for 2 h. The incubation of *S. aureus* and red blood cells suspension was used as the positive control, and the incubation of LB liquid medium and red blood cells suspension served as the negative control. After centrifugation (800 rpm, 5 min), the absorbance of supernatants at 540 nm was examined. The percentage of hemolysis value was calculated by comparing it with the positive control (100% hemolysis).

### 3.9. Pyocyanin Quantitation Assay

The *P. aeruginosa* strain was mixed with the compounds (100 µM) at 37 ℃ and 140 rpm/min for 24 h, and the supernatant was mixed with 1 mL chloroform. Then, the lower chloroform phase was mixed with 200 µL of 0.2 N HCl; after shaking and centrifugal (4500 rpm, 10 min), the color layer was removed and measured at 520 nm. Concentrations, expressed as micrograms of pyocyanin produced per milliliter of culture supernatant, were determined by multiplying the optical density at 520 nm (OD_520_) by 17.072.

## 4. Conclusions

In summary, five new cembrane-type diterpenes lobocalines A–E (**1**–**5**) and four new steroids lobocaloids A–D (**9**–**12**), along with six known analogues (**6**–**8** and **13**–**15**), were isolated and characterized from the soft coral L. catalai collected off Yalong Bay of the South China Sea. Structurally, all the isolated new cembrane-type diterpenes have hydroxyl or acetoxy groups substituted at C-5, C-6 or C-7. All the steroids possessed the hydroxyl groups at C-3, C-5 and C-6, and the stereotypes of hydroxyl groups (3β, 5β, 6α or 3β, 5α, 6β) are significantly different in **9**–**14**. It is worth noting that the structure of known compound **15** has been revised by the X-ray diffraction analysis in this study.

In the bioassays, compounds **2** and **7**–**14** showed antibacterial activities against the fish pathogenic bacteria *S. parauberis* KSP28 with MIC values ranging from 12.3 to 53.6 µg/mL. Compound **13** displayed potent inhibitory activity against *P. damselae* FP2244 with MIC value of 6.2 µg/mL. Compounds **10** (3β, 5β, 6α) and **12** (3β, 5α, 6β) have different configurations, and the antibacterial activity of compound **10** was stronger than that of **12**, suggested the importance of stereochemistry for bioactivity. Further study should be conducted to explore the effect of different cholestane-3,5,6-triols configurations (3β, 5β, 6α or 3β, 5α, 6β) on antibacterial activities. These new findings imply that these isolated compounds could be developed as new chemotypes of lead agents against fish pathogens, which may have a beneficial effect on fish health in the future. In addition, the findings reveal that compounds **2**, **7** and **14** have remarkable inhibition effects on the virulence of *S. aureus*, supporting their potential as the natural antimicrobial agent to combat *S. aureus*.

## Figures and Tables

**Table 1:** ^1^H data of compounds **1**–**5** in CDCl_3_.

No.	1 ^a^	2 ^b^	3 ^b^	4 ^b^	5 ^a^
	*δ*_H_ Mult. (*J* in Hz)	*δ*_H_ Mult. (*J* in Hz)	*δ*_H_ Mult. (*J* in Hz)	*δ*_H_ Mult. (*J* in Hz)	*δ*_H_ Mult. (*J* in Hz)
2	6.06 d (10.8)	5.95 d (10.19)	5.94 d (11.4)	5.92 d (10.1)	5.89 d (10.0)
3	5.97 d (10.9)	5.93 d (10.38)	5.92 d (11.5)	5.98 d (10.1)	6.08 d (10.0)
5*α*	2.24 m	2.13 m	2.26 m	4.12 dd (10.7, 3.5)	5.23 ov ^c^
5*β*	2.14 m	2.33 m	2.44 dd (13.1, 3.2)		
6*α*	2.02 m	1.87 m	4.54 ddd (11.6, 9.0, 3.2)	2.39 ddd (13.5, 10.7, 8.9)	2.53 dt (15.0, 7.5)
6*β*	1.93 ddd (14.4, 10.0, 4.8)	1.83 m		2.31 m	1.27 m
7	5.16 ov	5.22 t (6.8)	5.30 dt (9.0, 1.5)	5.19 t (7.7)	5.25 dd (8.5, 6.3)
9*α*	2.26 m	2.36 m	2.30 m	2.25 m	2.14 m
9*β*	2.23 m	2.06 m	2.21 m	2.12 m	1.38 m
10*α*	1.79 m	1.98 m	1.72 ddt (14.6, 13.1, 3.6)	1.64 m	1.52 m
10*β*	1.65 dp (13.8, 6.9)	1.61 m	1.46 dddd (14.6, 8.3, 4.8, 3.4)	1.60 m	1.81 dq (10.1, 5.9)
11	2.86 dd (7.5, 4.6)	3.05 m	2.82 dd (8.4, 3.6)	2.80 t (6.3)	2.89 dd (7.3, 5.3)
13*α*	2.11 m	2.19 m	1.67 ddd (15.2, 6.6, 4.1)	1.98 ddd (14.8, 8.9, 6.2)	2.10 m
13*β*	1.41 dt (13.4, 5.4)	1.13 m	2.00 ddd (15.0, 10.5, 4.4)	1.64 m	1.42 dt (13.8, 6.2)
14*α*	2.41 ddd (13.8, 9.9, 5.6)	2.24 m	2.36 m	2.26 m	2.10 m
14*β*	2.08 m	2.20 m	1.87 m	1.92 dt (13.1, 6.2)	1.51 m
15	2.33 q (6.8)	2.31 m	2.31 m	2.32 m	2.33 m
16	1.10 d (6.8)	1.05 d (6.8)	1.05 d (6.8)	1.05 d (6.8)	1.04 d (6.9)
17	1.03 d (6.8)	1.03 d (6.9)	1.02 d (6.9)	1.03 d (6.7)	1.02 d (6.9)
18	1.78 s	1.72 s	1.78 s	1.74 s	1.72 s
19*α*	5.15 ov	5.11 s	1.57 s	1.57 s	1.63 s
19*β*	5.18 ov	5.05 s			
20	1.21 s	1.25 s	1.25 s	1.25 s	1.25 s
-OAc	2.05 s	2.07 s			2.06 s

^a^ Recorded at 600 MHz; ^b^ Recorded at 800 MHz; ^c^ Overlapped.

**Table 2:** ^13^C (150 MHz) data of compounds **1**–**5** in CDCl_3_.

No.	1	2	3	4	5
	*δ*_C_, Type	*δ*_C_, Type	*δ*_C_, Type	*δ*_C_, Type	*δ*_C_, Type
1	146.9, C	147.7, C	147.7, C	149.6, C	149.9, C
2	118.6, CH	118.2, CH	118.3, CH	117.9, CH	117.7, CH
3	121.7, CH	121.5, CH	123.4, CH	122.8, CH	123.2, CH
4	135.1, C	135.6, C	132.4, C	136.4, C	133.0, C
5	34.9, CH_2_	34.0, CH_2_	48.5, CH_2_	78.1, CH	77.1, CH
6	30.0, CH_2_	31.1, CH_2_	67.6, CH	34.2, CH_2_	30.1, CH_2_
7	72.5, CH	75.9, CH	130.2, CH	122.3, CH	120.5, CH
8	146.8, C	147.6, C	137.4, C	135.8, C	136.6, C
9	31.5, CH_2_	28.8, CH_2_	37.1, CH_2_	36.9, CH_2_	37.1, CH_2_
10	26.8, CH_2_	28.0, CH_2_	24.6, CH_2_	24.8, CH_2_	24.9, CH_2_
11	61.0, CH	62.4, CH	59.1, CH	59.5, CH	60.5, CH
12	61.6, C	61.7, C	61.0, C	60.9, C	61.4, C
13	37.1, CH_2_	38.8, CH_2_	34.0, CH_2_	35.0, CH_2_	37.0, CH_2_
14	26.0, CH_2_	26.1, CH_2_	23.4, CH_2_	24.2, CH_2_	25.4, CH_2_
15	33.2, CH	34.2, CH	32.5, CH	33.2, CH	34.1, CH
16	21.5, CH_3_	22.2, CH_3_	22.3, CH_3_	22.2, CH_3_	22.2, CH_3_
17	23.1, CH_3_	22.3, CH_3_	23.0, CH_3_	22.8, CH_3_	22.3, CH_3_
18	16.7, CH_3_	17.8, CH_3_	17.6, CH_3_	11.3, CH_3_	14.5, CH_3_
19	113.0, CH_2_	111.9, CH_2_	15.7, CH_3_	15.1, CH_3_	15.5, CH_3_
20	18.8, CH_3_	17.2, CH_3_	19.4, CH_3_	18.9, CH_3_	18.0, CH_3_
OAc	170.6, C	170.4, C			170.5, C
	21.4, CH_3_	21.3, CH_3_			21.5, CH_3_

**Table 3:** ^1^H (600 MHz) and ^13^C (150 MHz) NMR data of compounds **9** and **10** in CDCl_3_.

No.	9		10	
	*δ*_C_, Type	*δ*_H_ Mult (*J* in Hz)	*δ*_C_, Type	*δ*_H_ Mult (*J* in Hz)
1	25.4, CH_2_	1.35 m	25.4, CH_2_	1.36 m
		1.81 m		1.83 m
2	27.9, CH_2_	1.65 m	28.0, CH_2_	2.09 m
		1.56 m		1.58 m
3	67.9, CH	4.27 br s	67.9, CH	4.28 br s
4	30.1, CH_2_	1.97 m	30.1, CH_2_	1.85 m
		1.84 m		1.96 m
5	78.2, C		78.2, C	
6	72.0, CH	3.82 dd (12.2, 4.9)	72.0, CH	3.82 dd (12.2, 4.9)
7	34.7, CH_2_	1.85 m	34.7, CH_2_	1.86 m
		1.07 m		1.07 m
8	32.5, CH	1.77 m	32.6, CH	1.76 m
9	43.5, CH	1.35 m	43.6, CH	1.30 m
10	41.3, C		41.3, C	
11	21.7, CH_2_	1.49 m	21.8, CH_2_	1.48 m
		1.40 m		1.53 m
12	36.2, CH_2_	2.08 m	36.5, CH_2_	2.11 m
		1.67 m		1.53 m
13	47.5, C		47.9, C	
14	58.0, CH	1.51 m	57.9, CH	1.47 m
15	31.2, CH_2_	1.87 m	31.1, CH_2_	1.83 m
		2.11 m		2.07 m
16	126.9, CH	5.70 dd (3.5, 1.5)	123.6, CH	5.49 dd (3.4, 1.5)
17	158.3, C		161.2, C	
18	18.1, CH_3_	0.94 s	18.0, CH_3_	0.96 s
19	19.4, CH_3_	0.95 s	18.1, CH_3_	0.96 s
20	85.7, C		75.7, C	
21	23.0, CH_3_	1.30 s	29.3, CH_3_	1.39 s
22	46.7, CH_2_	2.04 m	50.6, CH_2_	1.65 m
		1.48 m		1.40 m
23	25.2, CH	1.36 m	27.9, CH	2.10 m
24	50.3, CH	1.70 m	47.3, CH	1.99 m
25	75.6, C		86.6, C	
26	30.2, CH_3_	1.20 s	22.9, CH_3_	1.98 s
27	25.2, CH_3_	1.19 s	23.8, CH_3_	1.46 s
28	10.8, CH_3_	0.86 d (7.3)	8.8, CH_3_	0.85 d (7.2)
29	17.3, CH_3_	0.93 d (7.1)	17.3, CH_3_	0.90 d (6.9)
OAc			170.6, C	
			25.1, CH_3_	1.50 s
OOH		9.20 br s		

**Table 4:** ^1^H (600 MHz) and ^13^C (150 MHz) NMR data of compounds **11** and **12** in CDCl_3_.

No.	11		12	
	*δ*_C_, Type	*δ*_H_ Mult (*J* in Hz)	*δ*_C_, Type	*δ*_H_ Mult (*J* in Hz)
1	32.3, CH_2_	1.56 m	32.4, CH_2_	1.53 m
		1.42 m		1.42 m
2	31.0, CH_2_	1.86 m	31.0, CH_2_	1.88 m
		1.52 m		1.52 m
3	67.7, CH	4.11td (10.8,5.3)	67.7, CH	4.11 td (10.9, 5.3)
4	40.8, CH_2_	2.10 m	40.9, CH_2_	2.10 m
		1.62 m		1.64 m
5	76.3, C		76.4, C	
6	76.2, CH	3.57 t (3.1)	76.2, CH	3.60 t (2.9)
7	34.4, CH_2_	1.70 m	34.5, CH_2_	1.73 ddd (14.1, 12.5, 3.7)
		1.61 m		1.63 m
8	29.0, CH	1.98 m	29.0, CH	1.96 m
9	46.1, CH	1.34 m	46.2, CH	1.32 td (11.4, 5.0)
10	38.6, C		38.7, C	
11	21.3, CH_2_	1.45 m	21.3, CH_2_	1.41 m
		1.37 m		1.45 m
12	36.1, CH_2_	1.67 m	36.5, CH_2_	1.58 m
		2.07 m		2.09 m
13	47.6, C		47.9, C	
14	57.7, CH	1.52 m	57.5, CH	1.45 m
15	31.1, CH_2_	2.13 m	31.0, CH_2_	2.07 m
		1.90 m		1.90 m
16	127.1, CH	5.70 dd (3.5, 1.5)	123.7, CH	5.49 dd (3.4, 1.5)
17	157.8, C		161.2, C	
18	18.3, CH_3_	0.98 s	18.4, CH_3_	1.00 s
19	16.9, CH_3_	1.21 s	16.9, CH_3_	1.22 s
20	86.1, C		75.8, C	
21	22.9, CH_3_	1.32 s	29.3, CH_3_	1.39 s
22	44.6, CH_2_	1.96 m	50.5, CH_2_	1.62 m
		1.43 m		1.43 m
23	26.6, CH	2.07 m	28.0, CH	2.10 m
24	46.4, CH	2.23 qd (7.6, 1.9)	47.3, CH	1.95 m
25	87.2, C		86.6, C	
26	24.3, CH_3_	1.42 s	23.9, CH_3_	1.47 s
27	25.8, CH_3_	1.49 s	25.1, CH_3_	1.49 s
28	9.4, CH_3_	0.88 d (7.2)	8.8, CH_3_	0.85 d (7.1)
29	18.1, CH_3_	0.93 d (6.9)	17.9, CH_3_	0.90 d (6.9)
OAc	171.9, C		170.6, C	
	22.7, CH_3_	2.02 s	22.9, CH_3_	1.98 s
OOH		7.89 br s		

**Table 5:** The MIC values (μg/mL) of antibacterial bioassays of compounds **2** and **7**–**14**.

Compounds	2	7	8	9	10	11	12	13	14	TC ^a^	OT	LF	AMP	VAN
*Streptococcus parauberis* KSP28	8.7	30.4	32.2	49.4	13.0	53.6	26.0	12.3	50.8	3.01	1.55	1.24	4.64	
*Enterococcus faecium* 5270 MDR8	- ^b^	-	-	-	-	-	-	49.2	-	>48.09	>49.69	4.98	>37.14	
*Aeromonas salmonicida* AS42	-	-	-	-	26.0	-	-	24.6		6.11	0.39	0.31	>18.57	
*Photobacterium halotolerans* LMG 22194T	-	-	-	-	-	-	-	24.6	-	0.19	0.39	0.16	0.04	
*Streptococcus parauberis* SPOF3K	-	-	-	-	26.0	-	26.0	12.3	50.8	>24.05	12.42	1.24	0.58	
*Lactococcus garvieae* FP MP5245	-	-	-	-	52.0	-	-	24.6	-	0.38	0.19	0.62	0.58	
*Phoyobacterium damselae* FP2244	17.3	30.4	16.1	49.4	13.0	-	26.0	6.2	-	0.02	0.02	0.02	0.02	
*Enterococcus faecium* G1	-	-	-	-	52.0	-	-	12.3	-	0.09	0.19	>39.78	>37.14	>297
*Enterococcus faecium* G4	-	-	-	-	52.0	-	-	49.2	-	0.19	0.19	>39.78	>37.14	>297
*Enterococcus faecium* G7	-	-	-	-	26.0	53.6	52.0	24.6	-	0.09	0.09	>39.78	>37.14	18.56
*Enterococcus faecium* G8	-	-	-	-	52.0	-	52.0	12.3	-	0.09	0.05	39.78	>37.14	74.25
*Enterococcus faecium* G13	-	-	-	-	52.0	-	-	49.2	-	12.00	6.20	>39.78	>37.14	>297
*Streptococcus agalactiae* WR10	-	-	-	-	26.0	-	52.0	12.3	-	0.75	0.78	2.49	1.16	<0.2
*Edwardsiella piscicida* TH1	-	-	-	49.4	-	-	52.0	-	-	1.50	0.78	0.62	9.28	>148

^a^ Tetracycline hydrochloride (TC), oxytetracycline hydrochloride (OT), levofloxacin hydrochloride (LF), Ampicillin (AMP) and Vancomycin hydrochloride (VAN) were used as positive controls. ^b^ ‘-’ indicated they were not subjected to the antibacterial rescreening experiments since their inhibition rates against these bacteria were <90% in the preliminary antibacterial bioassays.

## Data Availability

Data are contained within the article or Supplementary Materials.

## Supplementary Materials

The following supporting information can be downloaded at: https://www.mdpi.com/article/10.3390/md22010050/s1. X-ray crystallographic analyses data of compounds **1** and **15a**, NMR, HR-ESIMS and IR data of compounds **1**–**5** and **9**–**12**, CD and UV data of compounds **1**–**5**, HR-ESIMS data of compound **15a**, QM-NMR and TDDFT-ECD calculation data of compound **3**–**5**.
