# Supplementary material for: Nine New Antibacterial Diterpenes and Steroids from the South China Sea Soft Coral Lobophytum catalai Tixier-Durivault
Source: Mar Drugs. 2024 Jan 20;22(1):50. doi: 10.3390/md22010050 (PMC10817416; doi:10.3390/md22010050)
Supplement: Supplementary file 1 [file marinedrugs-22-00050-s001.zip › marinedrugs-2821795-supplementary.pdf]

Supplementary Material

Nine New Antibacterial Diterpenes and Steroids from the South China Sea soft coral *Lobophytum catalai* Tixier-Durivault

Sheng-Hui Zhu[2,3], Yuan-Min Chang[2], Ming-Zhi Su[2], Li-Gong Yao[2], Song-Wei Li[1,*], Hong Wang[3,*] and Yue-Wei Guo[1,2,3,*]

[1] School of Medicine, Shanghai University, Shanghai 200444, China

[2] Shandong Laboratory of Yantai Drug Discovery, Bohai Rim Advanced Research Institute for Drug Discovery, Yantai 264117, China; zhushenghui0115@163.com (S.-H. Z.); 13153672940@163.com (Y.-M.C.) yaoligong@simm.ac.cn (L.-G.Y.); smz0310@163.com (M.-Z.S.)

[3] Collaborative Innovation Center of Yangtze River Delta Region Green Pharmaceuticals and College of Pharmaceutical Science, Zhejiang University of Technology, Hangzhou 310014, China

* Correspondence: simmswli@163.com (S.-W. L.); hongw@zjut.edu.cn (H. W.); ywguo@simm.ac.cn (Y.-W. G.)

[*]Corresponding author.

E-mail addresses: ywguo@simm.ac.cn

Table of Contents

1. X-ray crystallographic analyses 3

2. Spectra of compound 1. 4

Figure S1.1 ^{1}H NMR spectrum (600 MHz) of compound **1** in $CDCl_3$. 4
Figure S1.2 ^{13}C NMR spectrum (150 MHz) of compound **1** in $CDCl_3$ 4
Figure S1.3 HSQC spectrum (600 MHz) of compound **1** in $CDCl_3$.......................5
Figure S1.4 HMBC spectrum (600 MHz) of compound **1** in $CDCl_3$.....................5
Figure S1.5 ^{1}H-^{1}H COSY spectrum (600 MHz) of compound **1** in $CDCl_3$ 6
Figure S1.6 NOESY spectrum (600 MHz) of compound **1** in $CDCl_3$ 6
Figure S1.7 HR-ESIMS spectrum of compound **1**. 7
Figure S1.8 IR spectrum of compound **1**. 7
Figure S1.9 CD and UV spectra of compound **1**. 8
Figure S1.10 ^{1}H NMR spectrum (400 MHz) of compound **1a** 8

3. Spectra of compound 2. 9

Figure S2.1 ^{1}H NMR spectrum (800 MHz) of compound **2** in $CDCl_3$. 9
Figure S2.2 ^{13}C NMR spectrum (150 MHz) of compound **2** in $CDCl_3$ 9
Figure S2.3 HSQC spectrum (800 MHz) of compound **2** in $CDCl_3$....................10
Figure S2.4 HMBC spectrum (800 MHz) of compound **2** in $CDCl_3$...................10
Figure S2.5 ^{1}H-^{1}H COSY spectrum (800 MHz) of compound **2** in $CDCl_3$ 11
Figure S2.6 NOESY spectrum (800 MHz) of compound **2** in $CDCl_3$ 11
Figure S2.7 HR-ESIMS spectrum of compound **2**. 12
Figure S2.8 IR spectrum of compound **2**. 12
Figure S2.9 CD and UV spectra of compound **2**. 12

4. Spectra of compound 3. 13

Figure S3.1 ^{1}H NMR spectrum (600 MHz) of compound **3** in $CDCl_3$. 13
Figure S3.2 ^{13}C NMR spectrum (150 MHz) of compound **3** in $CDCl_3$............ 13
Figure S3.3 HSQC spectrum (600 MHz) of compound **3** in $CDCl_3$....................14
Figure S3.4 HMBC spectrum (600 MHz) of compound **3** in $CDCl_3$...................14
Figure S3.5 ^{1}H-^{1}H COSY spectrum (600 MHz) of compound **3** in $CDCl_3$ 15
Figure S3.6 NOESY spectrum (600 MHz) of compound **3** in $CDCl_3$ 15
Figure S3.7 HR-ESIMS spectrum of compound **3**. 16
Figure S3.8 IR spectrum of compound **3**. 16
Figure S3.9 CD and UV spectrum of compound **3**. 16

5. Spectra of compound 4. 17

Figure S4.1 ^{1}H NMR spectrum (800 MHz) of compound **4** in $CDCl_3$. **17**
Figure S4.2 ^{13}C NMR spectrum (150 MHz) of compound **4** in $CDCl_3$ **17**
Figure S4.3 HSQC spectrum (800 MHz) of compound **4** in $CDCl_3$ **18**
Figure S4.4 HMBC spectrum (800 MHz) of compound **4** in $CDCl_3$ **18**
Figure S4.5 ^{1}H-^{1}H COSY spectrum (800 MHz) of compound **4** in $CDCl_3$ **19**
Figure S4.6 NOESY spectrum (800 MHz) of compound **4** in $CDCl_3$ **19**
Figure S4.7 HR-ESIMS spectrum of compound **4**. **20**
Figure S4.8 IR spectrum of compound **4**. **20**
Figure S4.9 CD and UV spectrum of compound **4**. **20**

6. Spectra of compound 5. 21

Figure S5.1 ^{1}H NMR spectrum (600 MHz) of compound **5** in $CDCl_3$. **21**
Figure S5.2 ^{13}C NMR spectrum (150 MHz) of compound **5** in $CDCl_3$ **21**
Figure S5.3 HSQC spectrum (600 MHz) of compound **5** in $CDCl_3$ **22**
Figure S5.4 HMBC spectrum (600 MHz) of compound **5** in $CDCl_3$ **22**
Figure S5.5 ^{1}H-^{1}H COSY spectrum (600 MHz) of compound **5** in $CDCl_3$ **23**
Figure S5.6 NOESY spectrum (600 MHz) of compound **5** in $CDCl_3$ **23**
Figure S5.7 HR-ESIMS spectrum of compound **5**. **24**
Figure S5.8 IR spectrum of compound **5**. **24**
Figure S5.9 CD and UV spectrum of compound **5**. **24**

7. Spectra of compound 9. 25

Figure S6.1 ^{1}H NMR spectrum (600 MHz) of compound **9** in $CDCl_3$. **25**
Figure S6.2 ^{13}C NMR spectrum (150 MHz) of compound **9** in $CDCl_3$ **25**
Figure S6.3 HSQC spectrum (600 MHz) of compound **9** in $CDCl_3$ **26**
Figure S6.4 HMBC spectrum (600 MHz) of compound **9** in $CDCl_3$ **26**
Figure S6.5 ^{1}H-^{1}H COSY spectrum (600 MHz) of compound **9** in $CDCl_3$ **27**
Figure S6.6 NOESY spectrum (600 MHz) of compound **9** in $CDCl_3$ **27**
Figure S6.7 HR-ESIMS spectrum of compound **9**. **28**
Figure S6.8 IR spectrum of compound **9**. **28**

8. Spectra of compound 10. 29

Figure S7.1 ^{1}H NMR spectrum (600 MHz) of compound **10** in $CDCl_3$. **29**
Figure S7.2 ^{13}C NMR spectrum (150 MHz) of compound **10** in $CDCl_3$ **29**
Figure S7.3 HSQC spectrum (600 MHz) of compound **10** in $CDCl_3$ **30**
Figure S7.4 HMBC spectrum (600 MHz) of compound **10** in $CDCl_3$ **30**
Figure S7.5 ^{1}H-^{1}H COSY spectrum (600 MHz) of compound **10** in $CDCl_3$ **31**
Figure S7.6 NOESY spectrum (600 MHz) of compound **10** in $CDCl_3$ **31**
Figure S7.7 HR-ESIMS spectrum of compound **10**. **32**
Figure S7.8 IR spectrum of compound **10**. **32**

9. Spectra of compound 11. 33

Figure S8.1 ^{1}H NMR spectrum (600 MHz) of compound **11** in $CDCl_3$. **33**
Figure S8.2 ^{13}C NMR spectrum (150 MHz) of compound **11** in $CDCl_3$ **33**

Figure S8.3 HSQC spectrum (600 MHz) of compound **11** in $CDCl_3$ 34
Figure S8.4 HMBC spectrum (600 MHz) of compound **11** in $CDCl_3$ 34
Figure S8.5 ^{1}H-^{1}H COSY spectrum (600 MHz) of compound **11** in $CDCl_3$ 35
Figure S8.6 NOESY spectrum (600 MHz) of compound **11** in $CDCl_3$ 35
Figure S8.7 HR-ESIMS spectrum of compound **11**. 36
Figure S8.8 IR spectrum of compound **11**. 36

10. Spectra of compound 12. 37

Figure S9.1 ^{1}H NMR spectrum (600 MHz) of compound **12** in $CDCl_3$. 37
Figure S9.2 ^{13}C NMR spectrum (150 MHz) of compound **12** in $CDCl_3$ 37
Figure S9.3 HSQC spectrum (600 MHz) of compound **12** in $CDCl_3$ 38
Figure S9.4 HMBC spectrum (600 MHz) of compound **12** in $CDCl_3$ 38
Figure S9.5 ^{1}H-^{1}H COSY spectrum (600 MHz) of compound **12** in $CDCl_3$ 39
Figure S9.6 NOESY spectrum (600 MHz) of compound **12** in $CDCl_3$ 39
Figure S9.7 HR-ESIMS spectrum of compound **12**. 40
Figure S9.8 IR spectrum of compound **12**. 40

11. Spectra of compound 15a. 40

Figure S10.1 HR-ESIMS spectrum of compound **15a**. 40

12. QM-NMR calculation 41

Figure S11.1 Two possible configurations of compound **3**. 41
Figure S11.2 Two possible configurations of compound **4**. 42
Figure S11.3 Two possible configurations of compound **5**. 44

13. TDDFT-ECD calculation 45

1. X-ray crystallographic analyses

Table S1. X-ray crystallographic data for **1**.

Empirical formula	$C_{44}H_{68}O_6$
Formula weight	692.98
Temperature/K	100
Crystal system	monoclinic
Space group	$P2_1$
a/Å	10.6135(3)
b/Å	16.2226(5)
c/Å	11.8888(4)
α/°	90
β/°	92.695(2)
γ/°	90
Volume/Å^3	2044.73(11)
Z	2
ρ_{calc}g/cm^3	1.126
μ/mm^{-1}	0.570
F(000)	760.0
Crystal size/mm^3	0.11 × 0.05 × 0.04
Radiation	CuKα (λ = 1.54178)
2Θ range for data collection/°	9.228 to 149.386
Index ranges	-12 ≤ h ≤ 12, -19 ≤ k ≤ 19, -14 ≤ l ≤ 14
Reflections collected	15315
Independent reflections	7619 [R_{int} = 0.0385, R_{sigma} = 0.0475]
Data/restraints/parameters	7619/1/461
Goodness-of-fit on F^2	1.031
Final R indexes [I>=2σ (I)]	R_1 = 0.0479, wR_2 = 0.1174
Final R indexes [all data]	R_1 = 0.0523, wR_2 = 0.1215
Largest diff. peak/hole / e Å^{-3}	0.89/-0.27
Flack parameter	0.00(10)

Table S2. X-ray crystallographic data for **15a**.

Empirical formula	$C_{20}H_{36}O_4$
Formula weight	340.49
Temperature/K	100.0
Crystal system	tetragonal
Space group	I4
a/Å	14.6887(3)
b/Å	14.6887(3)
c/Å	37.2232(11)
α/°	90
β/°	90
γ/°	90
Volume/Å^3	8031.2(4)
Z	16
ρ_{calc}g/cm^3	1.126
μ/mm^{-1}	0.605
F(000)	3008.0
Crystal size/mm^3	0.15 × 0.08 × 0.05
Radiation	CuKα (λ = 1.54178)
2Θ range for data collection/°	4.748 to 149.44
Index ranges	-18 ≤ h ≤ 18, -18 ≤ k ≤ 18, -46 ≤ l ≤ 46
Reflections collected	57940
Independent reflections	8240 [R_{int} = 0.0620, R_{sigma} = 0.0327]
Data/restraints/parameters	8240/15/462
Goodness-of-fit on F^2	1.067
Final R indexes [I>=2σ (I)]	R_1 = 0.0399, wR_2 = 0.1022
Final R indexes [all data]	R_1 = 0.0431, wR_2 = 0.1051
Largest diff. peak/hole / e Å^{-3}	0.23/-0.47
Flack parameter	0.12(7)

2. Spectra of compound 1

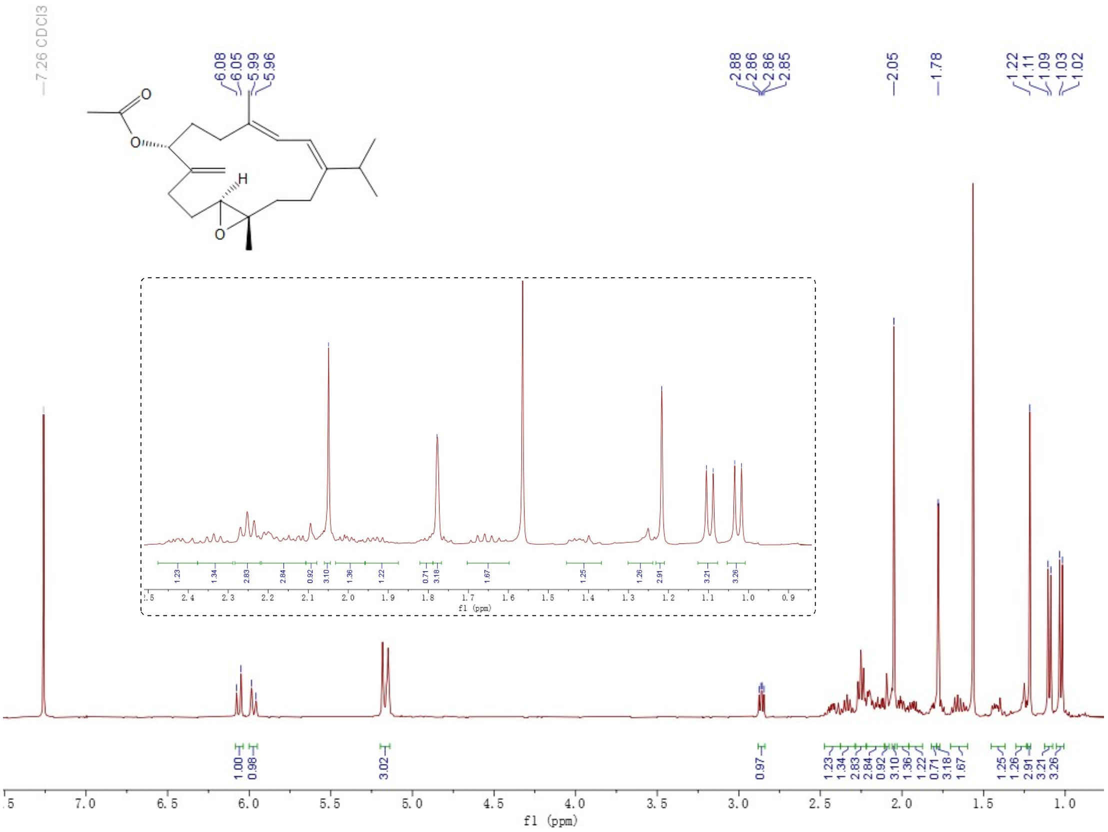

Figure S1.1. ^{1}H NMR spectrum (600 MHz) of compound **1** in $CDCl_3$.

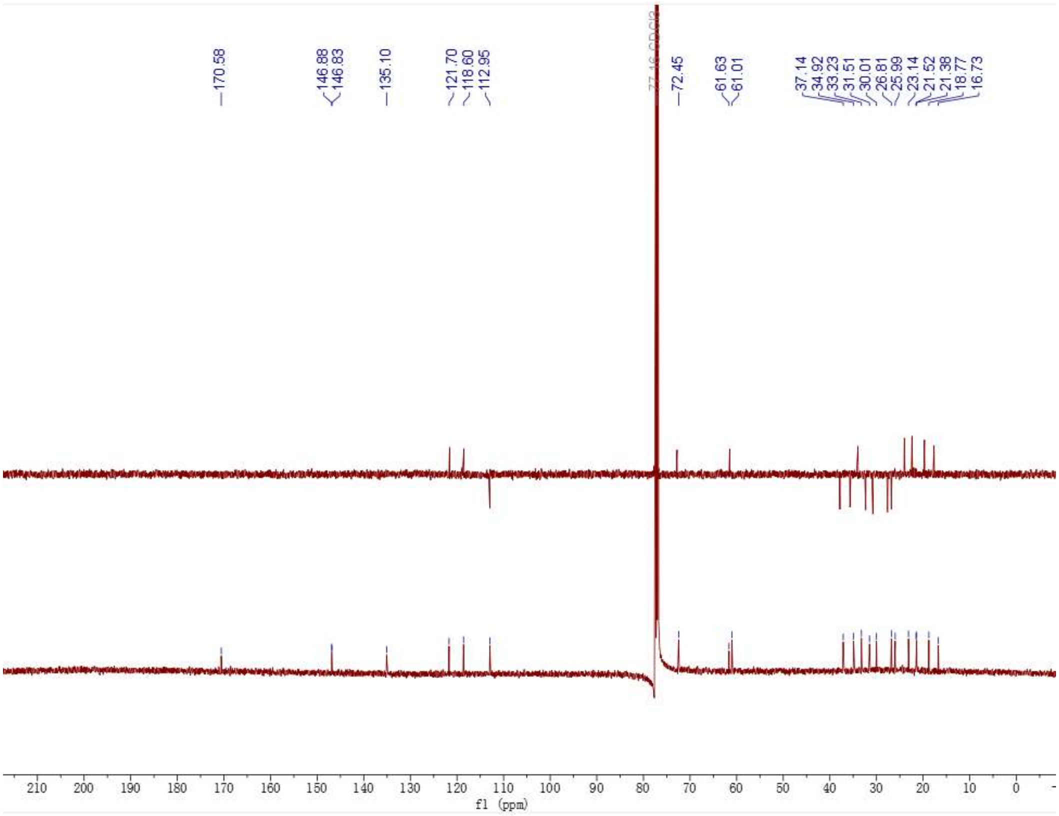

Figure S1.2. ^{13}C NMR spectrum (150 MHz) of compound **1** in $CDCl_3$.

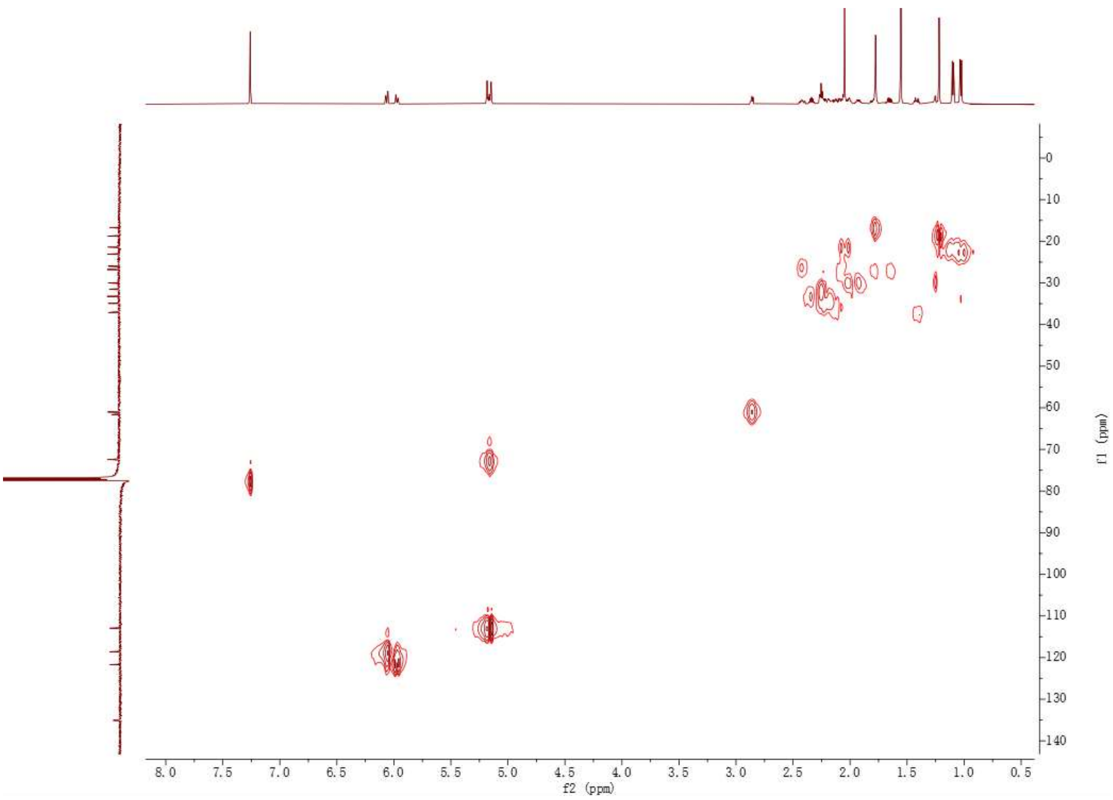

Figure S1.3. HSQC spectrum (600 MHz) of compound **1** in $CDCl_3$.

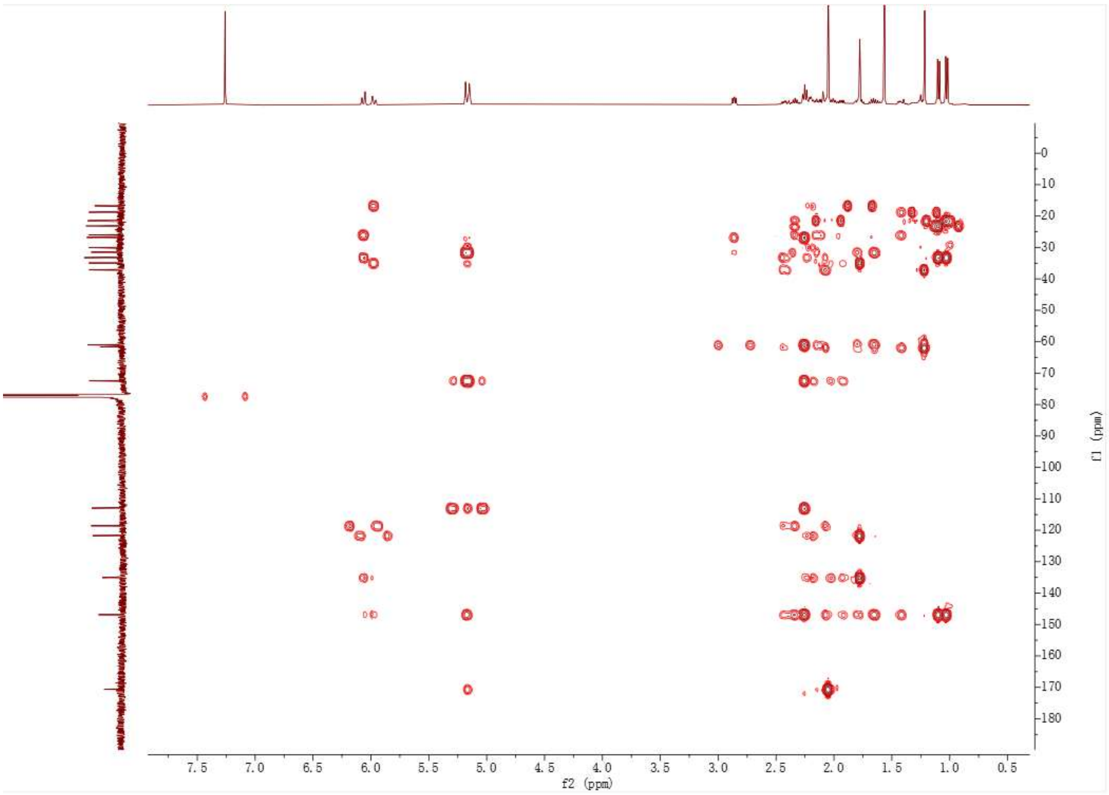

Figure S1.4. HMBC spectrum (600 MHz) of compound **1** in $CDCl_3$.

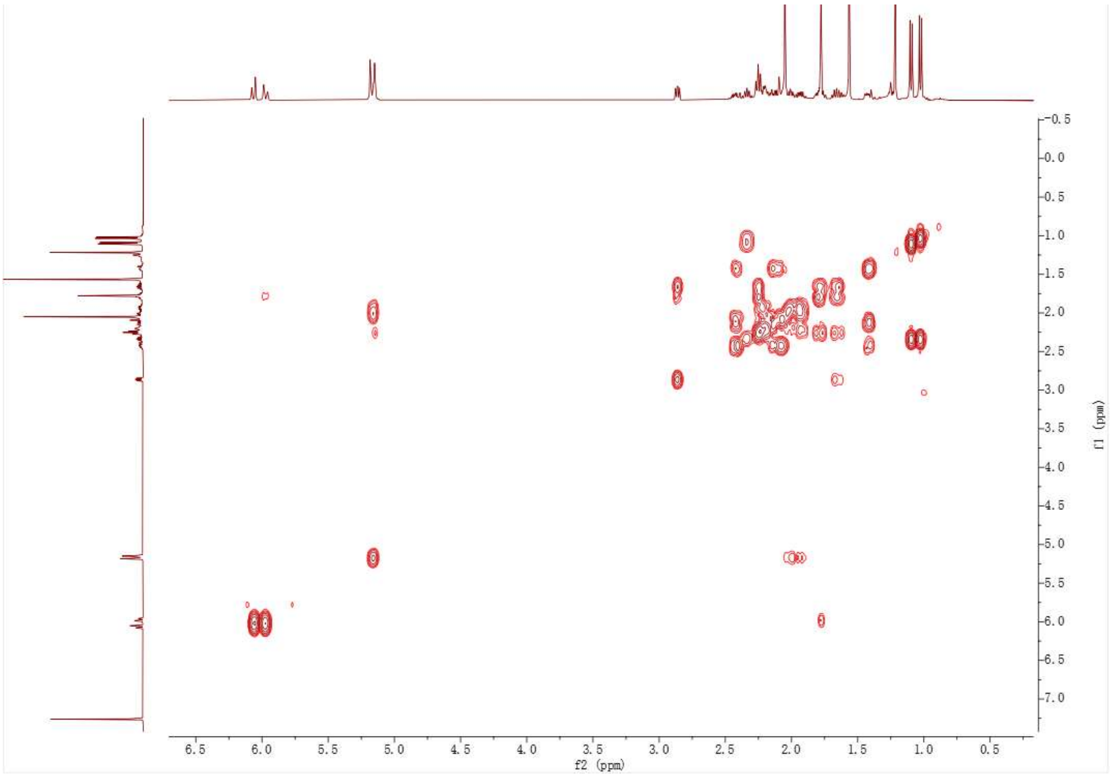

Figure S1.5. ^1H-^1H COSY spectrum (600 MHz) of compound **1** in $CDCl_3$.

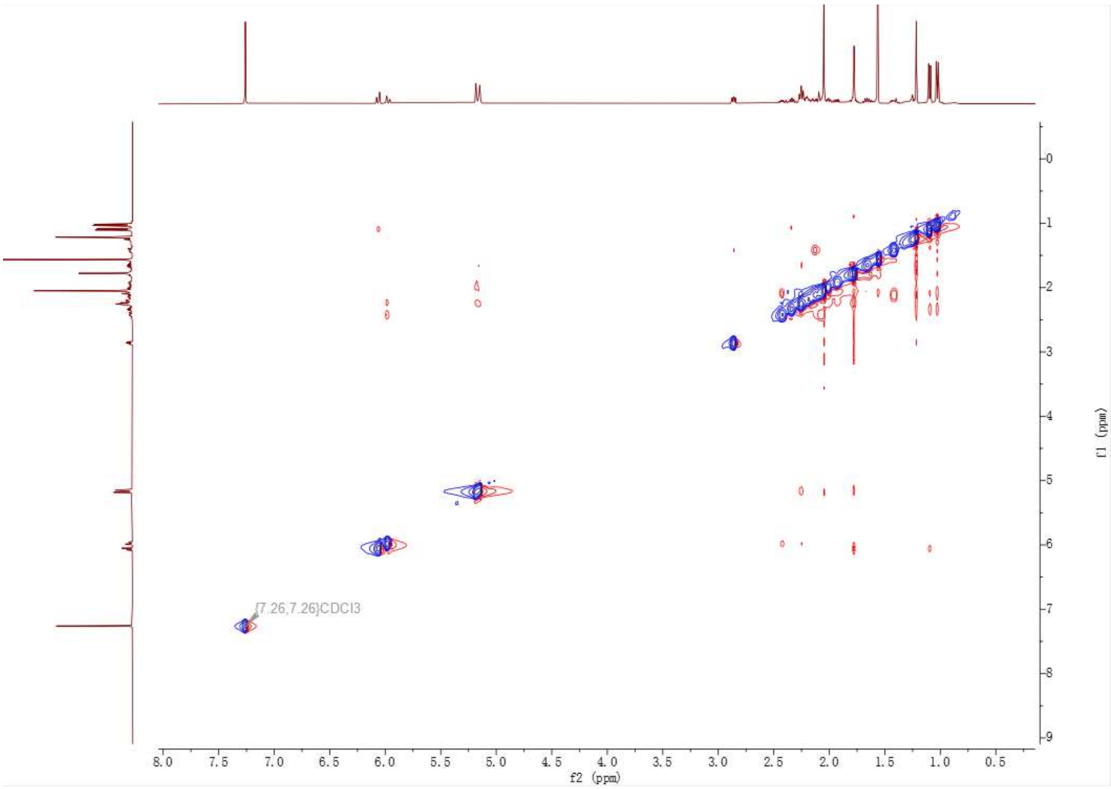

Figure S1.6. NOESY spectrum (600 MHz) of compound **1** in $CDCl_3$.

**Data Filename**	ESIH202203897.d	**Sample Name**	A8-Y2C13-3B
**Sample ID**		**Position**	P1-A8
**Instrument Name**	Agilent G6520 Q-TOF	**Acq Method**	20160322_MS_ESIH_POS_1min.m
**Acquired Time**	10/17/2022 13:26:41	**IRM Calibration Status**	Success
**DA Method**	small molecular data analysis method.m	**Comment**	ESIH by fangsu

User Spectra

Fragmentor Voltage	Collision Energy	Ionization Mode
170	0	ESI

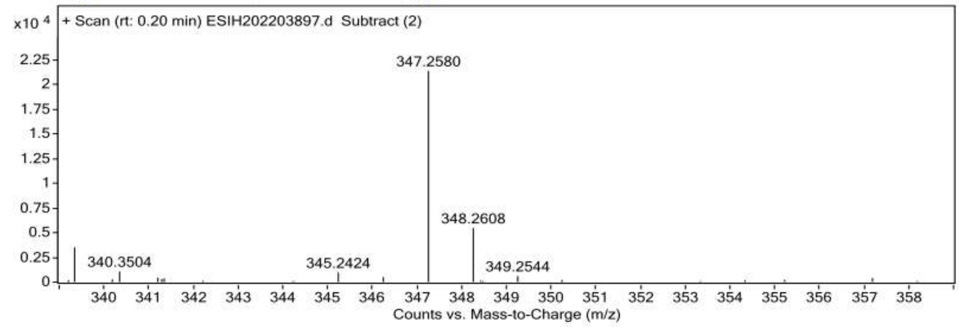

Formula Calculator Results

m/z	Calc m/z	Diff (mDa)	Diff (ppm)	Ion Formula	Ion
347.258	347.2581	0.04	0.12	C22 H35 O3	(M+H)+

Figure S1.7. HR-ESIMS spectrum of compound **1**.

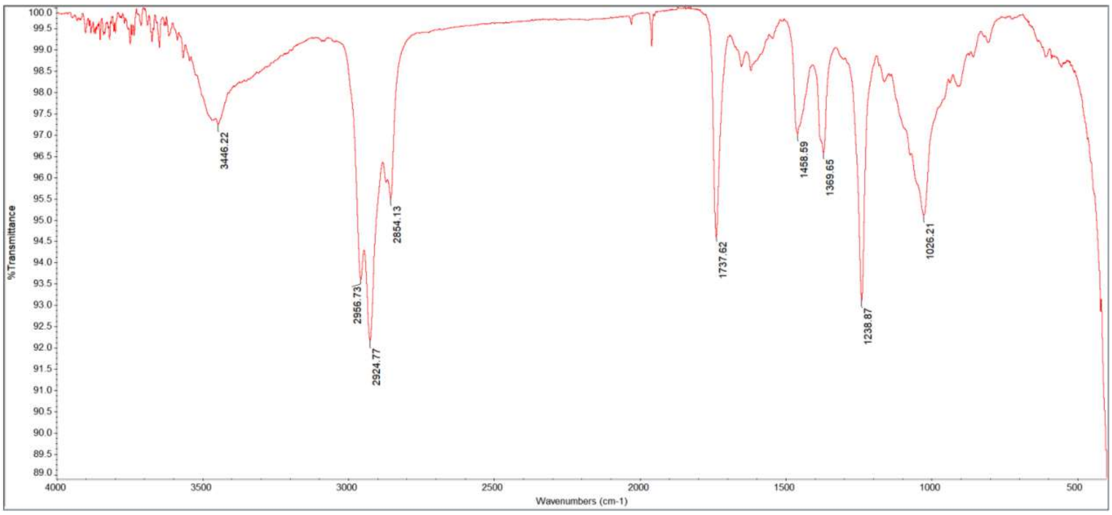

Figure S1.8. IR spectrum of compound **1**.

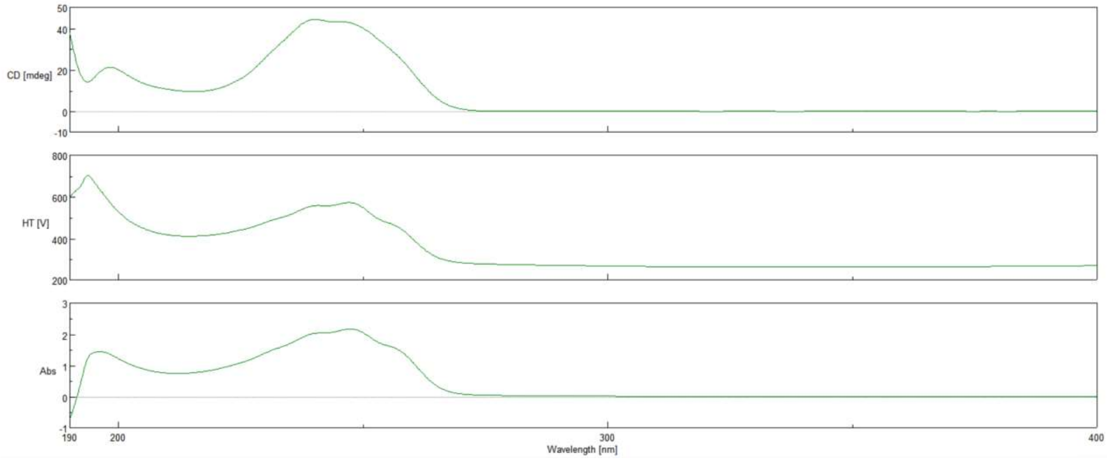

Figure S1.9. CD and UV spectra of compound **1**.

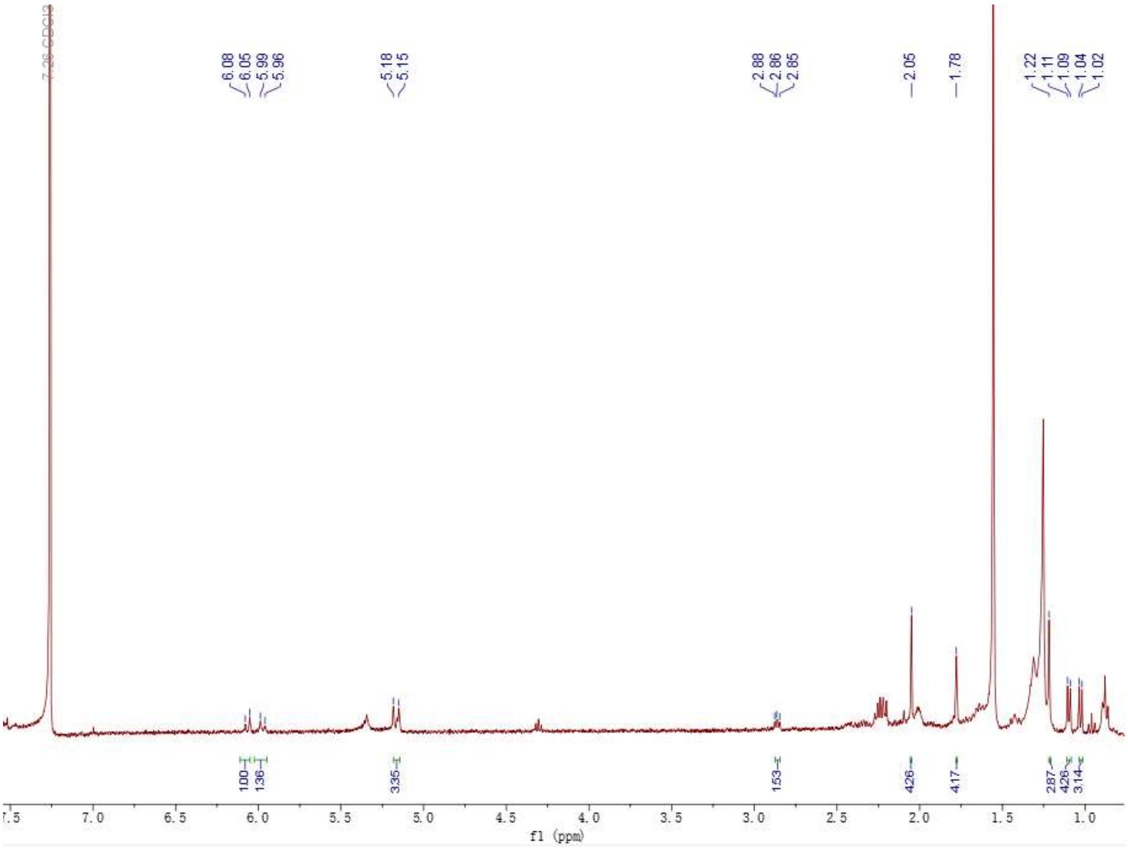

Figure S1.10. ^{1}H NMR spectrum (400 MHz) of compound **1a** in $CDCl_3$.

3. Spectra of compound 2

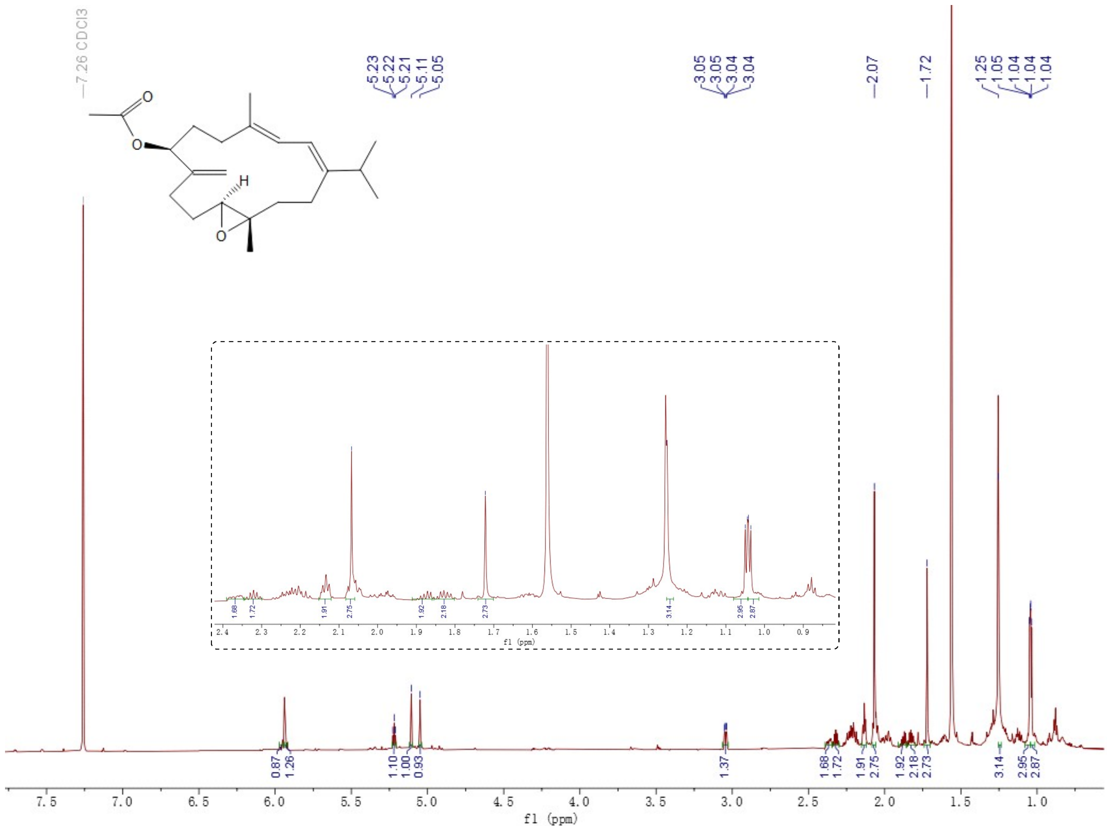

Figure S2.1. ^{1}H NMR spectrum (800 MHz) of compound **2** in $CDCl_3$.

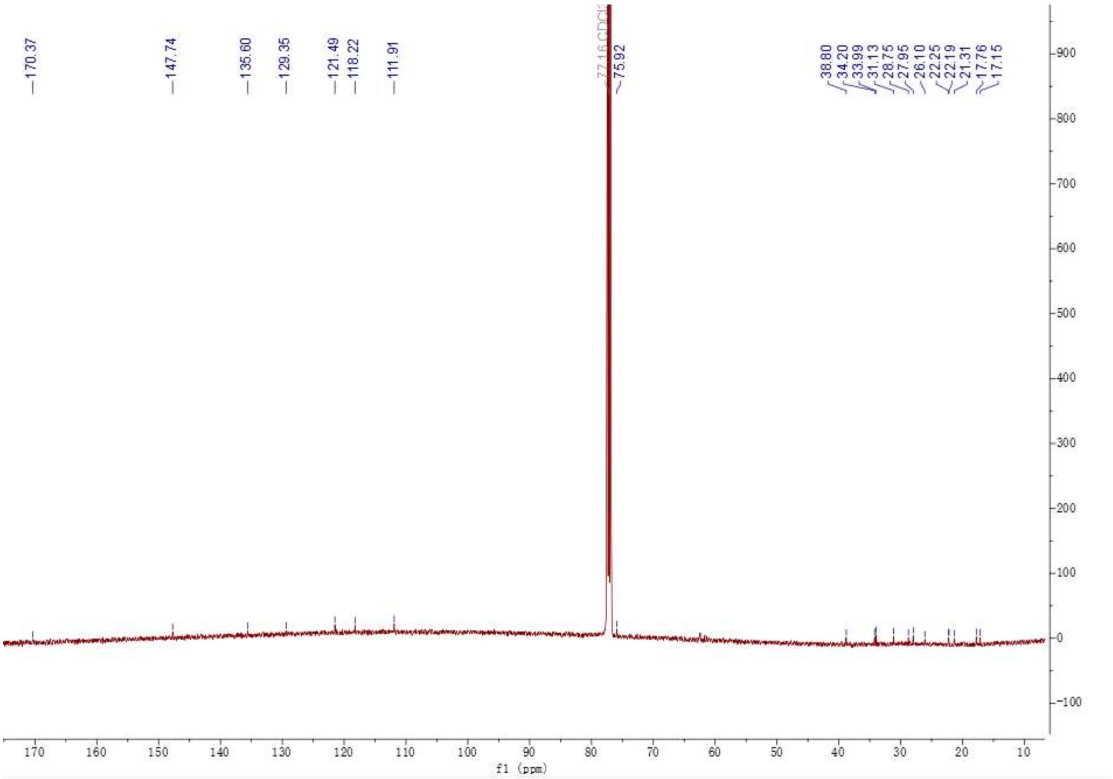

Figure S2.2. ^{13}C NMR spectrum (150 MHz) of compound **2** in $CDCl_3$.

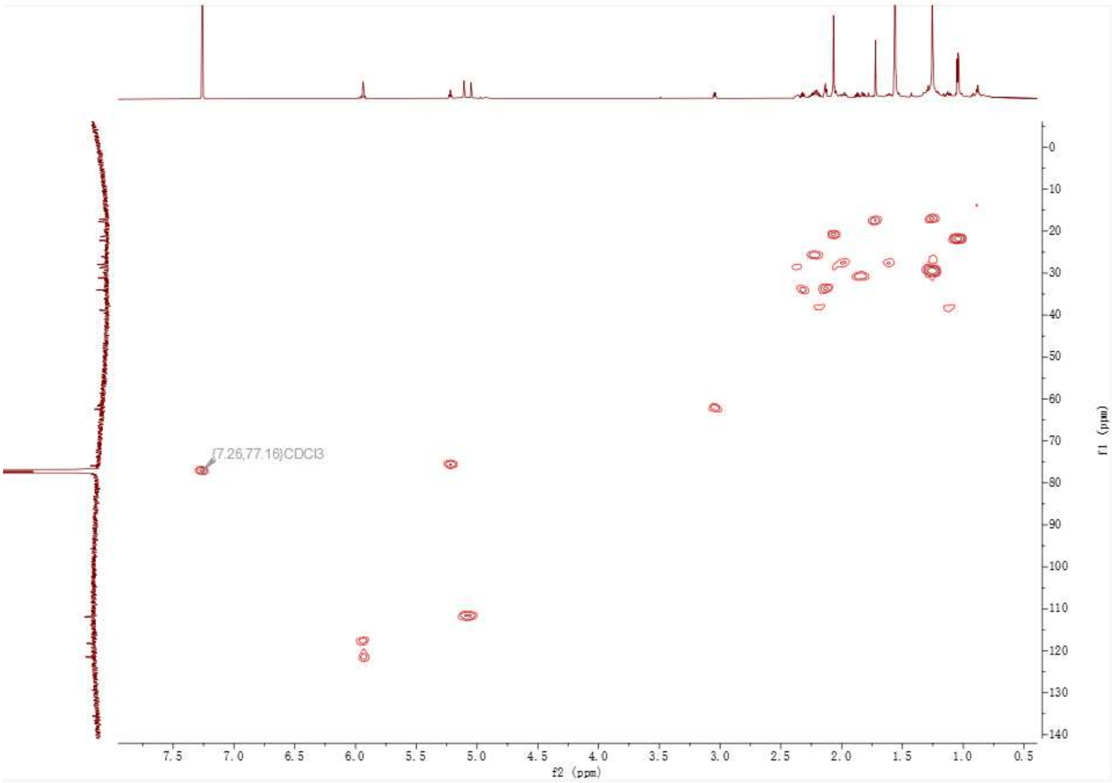

Figure S2.3. HSQC spectrum (800 MHz) of compound **2** in $CDCl_3$.

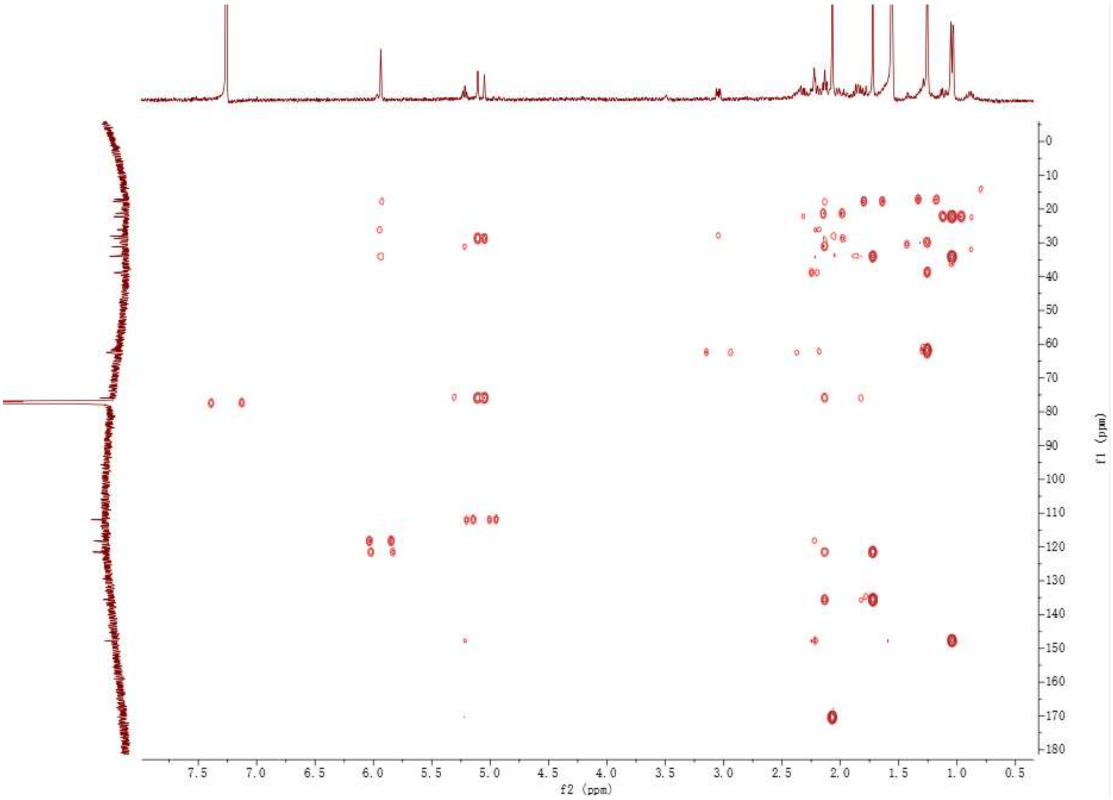

Figure S2.4. HMBC spectrum (800 MHz) of compound **2** in $CDCl_3$.

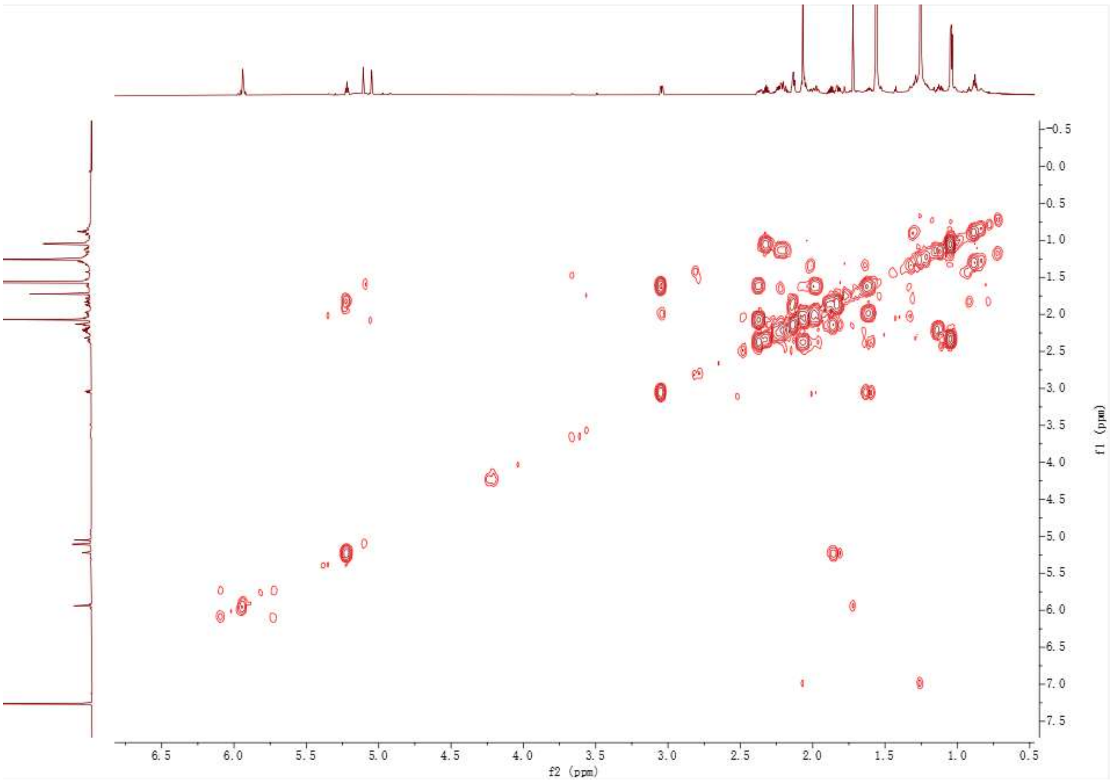

Figure S2.5. ^{1}H-^{1}H COSY spectrum (800 MHz) of compound **2** in $CDCl_3$.

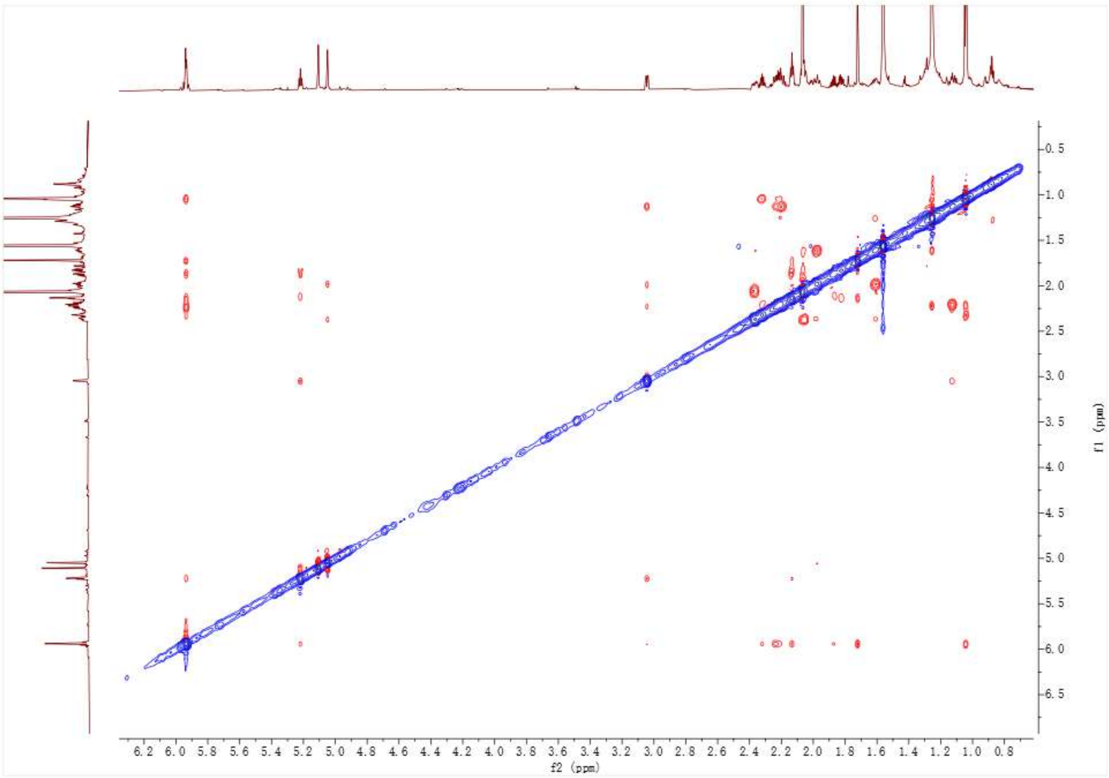

Figure S2.6. NOESY spectrum (800 MHz) of compound **2** in $CDCl_3$.

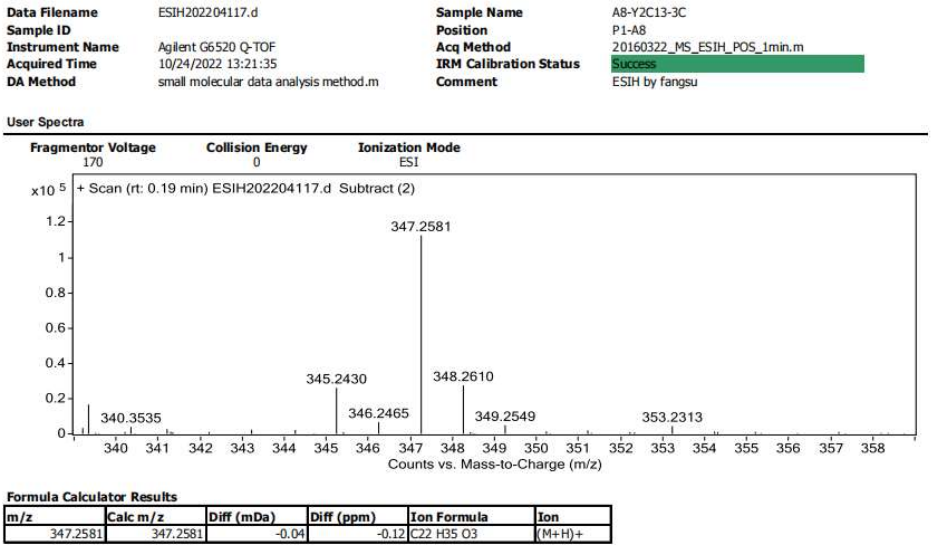

m/z	Calc m/z	Diff (mDa)	Diff (ppm)	Ion Formula	Ion
347.2581	347.2581	-0.04	-0.12	C22 H35 O3	(M+H)+

Figure S2.7. HR-ESIMS spectrum of compound **2**.

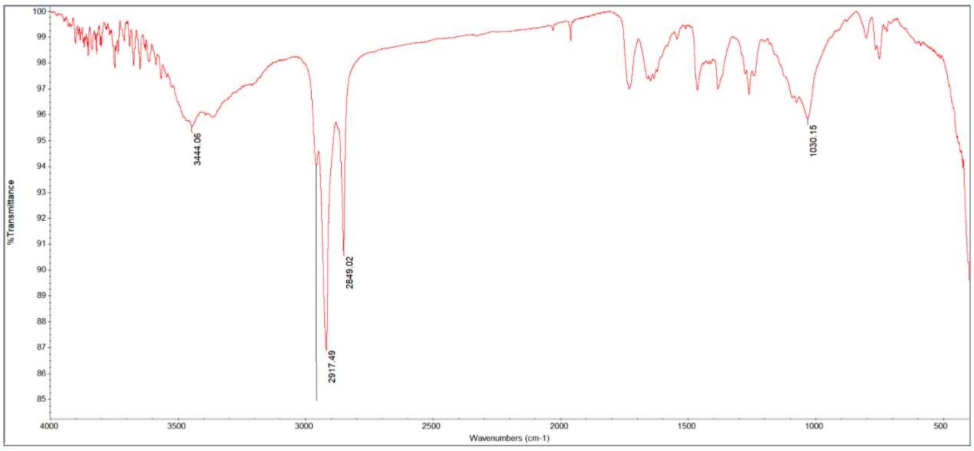

Figure S2.8. IR spectrum of compound **2**.

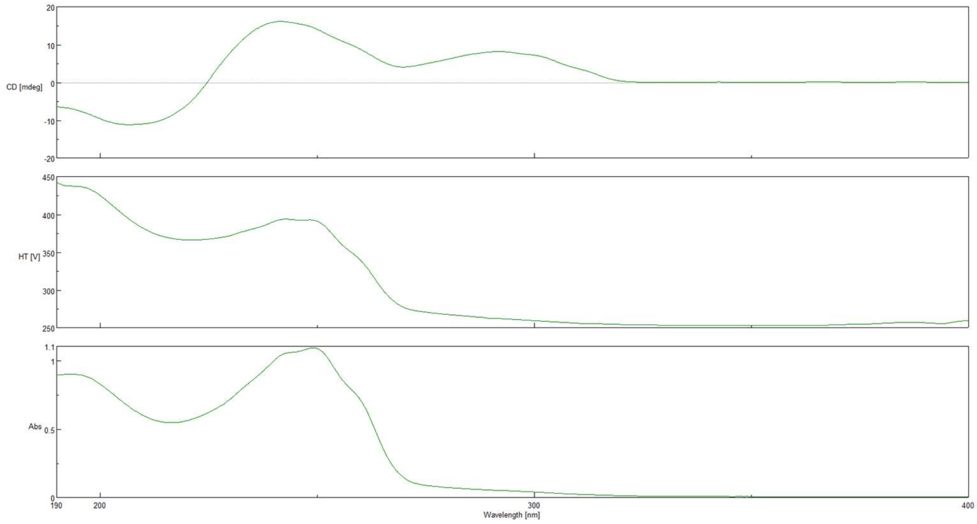

Figure S2.9. CD and UV spectra of compound **2**.

4. Spectra of compound 3

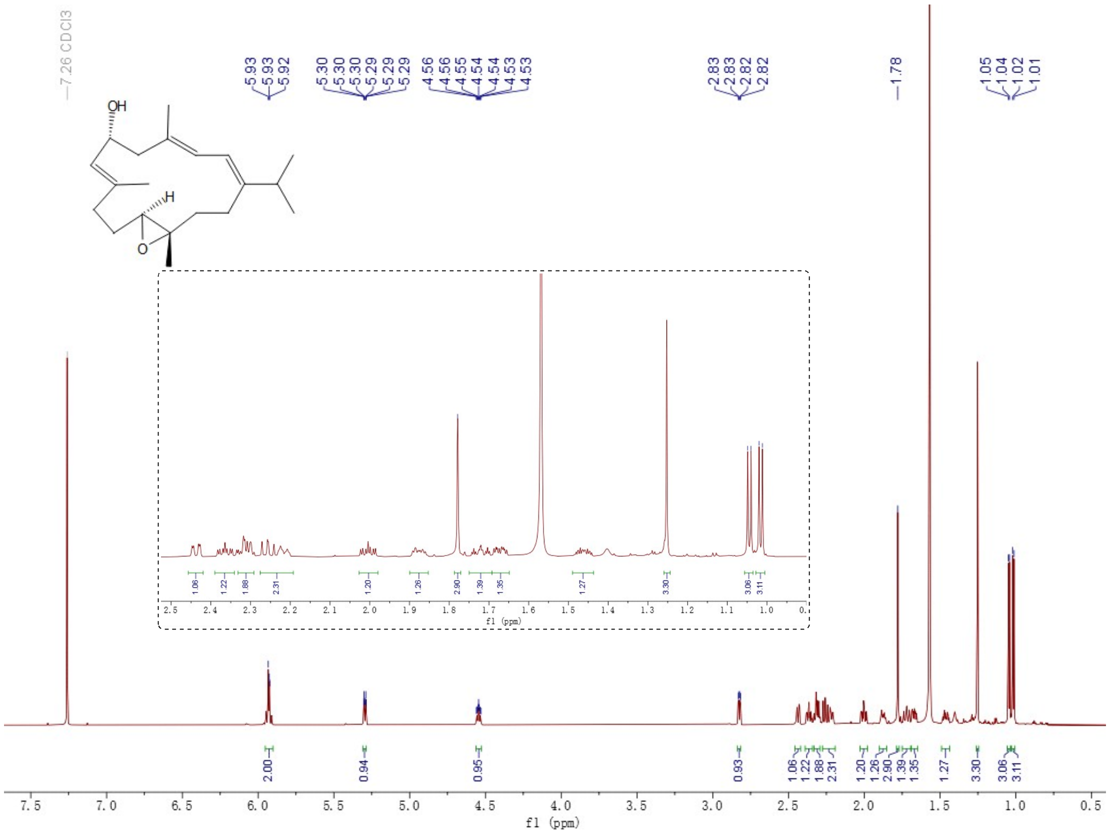

Figure S3.1. ^{1}H NMR spectrum (800 MHz) of compound **3** in $CDCl_3$.

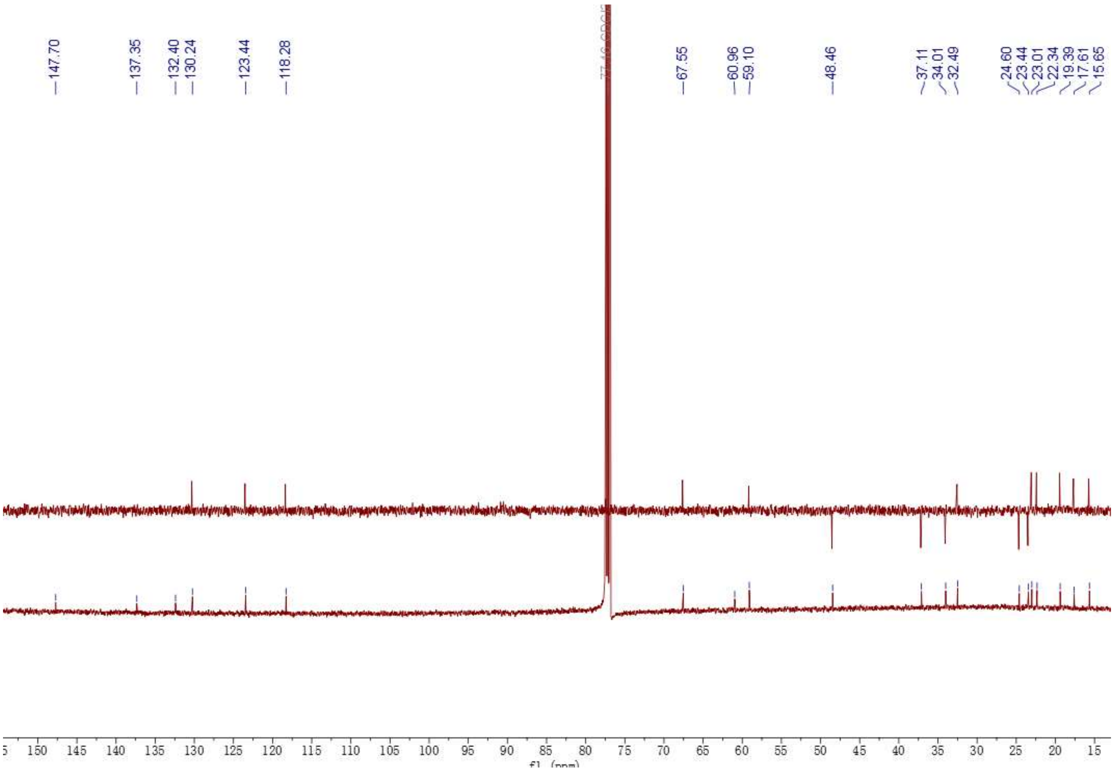

Figure S3.2. ^{13}C NMR spectrum (150 MHz) of compound **3** in $CDCl_3$.

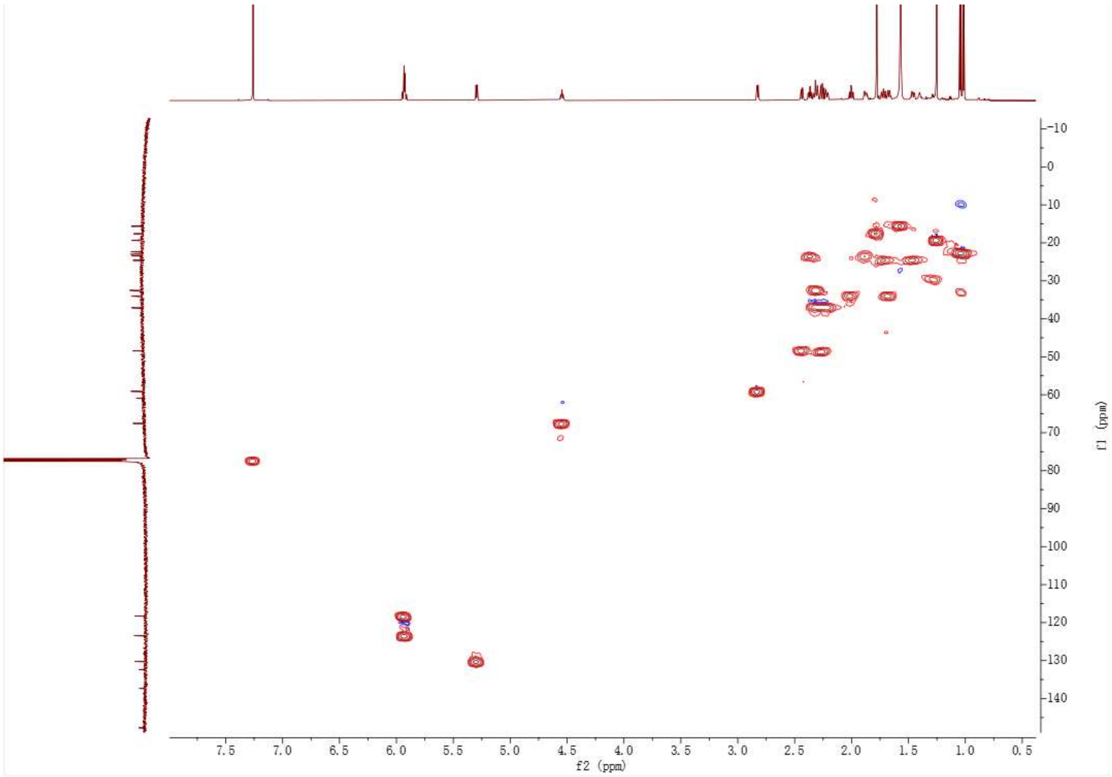

Figure S3.3. HSQC spectrum (800 MHz) of compound **3** in $CDCl_3$.

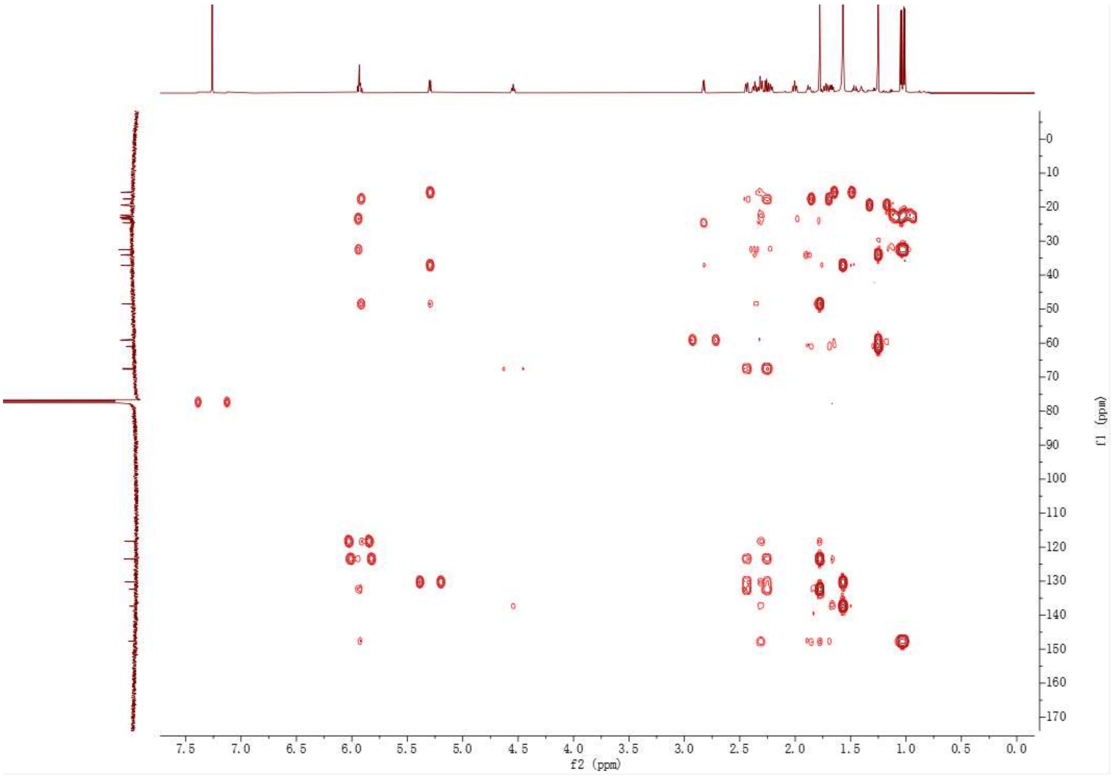

Figure S3.4. HMBC spectrum (800 MHz) of compound **3** in $CDCl_3$.

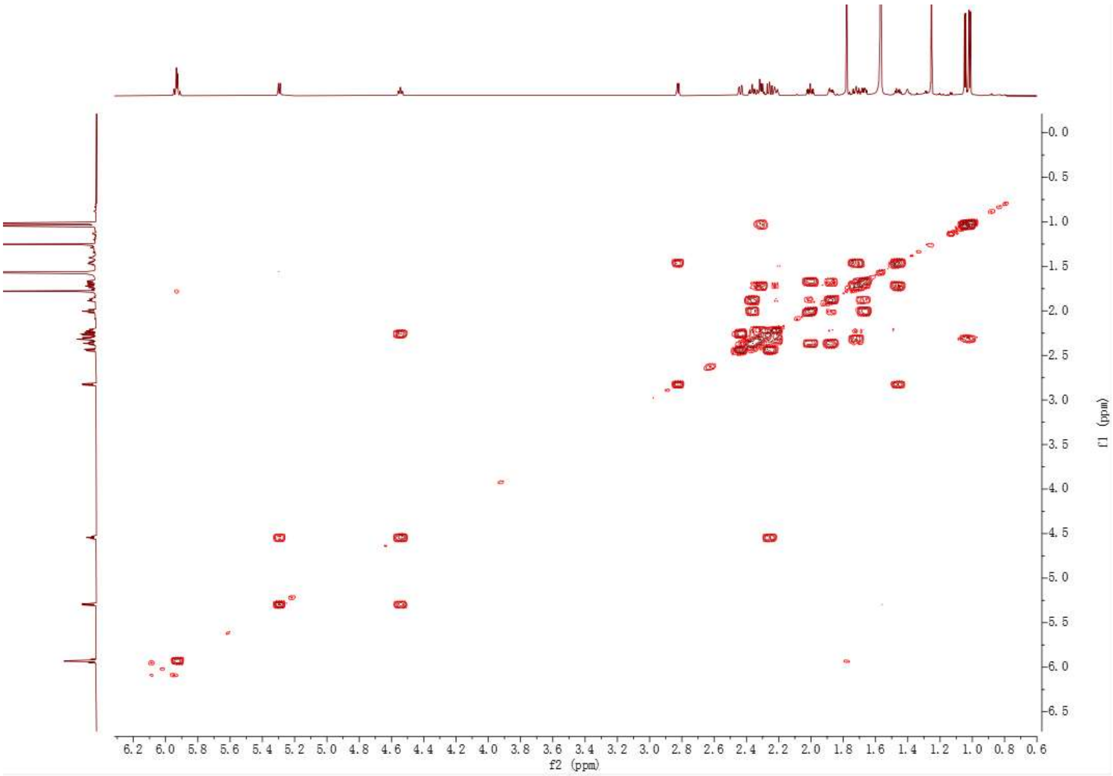

Figure S3.5. ^{1}H-^{1}H COSY spectrum (800 MHz) of compound **3** in $CDCl_3$.

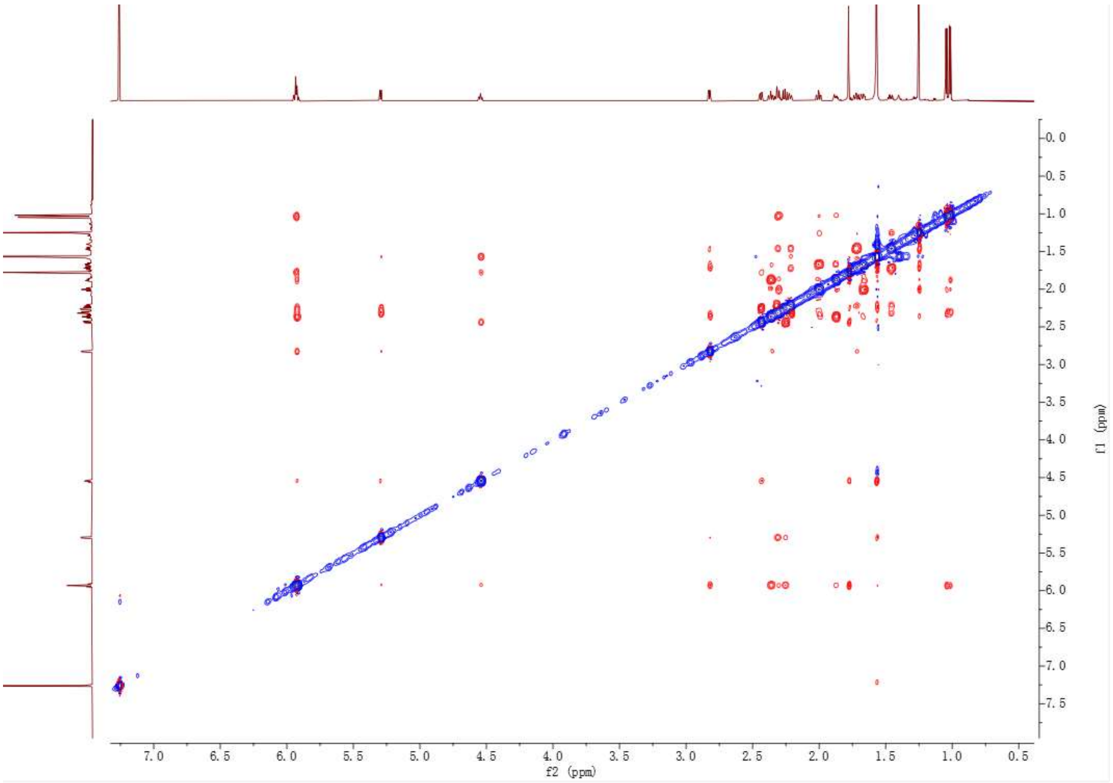

Figure S3.6. NOESY spectrum (800 MHz) of compound **3** in $CDCl_3$.

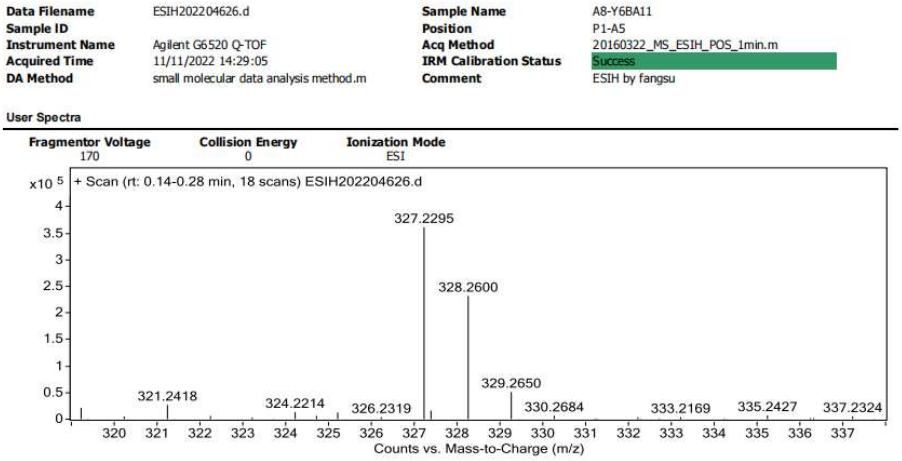

Formula Calculator Results

m/z	Calc m/z	Diff (mDa)	Diff (ppm)	Ion Formula	Ion
327.2295	327.2295	-0.04	-0.13	C20 H32 Na O2	(M+Na)+

Figure S3.7. HR-ESIMS spectrum of compound **3**.

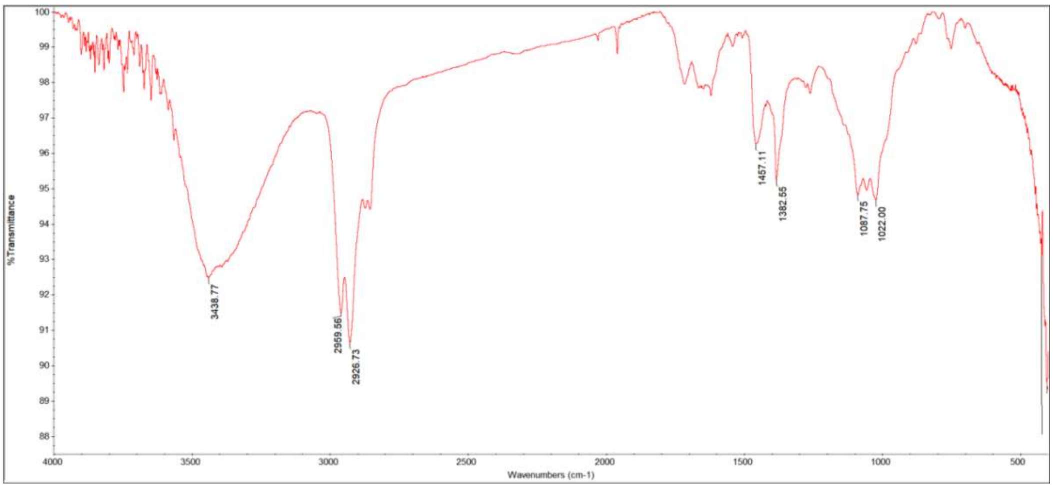

Figure S3.8. IR spectrum of compound **3**.

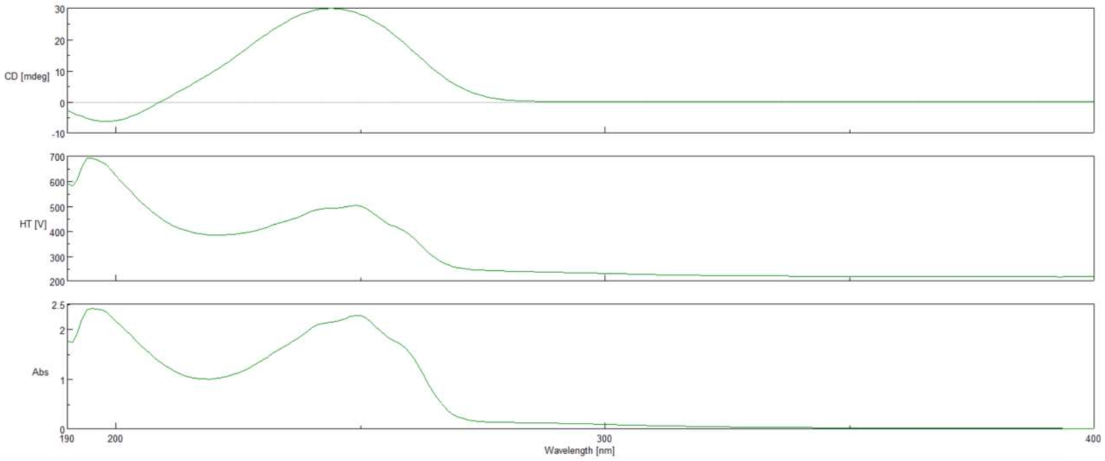

Figure S3.9. CD and UV spectrum of compound **3**.

5. Spectra of compound 4

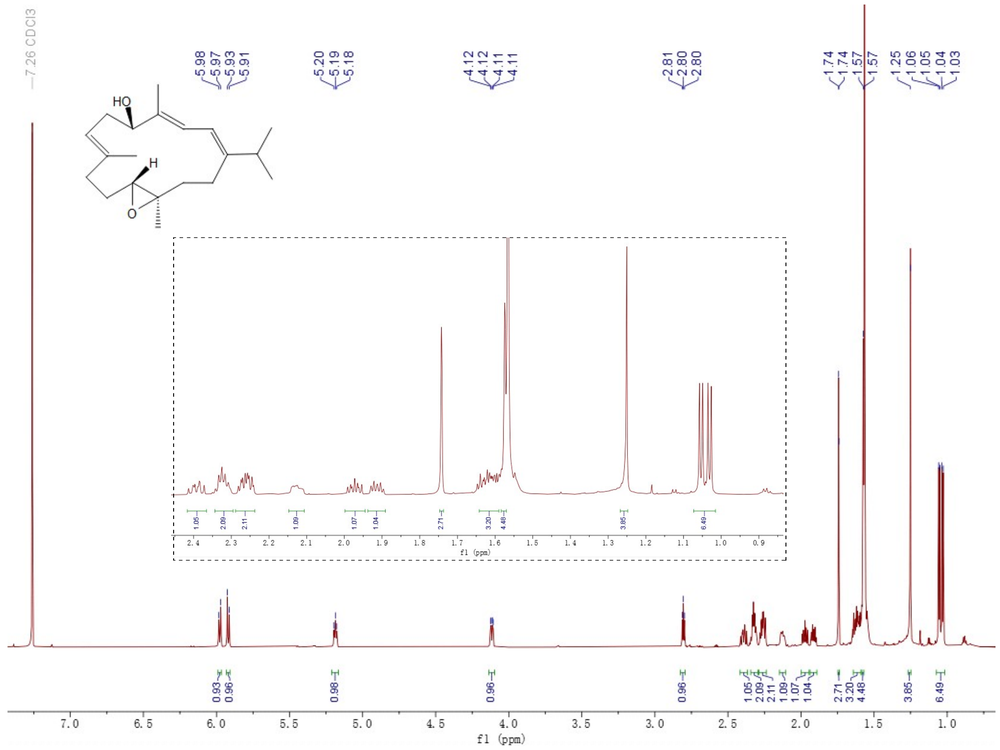

Figure S4.1. ^{1}H NMR spectrum (800 MHz) of compound **4** in $CDCl_3$.

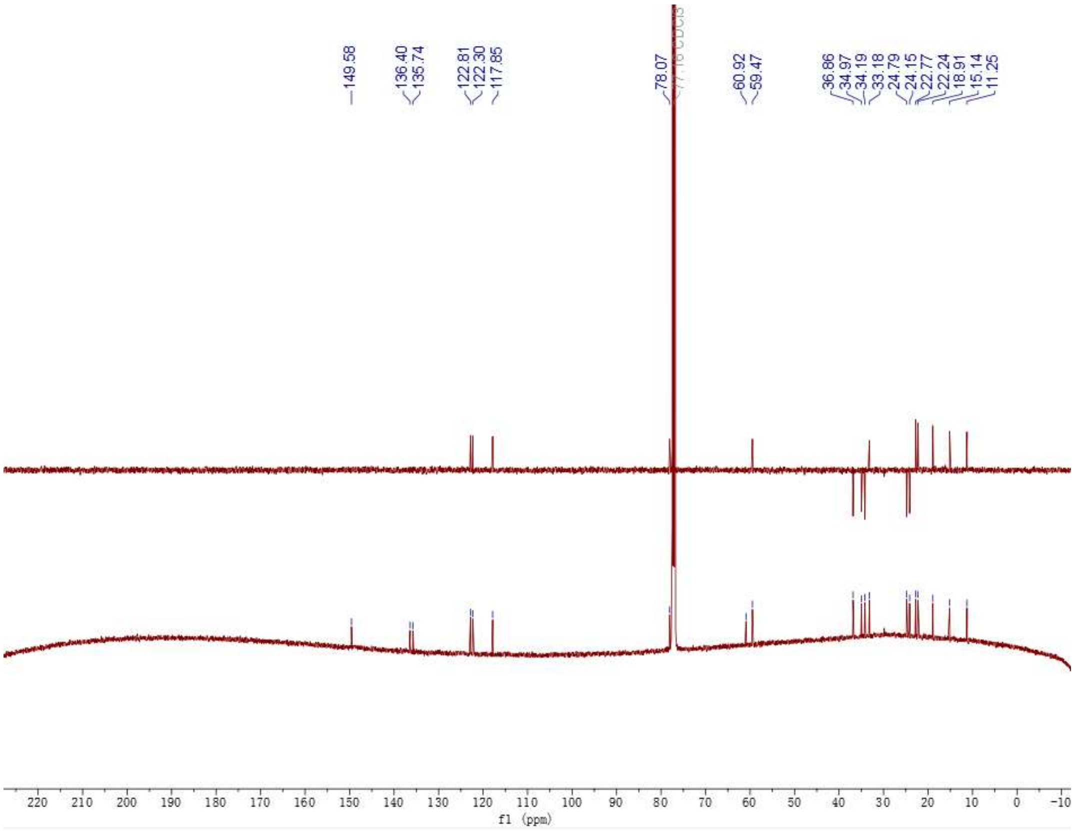

Figure S4.2. ^{13}C NMR spectrum (150 MHz) of compound **4** in $CDCl_3$.

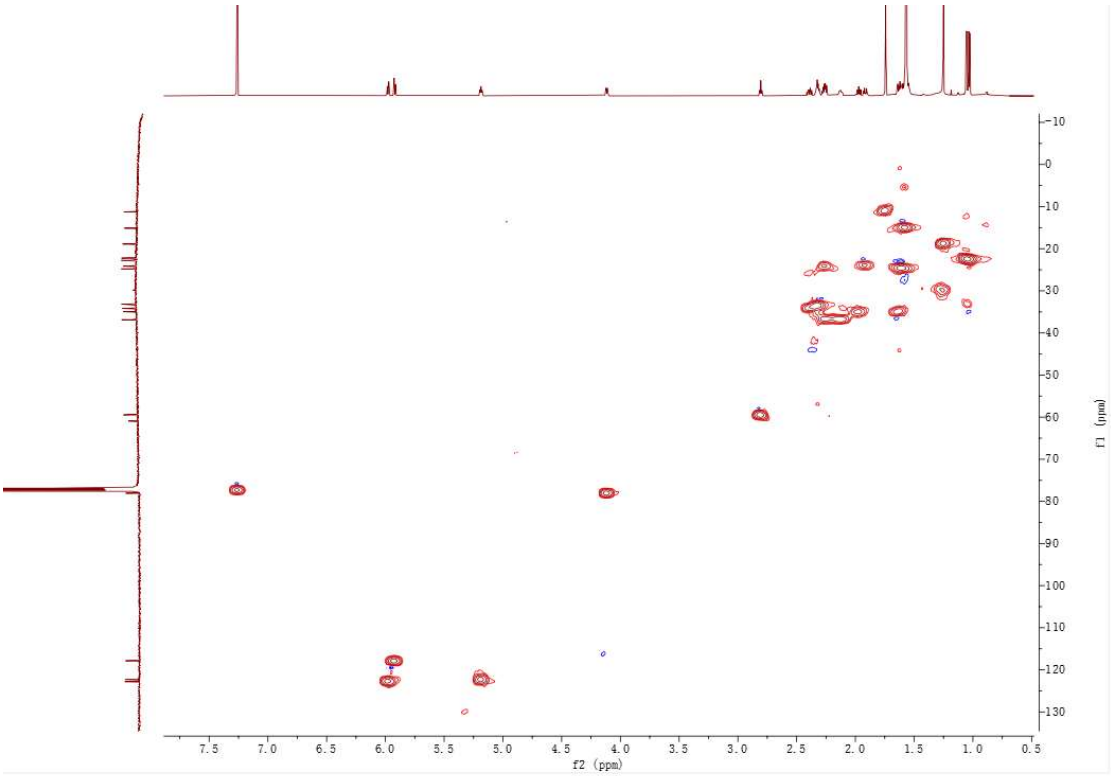

Figure S4.3. HSQC spectrum (800 MHz) of compound **4** in $CDCl_3$.

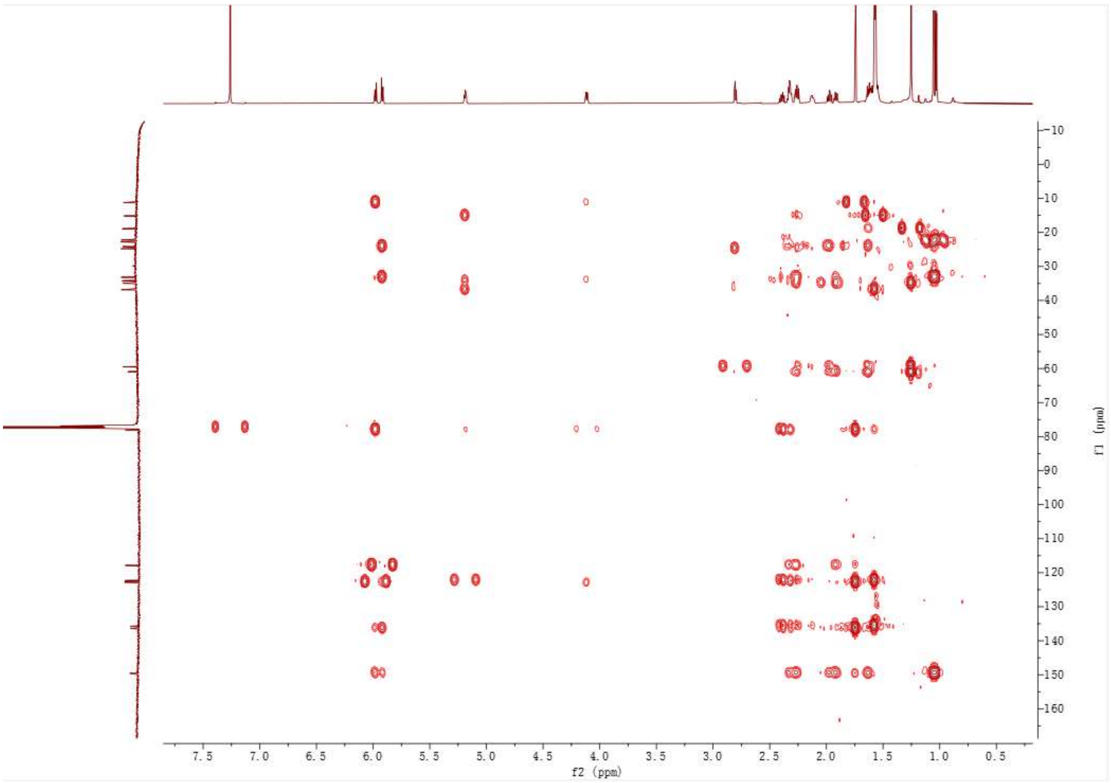

Figure S4.4. HMBC spectrum (800 MHz) of compound **4** in $CDCl_3$.

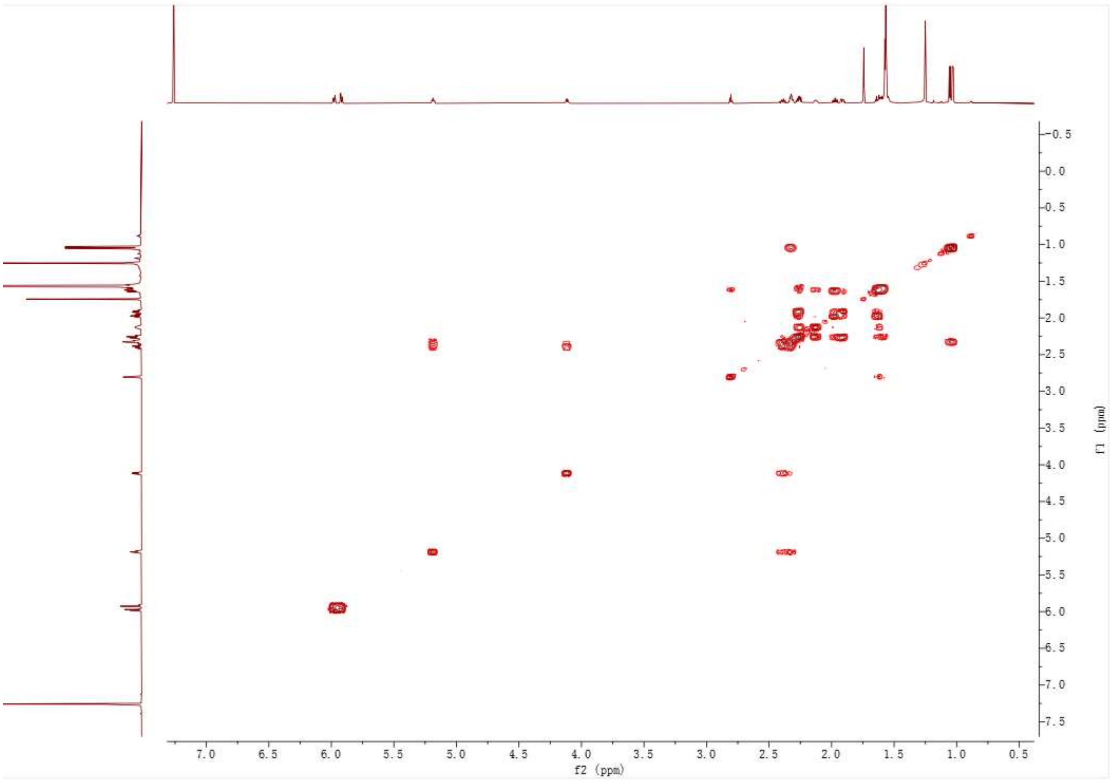

Figure S4.5. ^{1}H-^{1}H COSY spectrum (800 MHz) of compound **4** in $CDCl_3$.

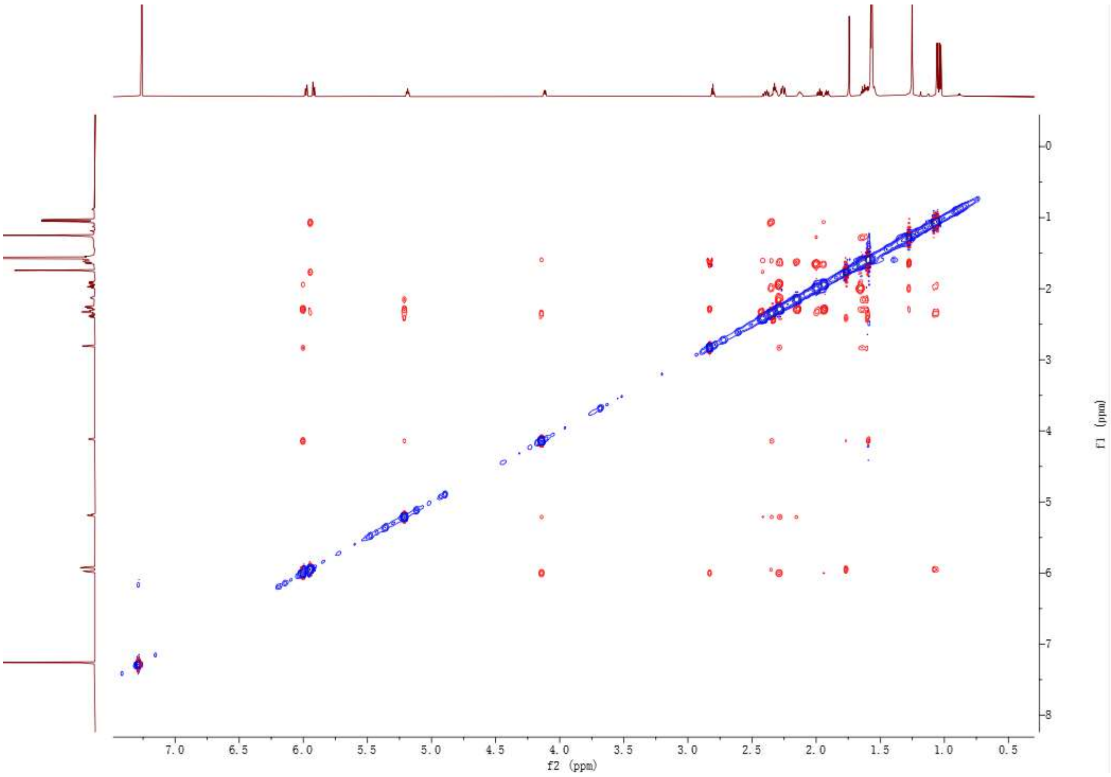

Figure S4.6. NOESY spectrum (800 MHz) of compound **4** in $CDCl_3$.

**Data Filename**	ESIH202204627.d	**Sample Name**	A8-Y6BA12
**Sample ID**		**Position**	P1-A6
**Instrument Name**	Agilent G6520 Q-TOF	**Acq Method**	20160322_MS_ESIH_POS_1min.m
**Acquired Time**	11/11/2022 14:30:22	**IRM Calibration Status**	Success
**DA Method**	small molecular data analysis method.m	**Comment**	ESIH by fangsu

User Spectra

Fragmentor Voltage	Collision Energy	Ionization Mode
170	0	ESI

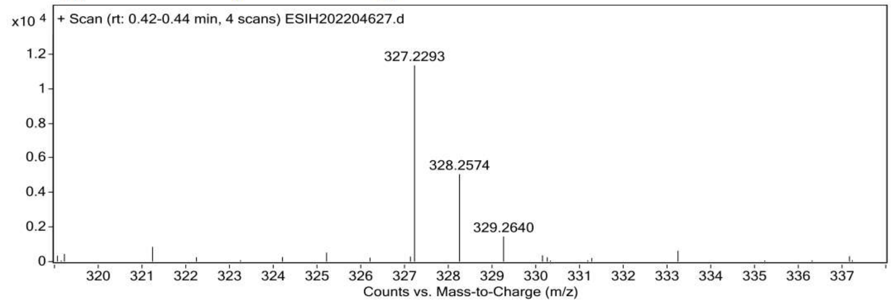

Formula Calculator Results

m/z	Calc m/z	Diff (mDa)	Diff (ppm)	Ion Formula	Ion
327.2293	327.2295	0.15	0.45	C20 H32 Na O2	(M+Na)+

Figure S4.7. HR-ESIMS spectrum of compound **4**.

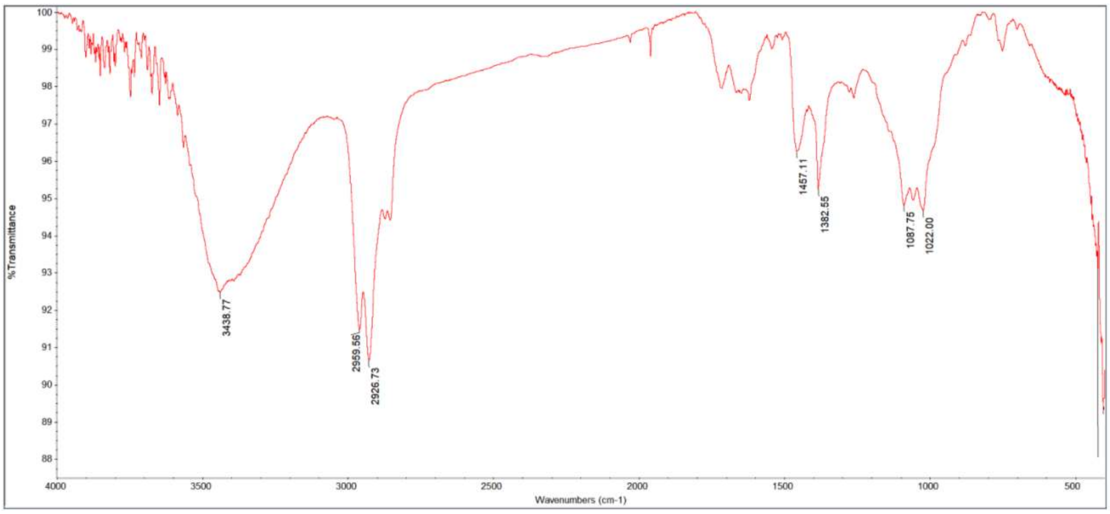

Figure S4.8. IR spectrum of compound **4**.

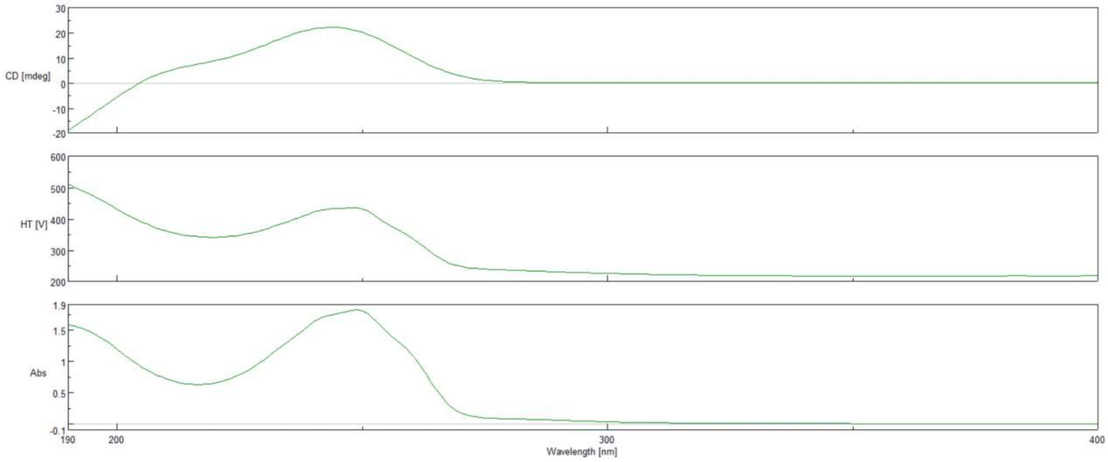

Figure S4.9. CD and UV spectrum of compound **4**.

6. Spectra of compound 5

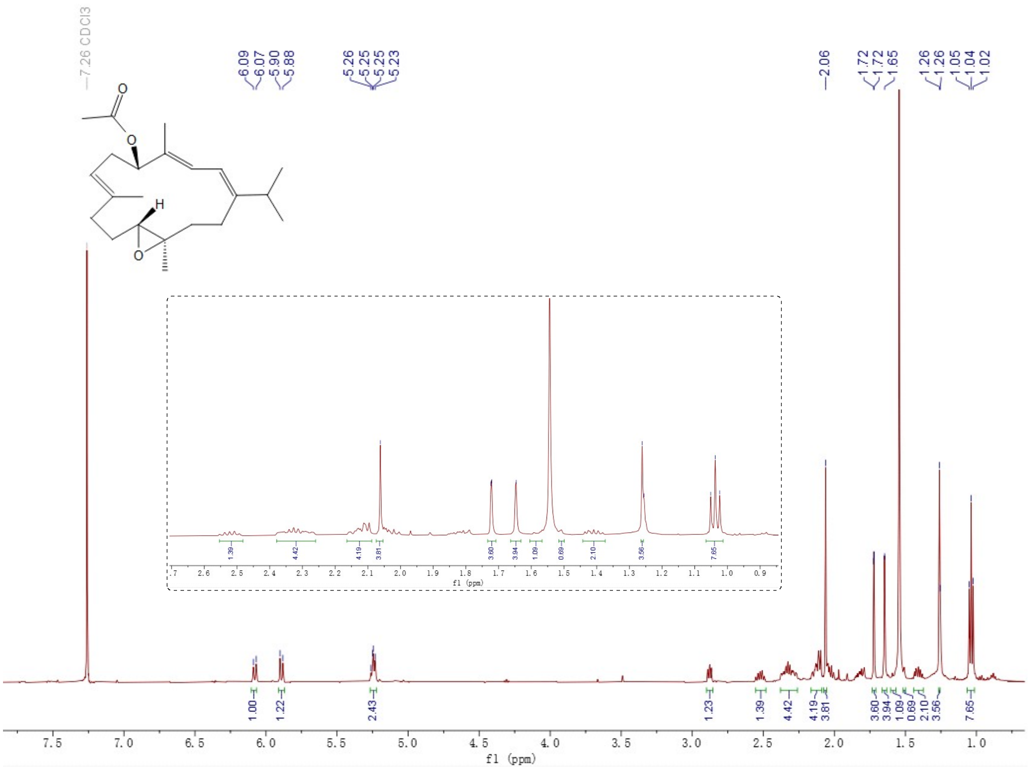

Figure S5.1. ^{1}H NMR spectrum (600 MHz) of compound **5** in $CDCl_3$.

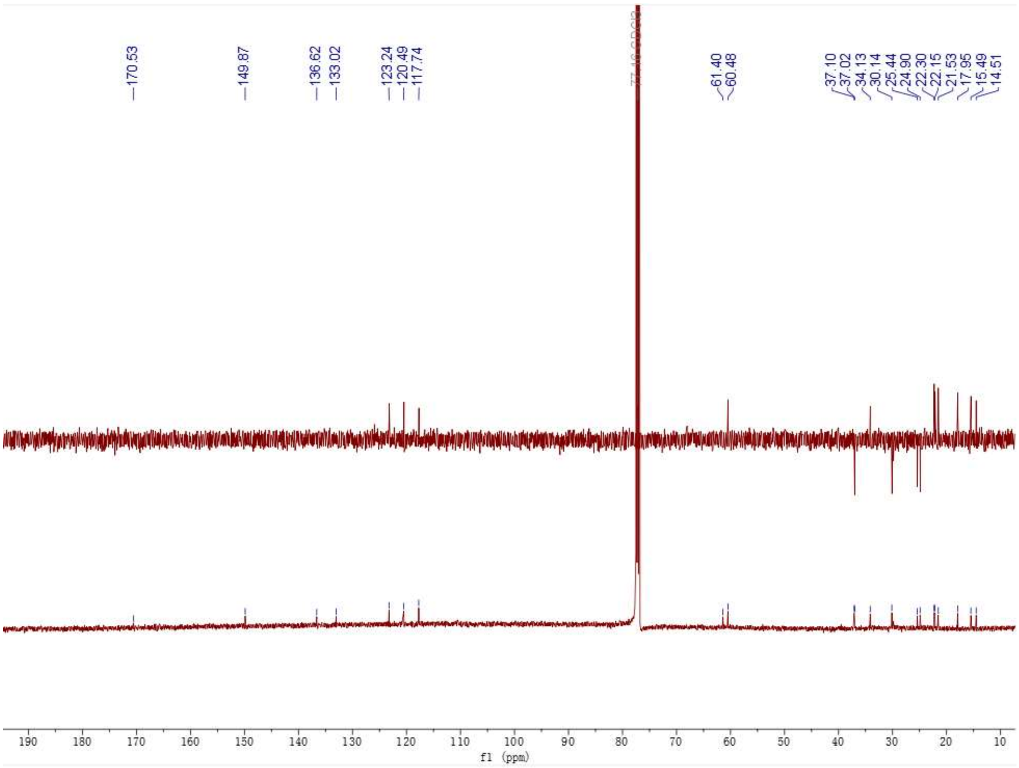

Figure S5.2. ^{13}C NMR spectrum (150 MHz) of compound **5** in $CDCl_3$.

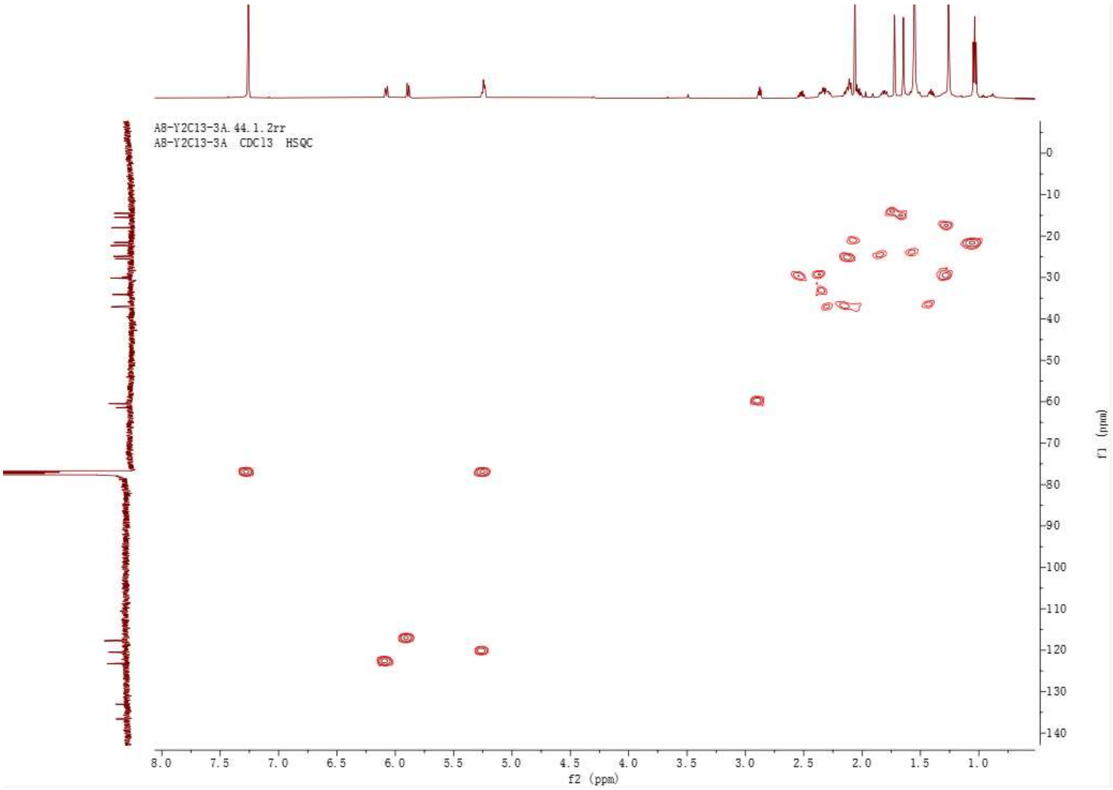

Figure S5.3. HSQC spectrum (600 MHz) of compound **5** in $CDCl_3$.

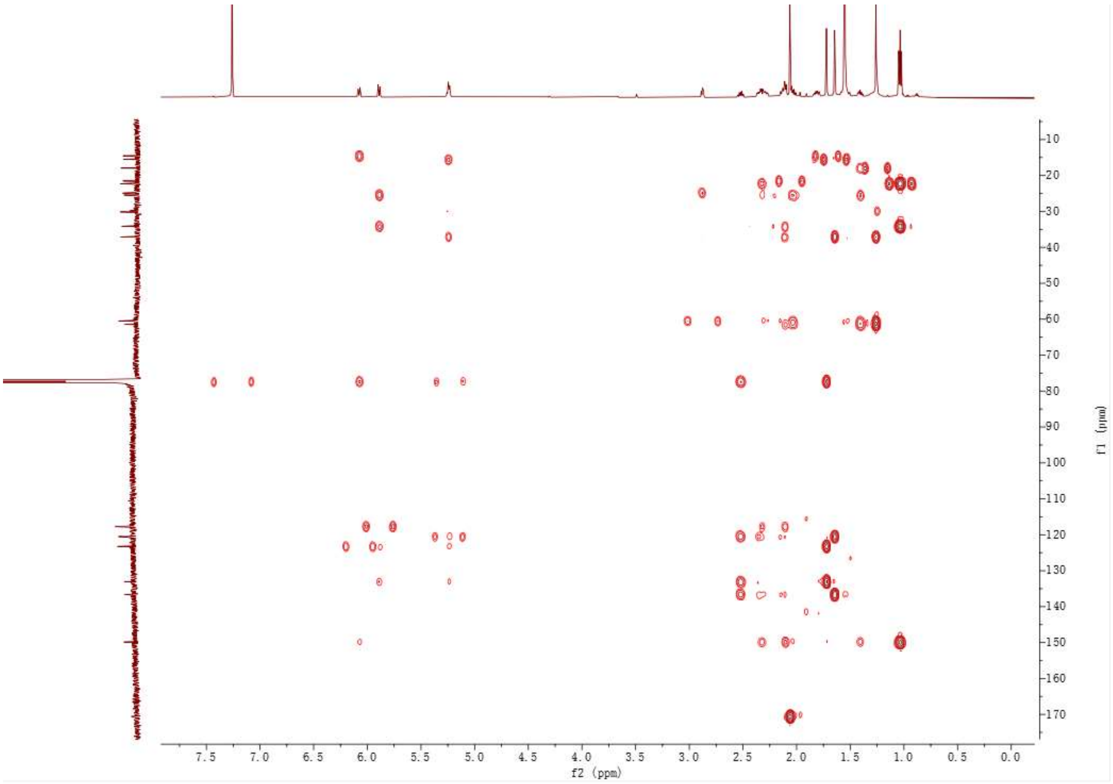

Figure S5.4. HMBC spectrum (600 MHz) of compound **5** in $CDCl_3$.

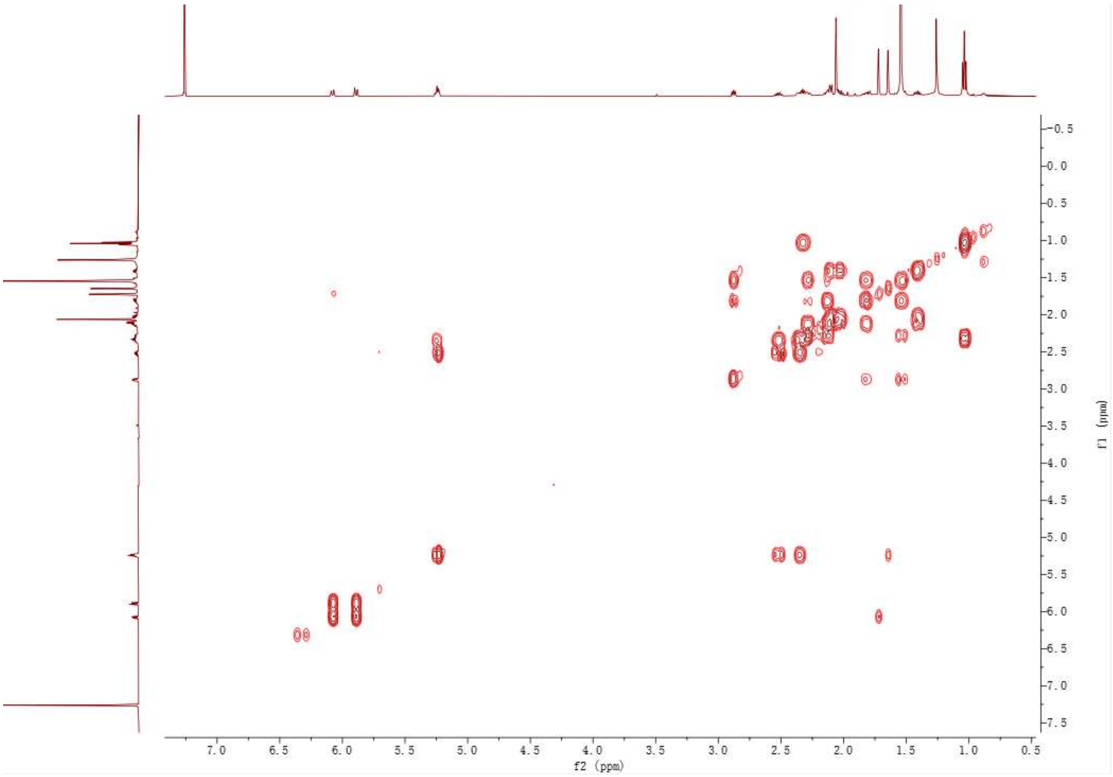

Figure S5.5. ^{1}H-^{1}H COSY spectrum (600 MHz) of compound **5** in $CDCl_3$.

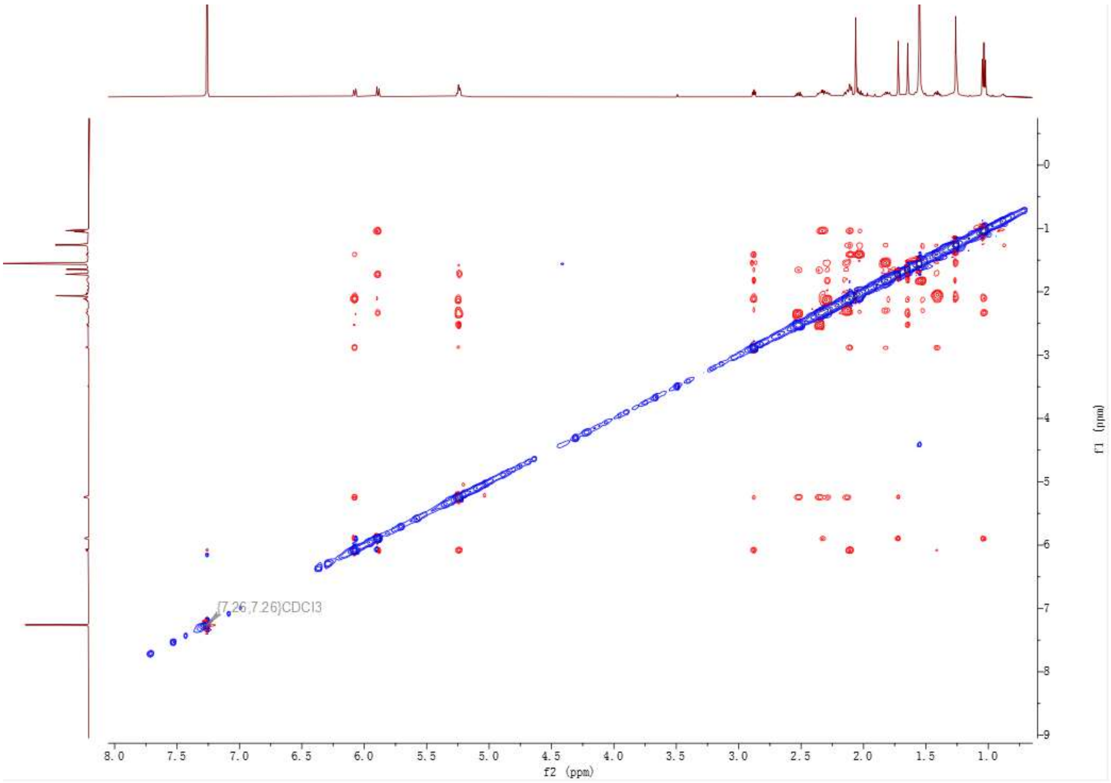

Figure S5.6. NOESY spectrum (600 MHz) of compound **5** in $CDCl_3$.

**Data Filename**	ESIH202203894.d	**Sample Name**	A8-Y2C13-3A
**Sample ID**		**Position**	P1-A5
**Instrument Name**	Agilent G6520 Q-TOF	**Acq Method**	20160322_MS_ESIH_POS_1min.m
**Acquired Time**	10/17/2022 13:22:49	**IRM Calibration Status**	Success
**DA Method**	small molecular data analysis method.m	**Comment**	ESIH by fangsu

User Spectra

**Fragmentor Voltage**	**Collision Energy**	**Ionization Mode**
170	0	ESI

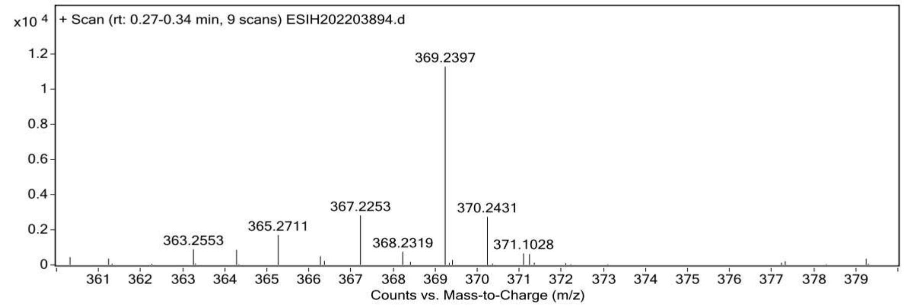

Formula Calculator Results

m/z	Calc m/z	Diff (mDa)	Diff (ppm)	Ion Formula	Ion
369.2397	369.24	0.36	0.98	C22 H34 Na O3	(M+Na)+

Figure S5.7. HR-ESIMS spectrum of compound **5**.

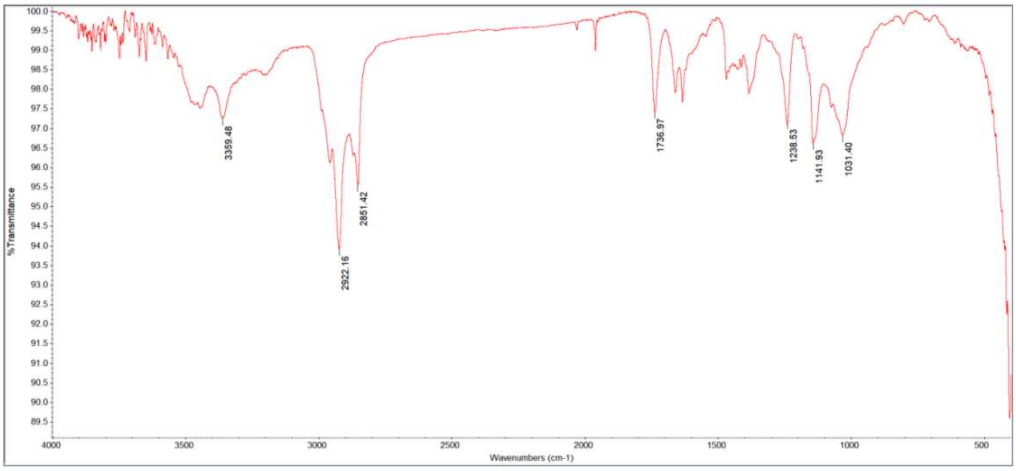

Figure S5.8. IR spectrum of compound **5**.

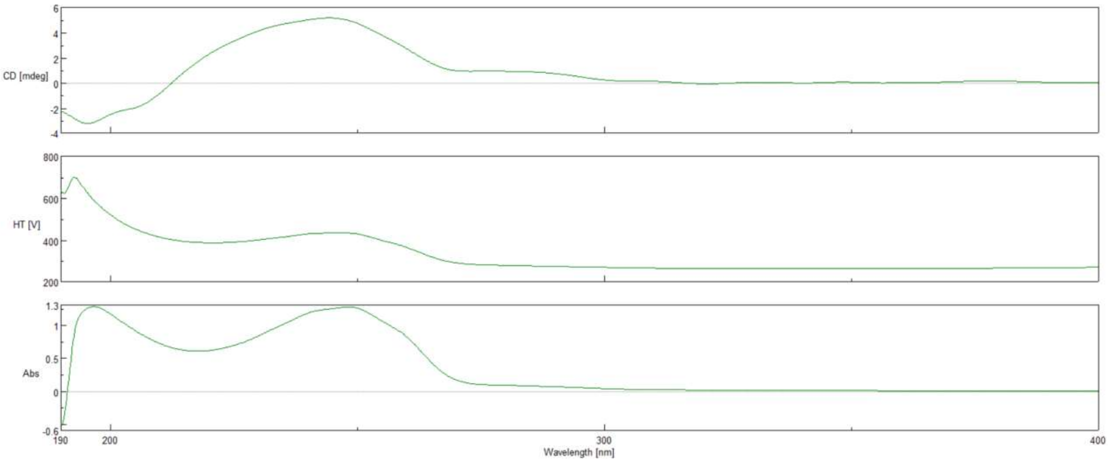

Figure S5.9. CD and UV spectrum of compound **5**.

7. Spectra of compound 9

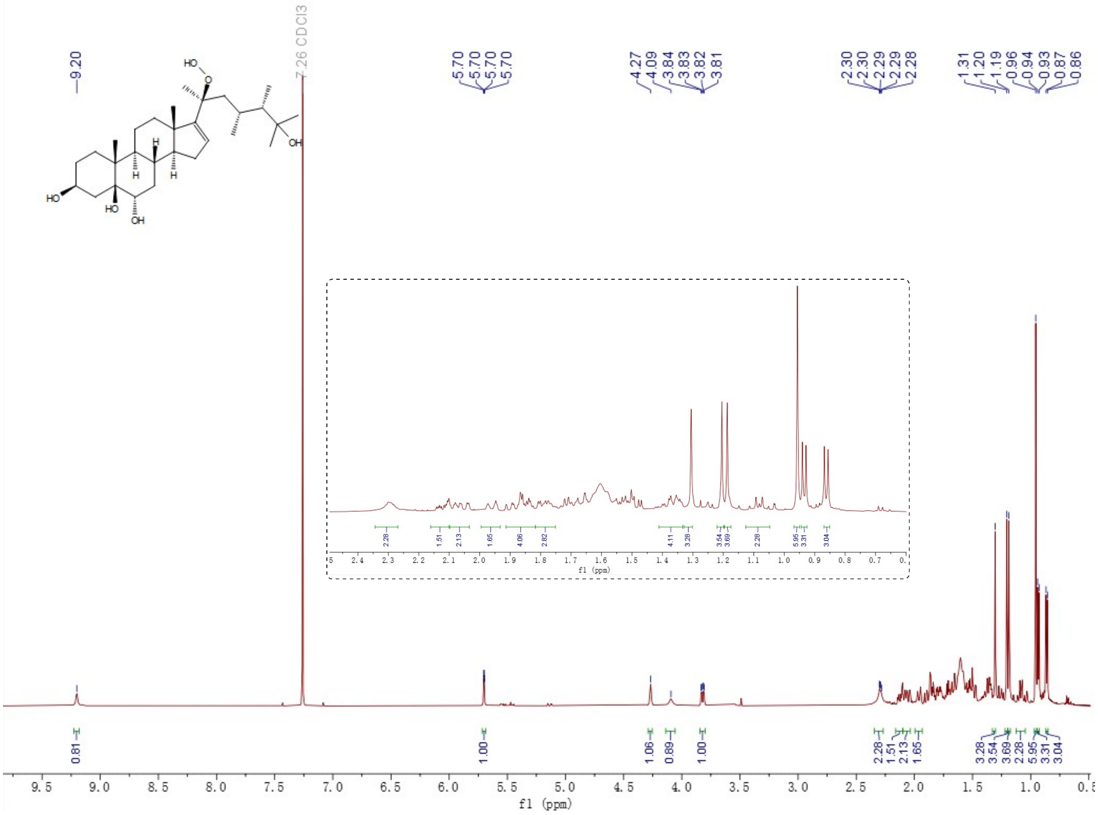

Figure S6.1. ^{1}H NMR spectrum (600 MHz) of compound **9** in $CDCl_3$.

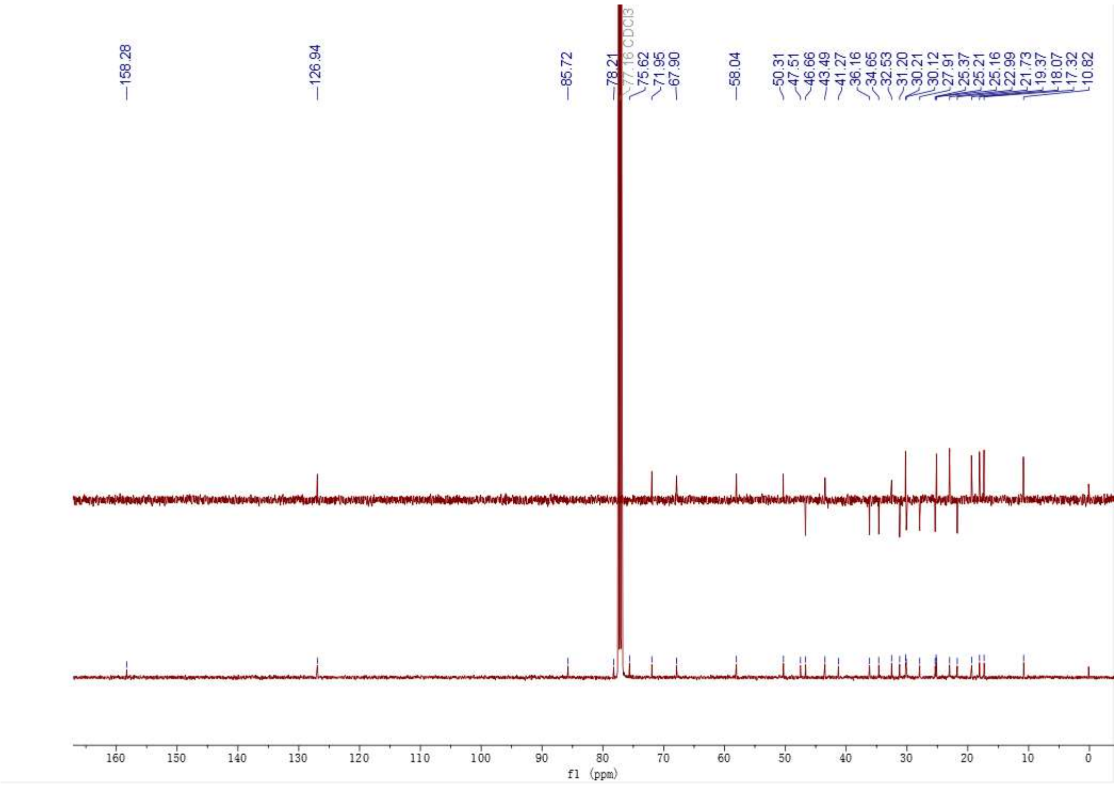

Figure S6.2. ^{13}C NMR spectrum (150 MHz) of compound **9** in $CDCl_3$.

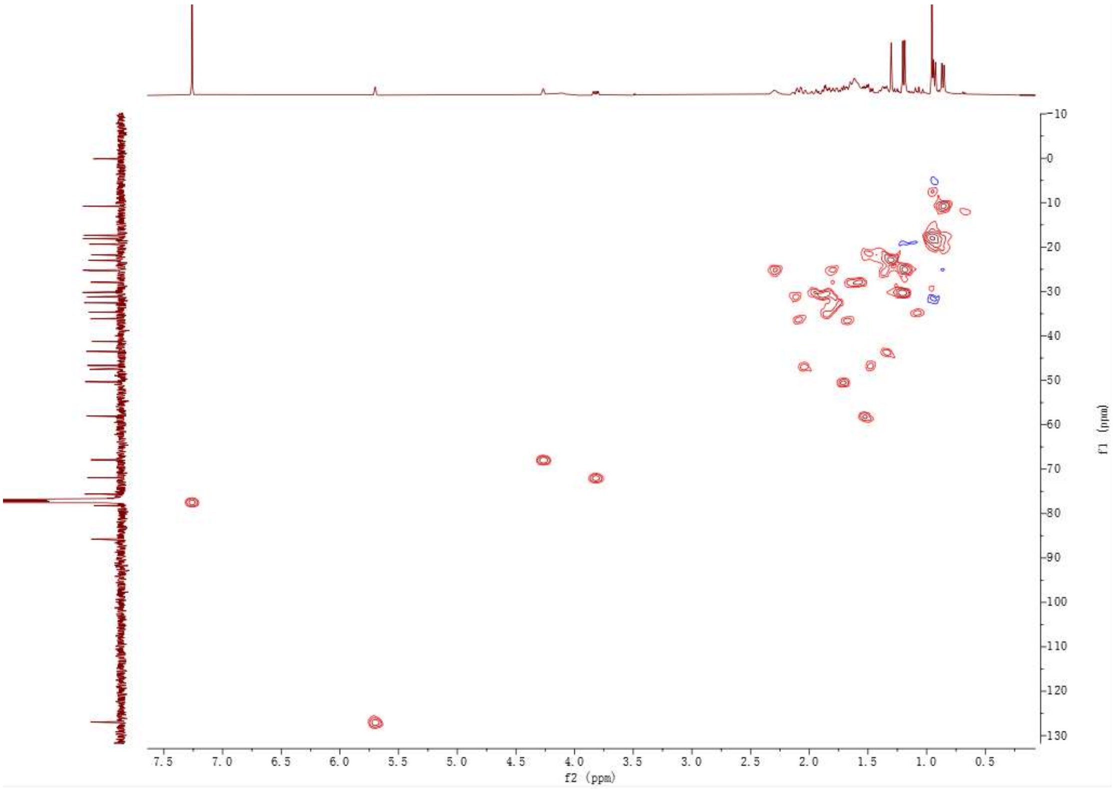

Figure S6.3. HSQC spectrum (600 MHz) of compound **9** in $CDCl_3$.

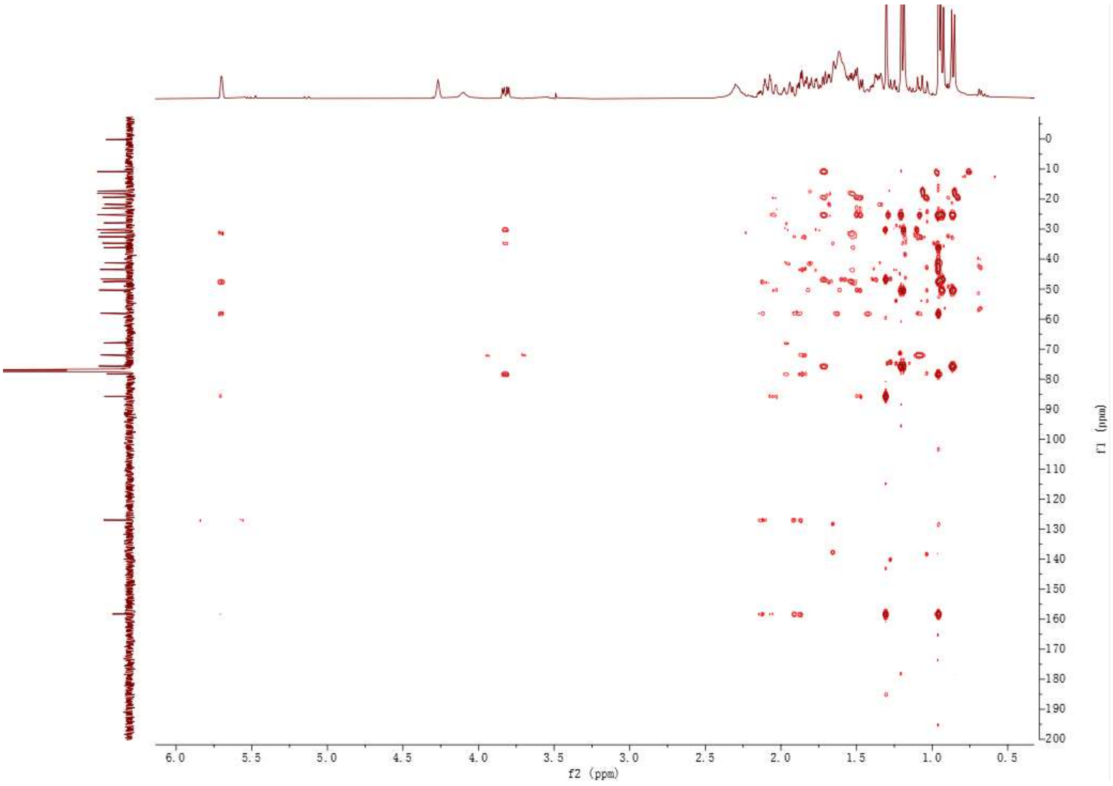

Figure S6.4. HMBC spectrum (600 MHz) of compound **9** in $CDCl_3$.

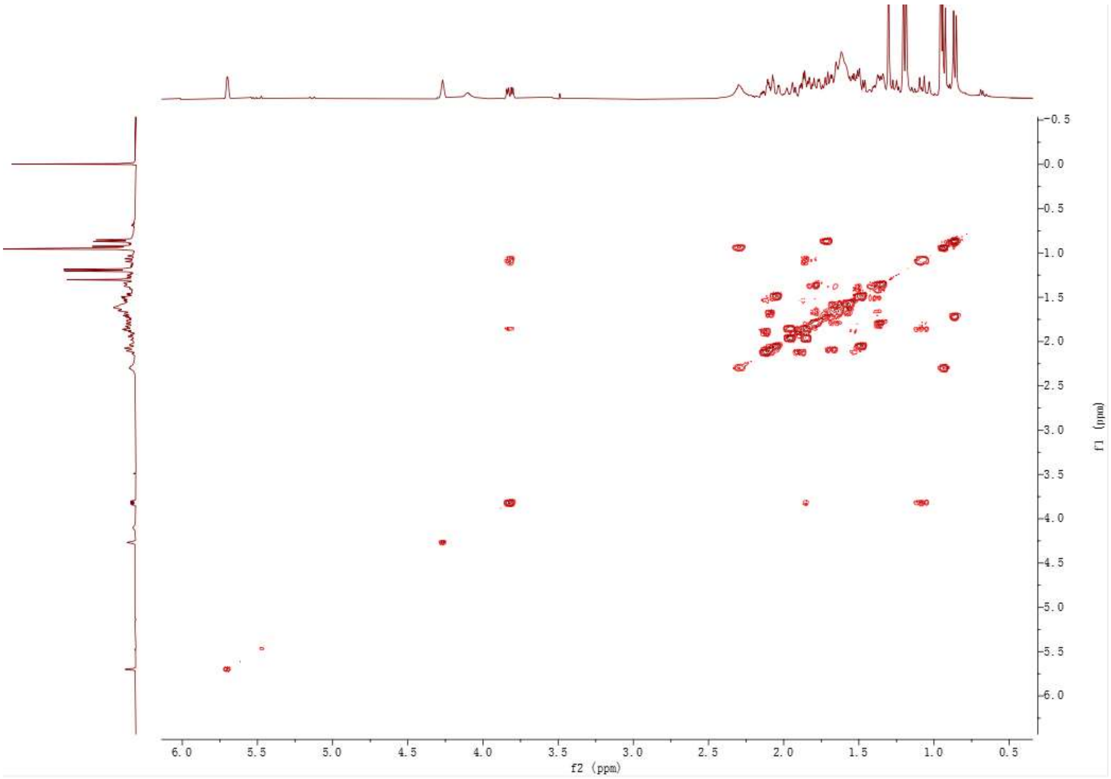

Figure S6.5. ^{1}H-^{1}H COSY spectrum (600 MHz) of compound **9** in $CDCl_3$.

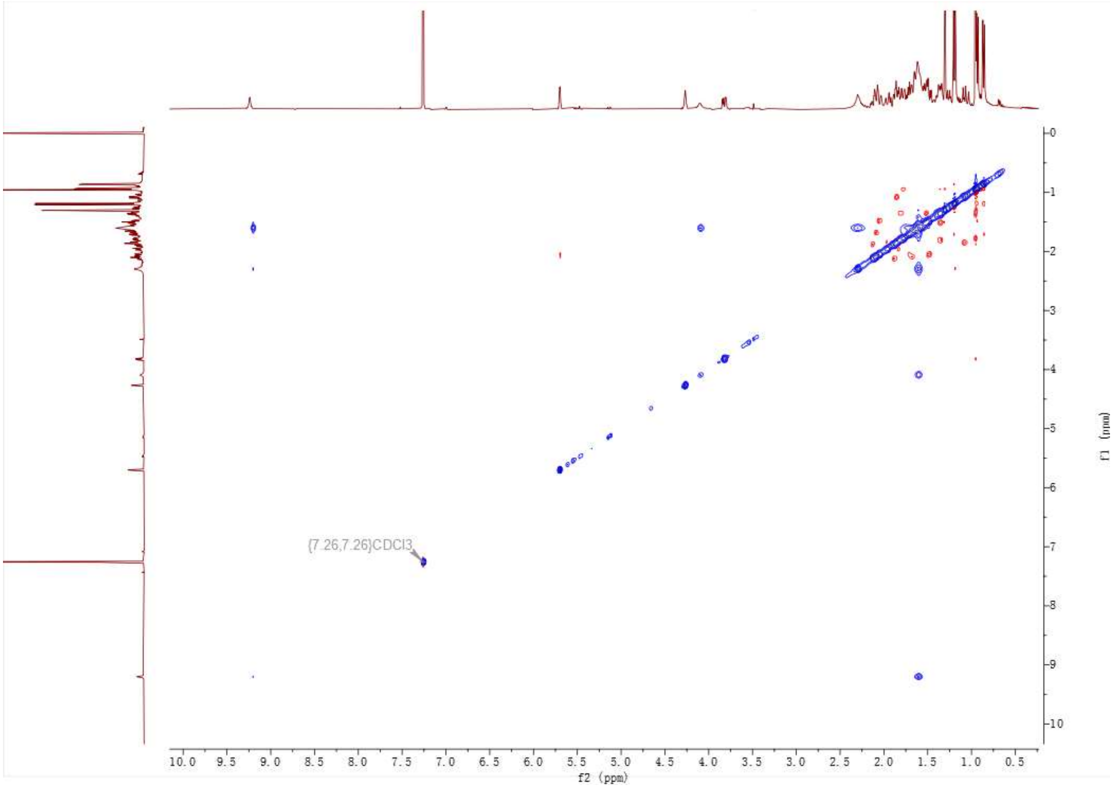

Figure S6.6. NOESY spectrum (600 MHz) of compound **9** in $CDCl_3$.

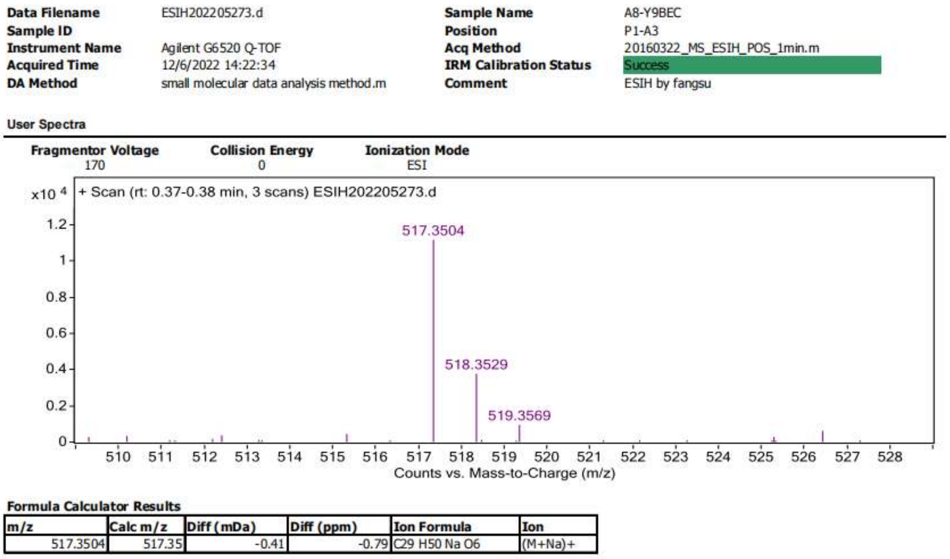

Figure S6.7. HR-ESIMS spectrum of compound **9**.

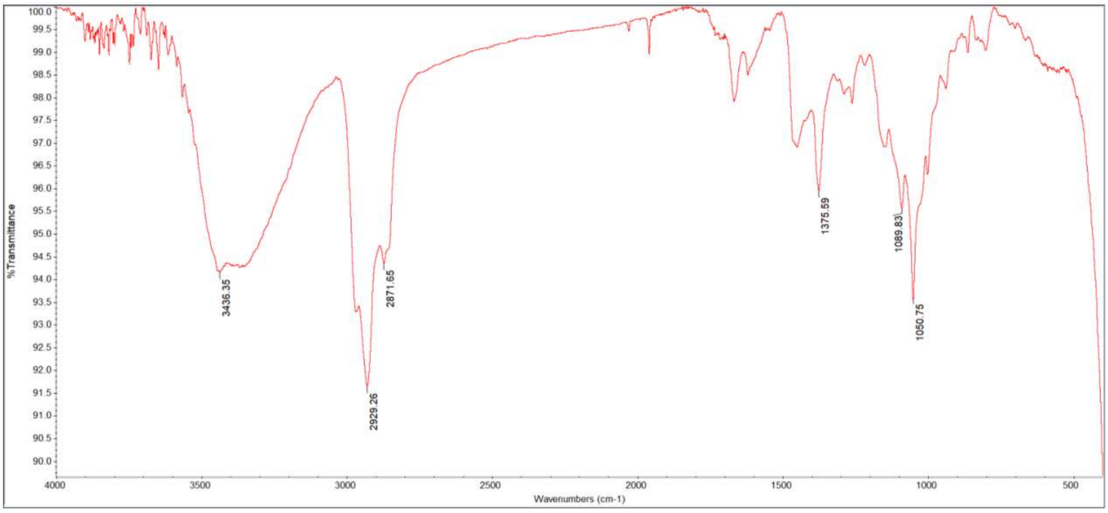

Figure S6.8. IR spectrum of compound **9**.

8. Spectra of compound 10

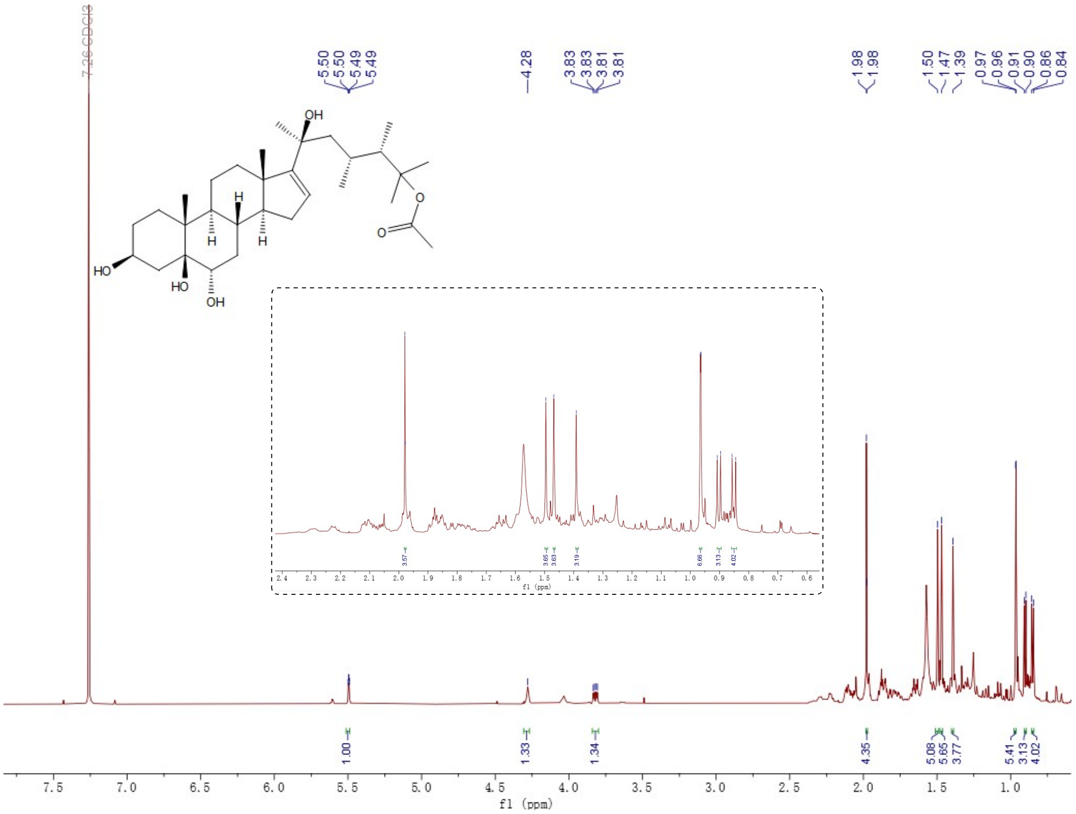

Figure S7.1. ^{1}H NMR spectrum (600 MHz) of compound **10** in $CDCl_3$.

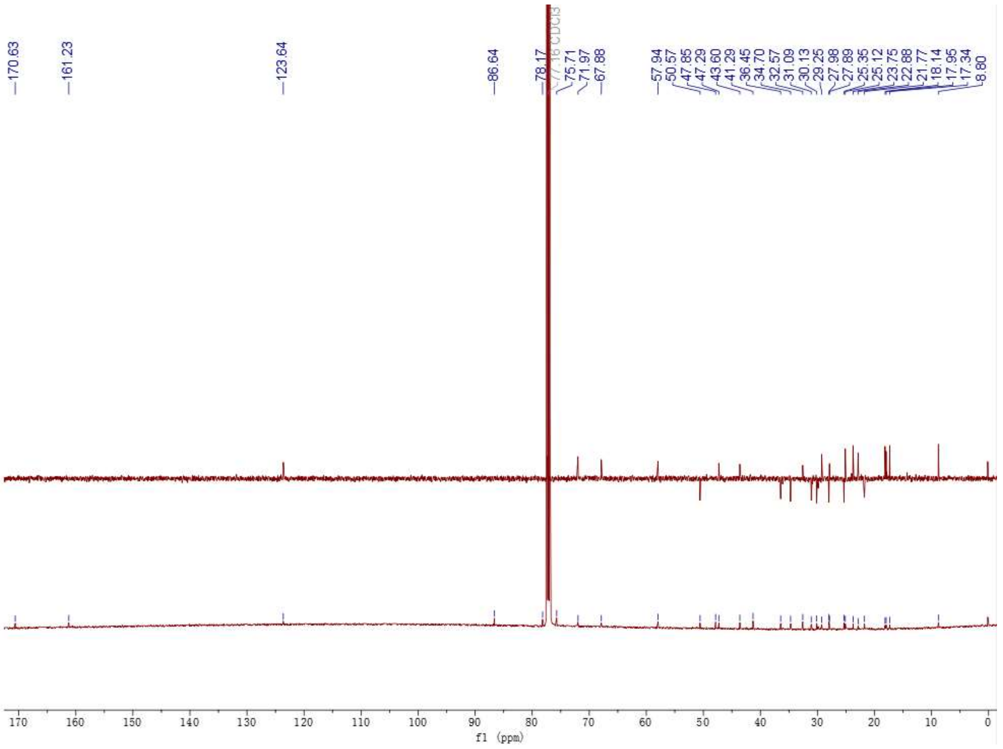

Figure S7.2. ^{13}C NMR spectrum (150 MHz) of compound **10** in $CDCl_3$.

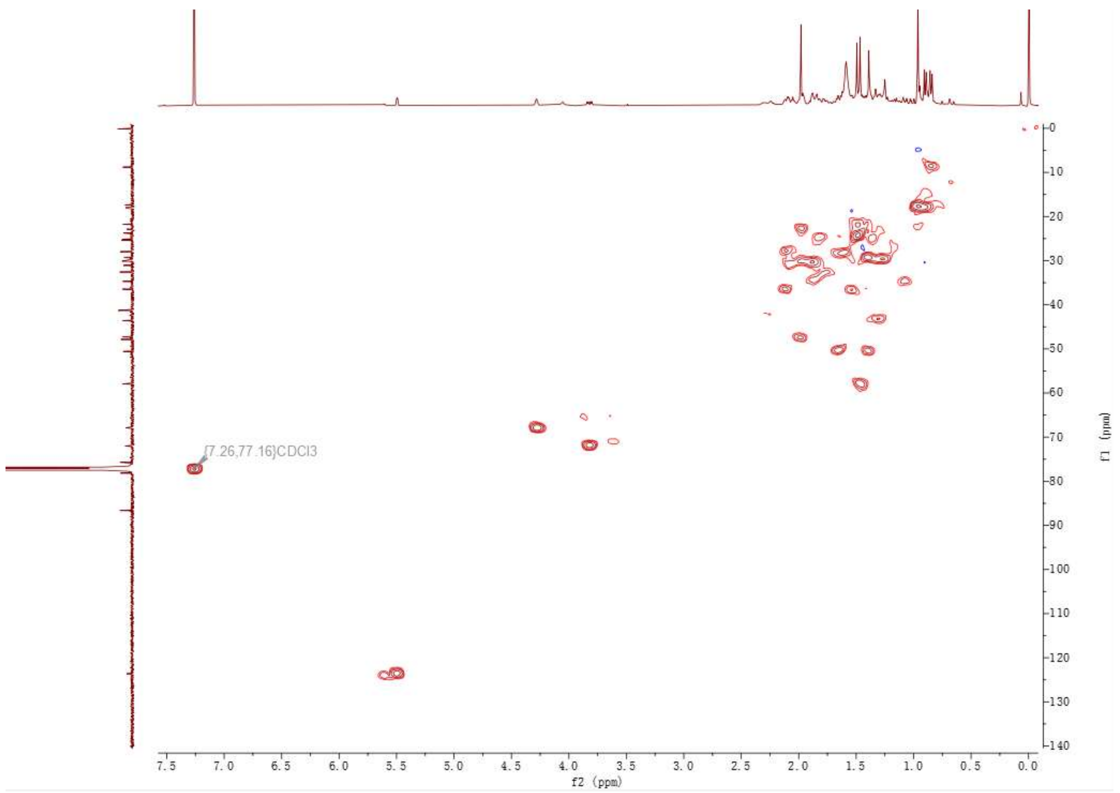

Figure S7.3. HSQC spectrum (600 MHz) of compound **10** in $CDCl_3$.

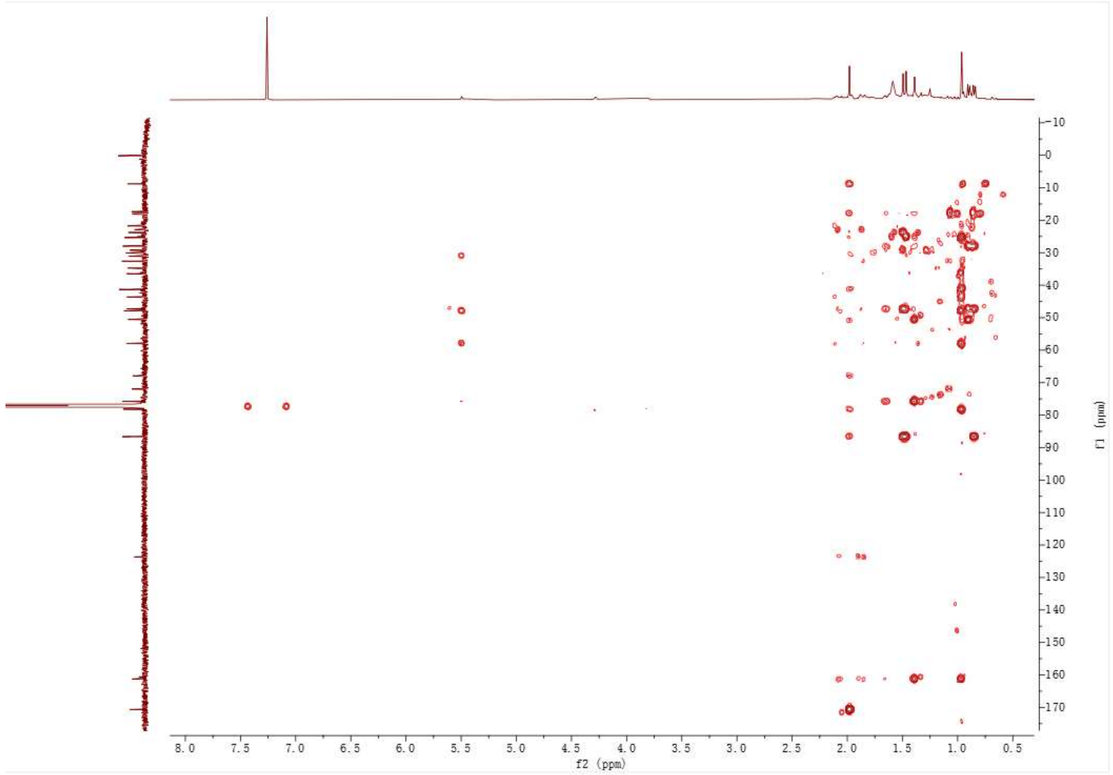

Figure S7.4. HMBC spectrum (600 MHz) of compound **10** in $CDCl_3$.

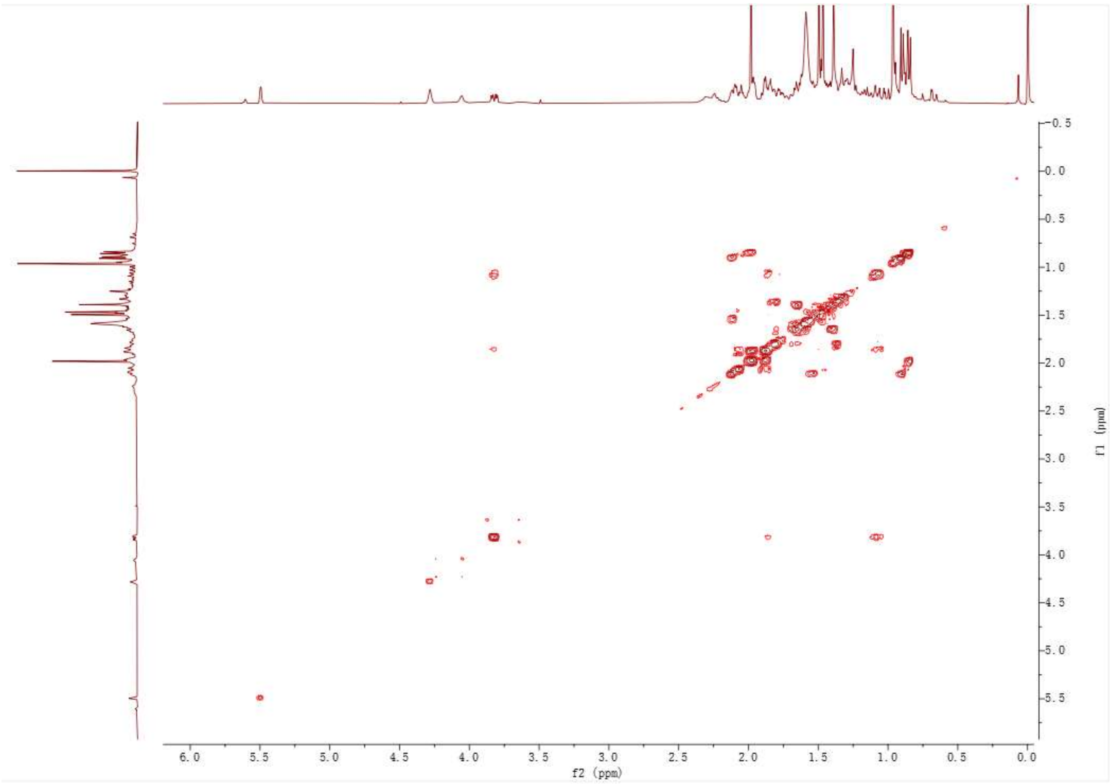

Figure S7.5. ^{1}H-^{1}H COSY spectrum (600 MHz) of compound **10** in $CDCl_3$.

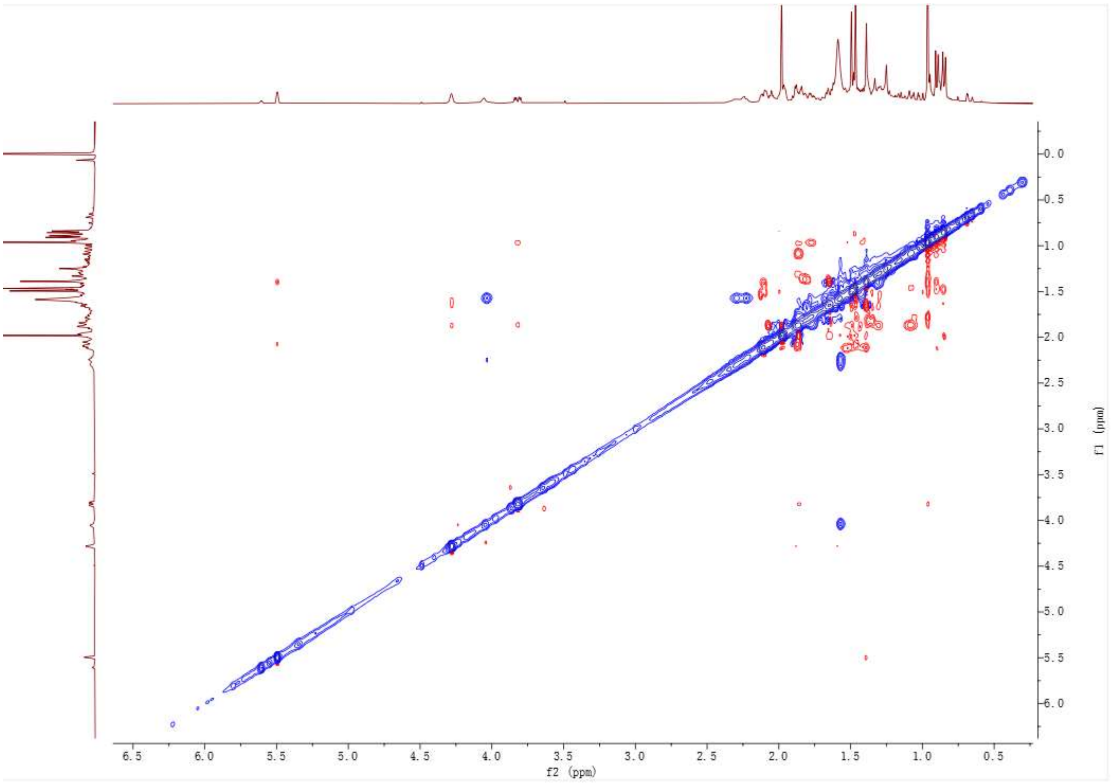

Figure S7.6. NOESY spectrum (600 MHz) of compound **10** in $CDCl_3$.

**Data Filename**	ESIH202205569.d	**Sample Name**	A8-Y9ADFGC
**Sample ID**		**Position**	P1-A4
**Instrument Name**	Agilent G6520 Q-TOF	**Acq Method**	20160322_MS_ESIH_POS_1min.m
**Acquired Time**	12/15/2022 10:27:19	**IRM Calibration Status**	Success
**DA Method**	small molecular data analysis method.m	**Comment**	ESIH by fangsu

User Spectra

**Fragmentor Voltage**	**Collision Energy**	**Ionization Mode**
170	0	ESI

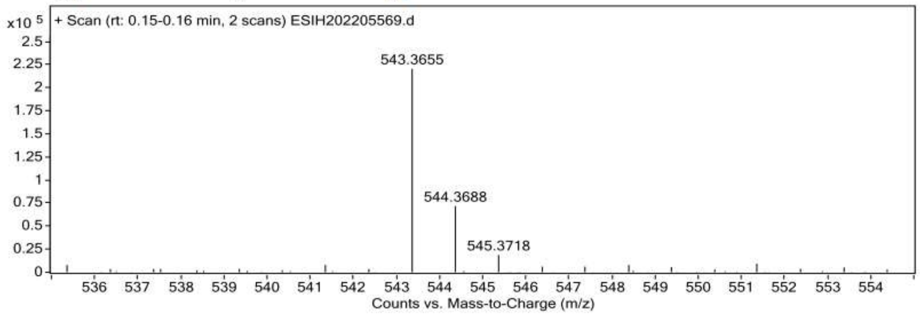

Formula Calculator Results

m/z	Calc m/z	Diff (mDa)	Diff (ppm)	Ion Formula	Ion
543.3655	543.3656	0.08	0.14	C31 H52 Na O6	(M+Na)+

Figure S7.7. HR-ESIMS spectrum of compound **10**.

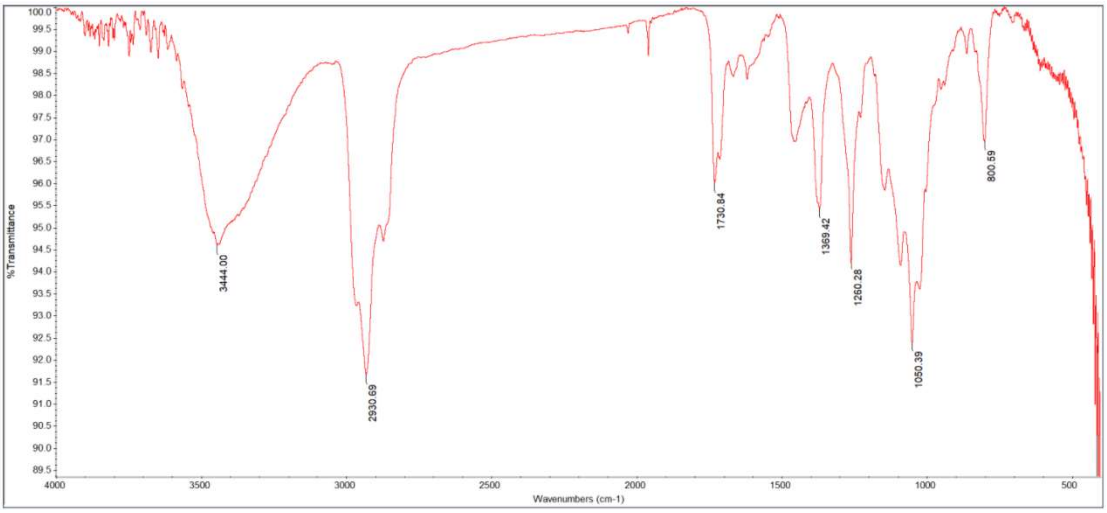

Figure S7.8. IR spectrum of compound **10**.

9. Spectra of compound 11

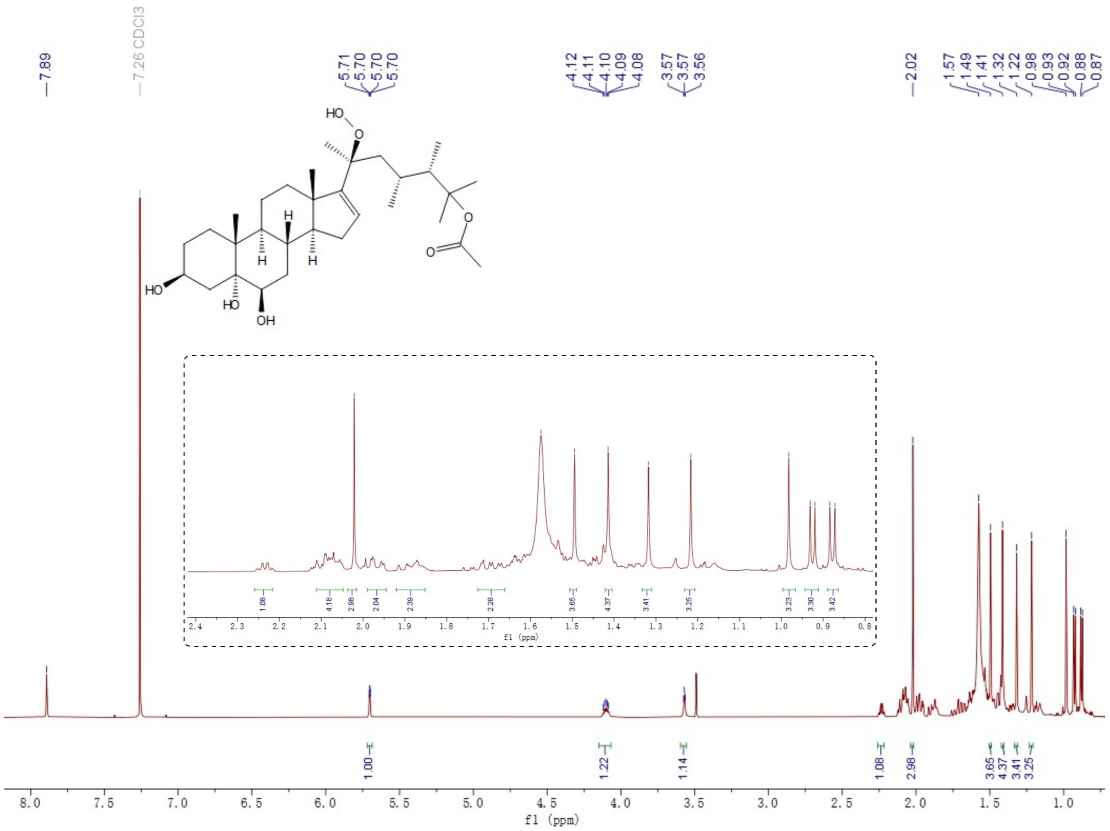

Figure S8.1. ^{1}H NMR spectrum (600 MHz) of compound **11** in $CDCl_3$.

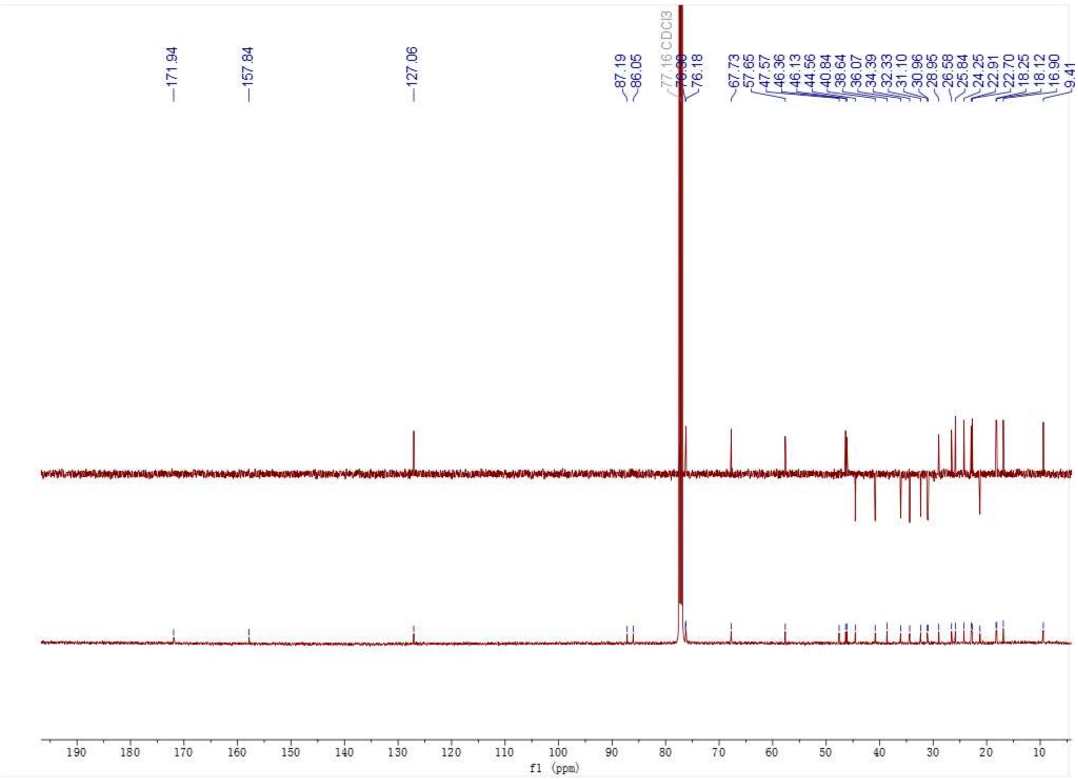

Figure S8.2. ^{13}C NMR spectrum (150 MHz) of compound **11** in $CDCl_3$.

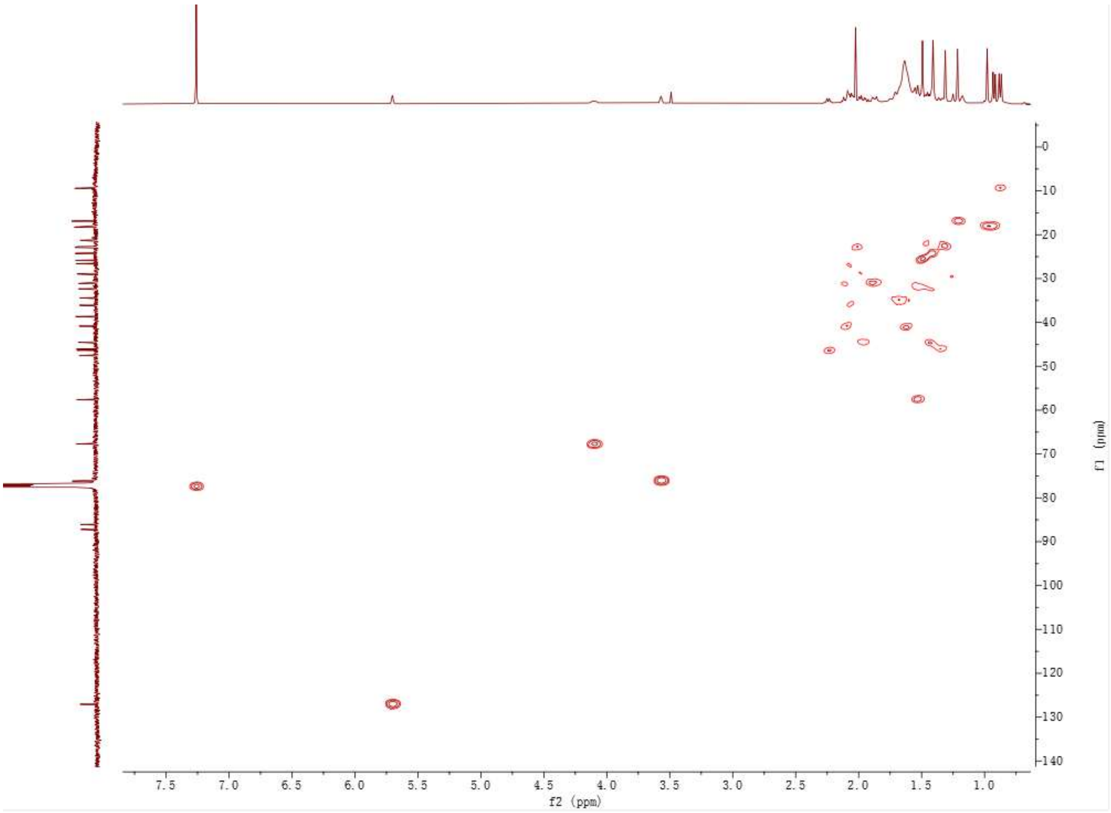

Figure S8.3. HSQC spectrum (600 MHz) of compound **11** in $CDCl_3$.

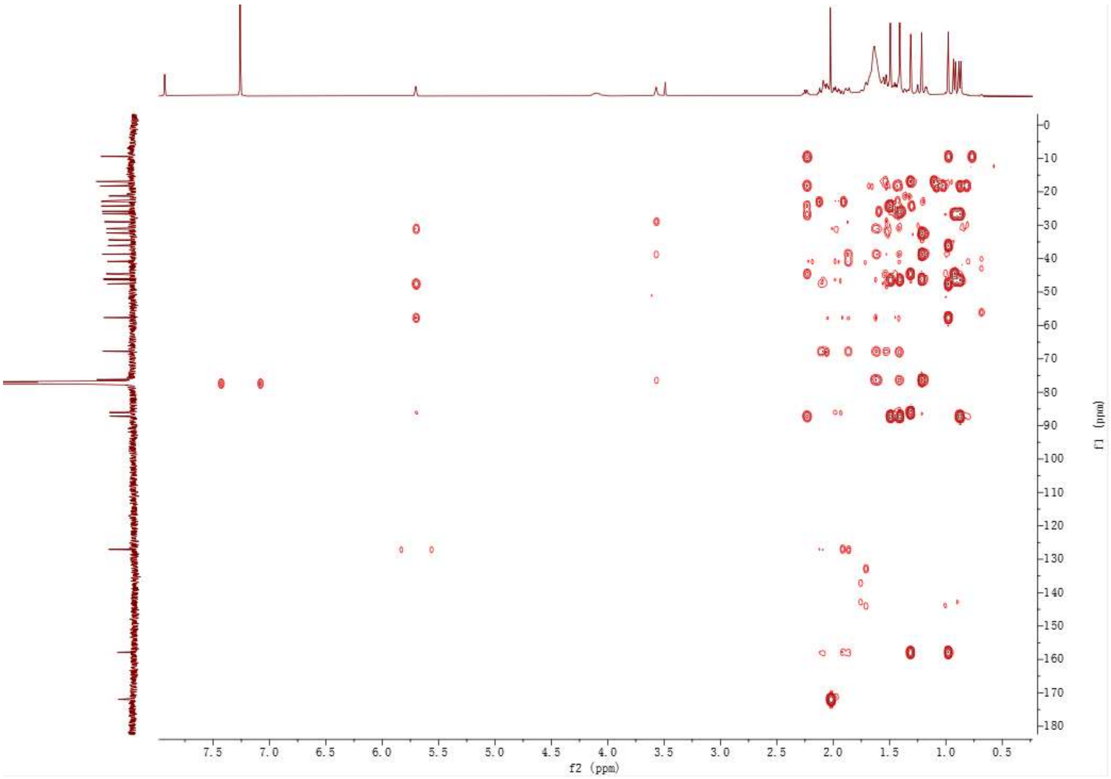

Figure S8.4. HMBC spectrum (600 MHz) of compound **11** in $CDCl_3$.

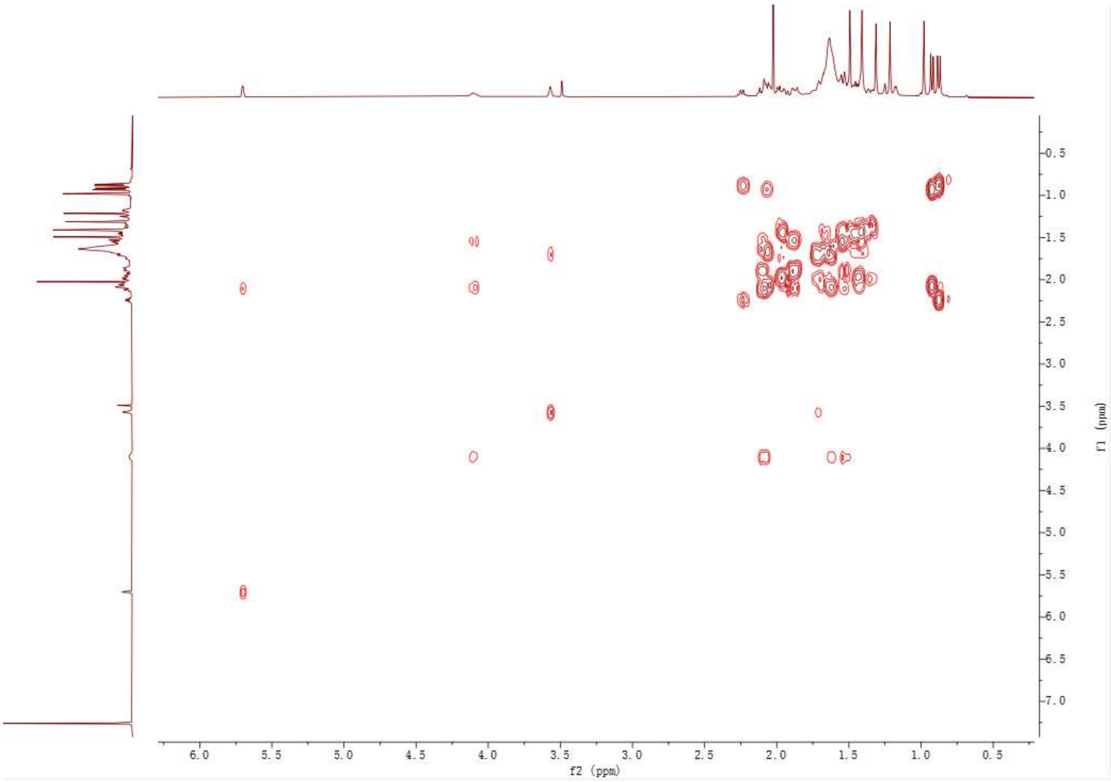

Figure S8.5. ^{1}H-^{1}H COSY spectrum (600 MHz) of compound **11** in $CDCl_3$.

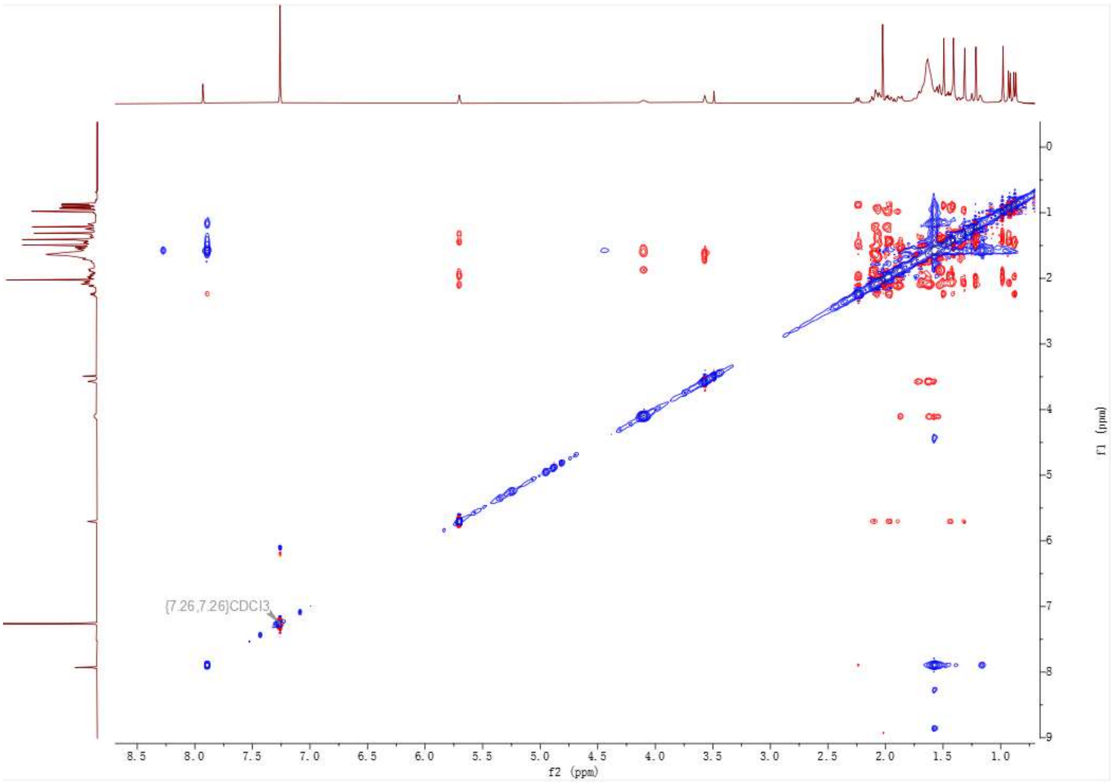

Figure S8.6. NOESY spectrum (600 MHz) of compound **11** in $CDCl_3$.

Data Filename	ESIH202205699.d	Sample Name	A8-A8-Y9ADFE
Sample ID		Position	P1-D9
Instrument Name	Agilent G6520 Q-TOF	Acq Method	20160322_MS_ESIH_POS_1min.m
Acquired Time	12/16/2022 12:37:30	IRM Calibration Status	Success
DA Method	small molecular data analysis method.m	Comment	ESIH by fangsu

User Spectra

Fragmentor Voltage	Collision Energy	Ionization Mode
170	0	ESI

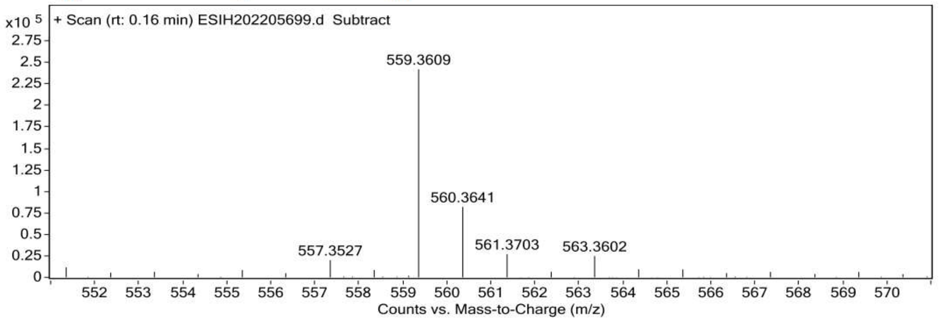

Formula Calculator Results

m/z	Calc m/z	Diff (mDa)	Diff (ppm)	Ion Formula	Ion
559.3609	559.3605	-0.35	-0.62	C31 H52 Na O7	(M+Na)+

Figure S8.7. HR-ESIMS spectrum of compound **11**.

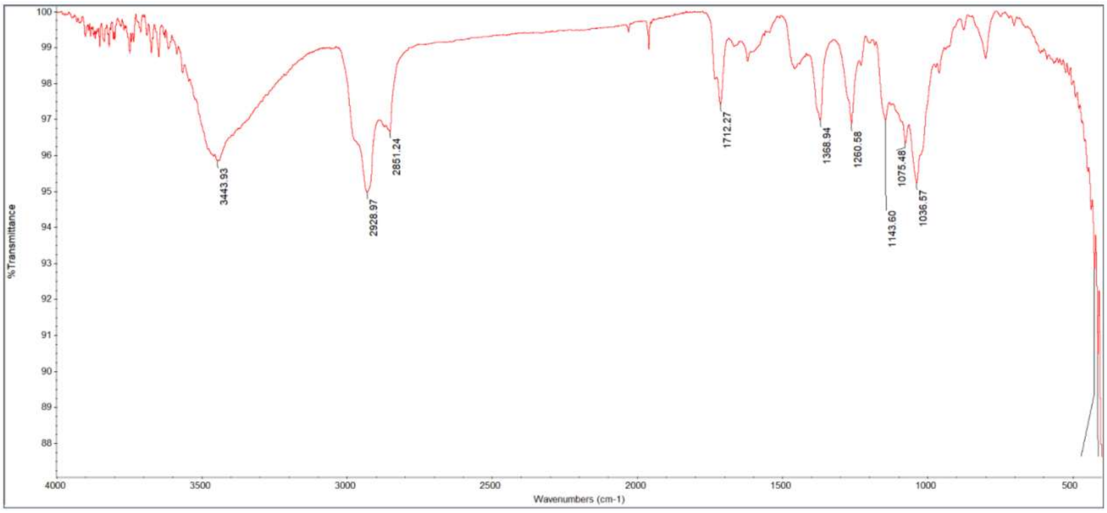

Figure S8.8. IR spectrum of compound **11**.

10.Spectra of compound 12

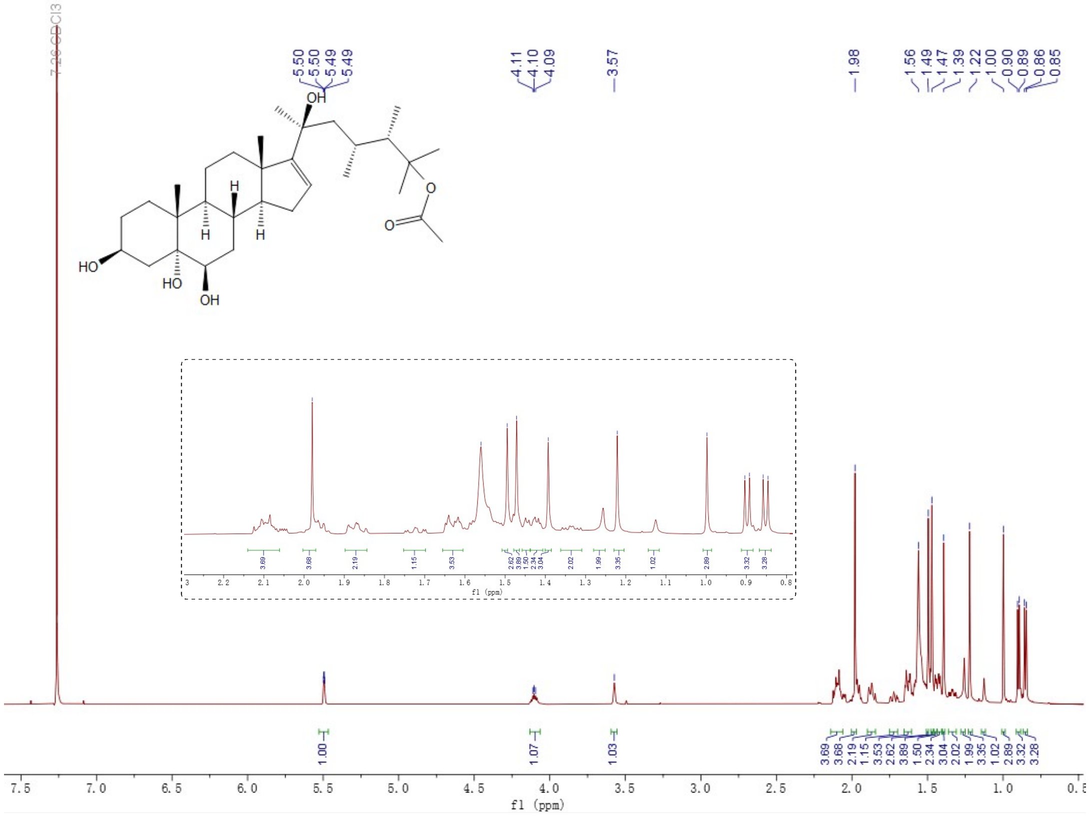

Figure S9.1. ^{1}H NMR spectrum (600 MHz) of compound **12** in $CDCl_3$.

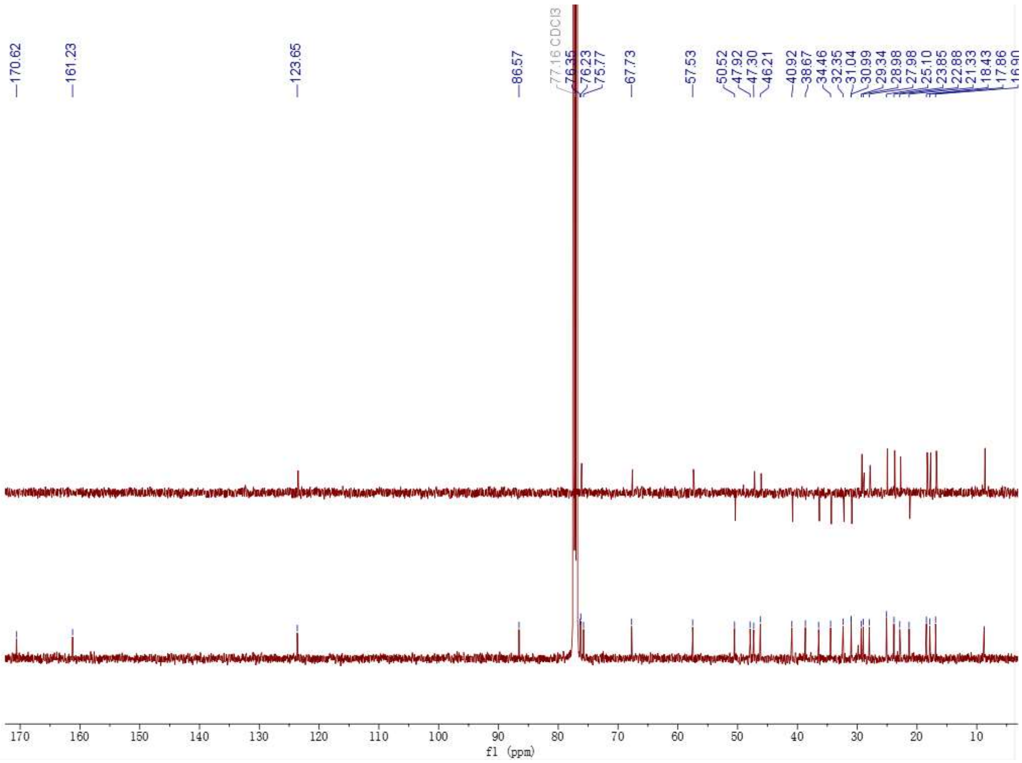

Figure S9.2. ^{13}C NMR spectrum (150 MHz) of compound **12** in $CDCl_3$.

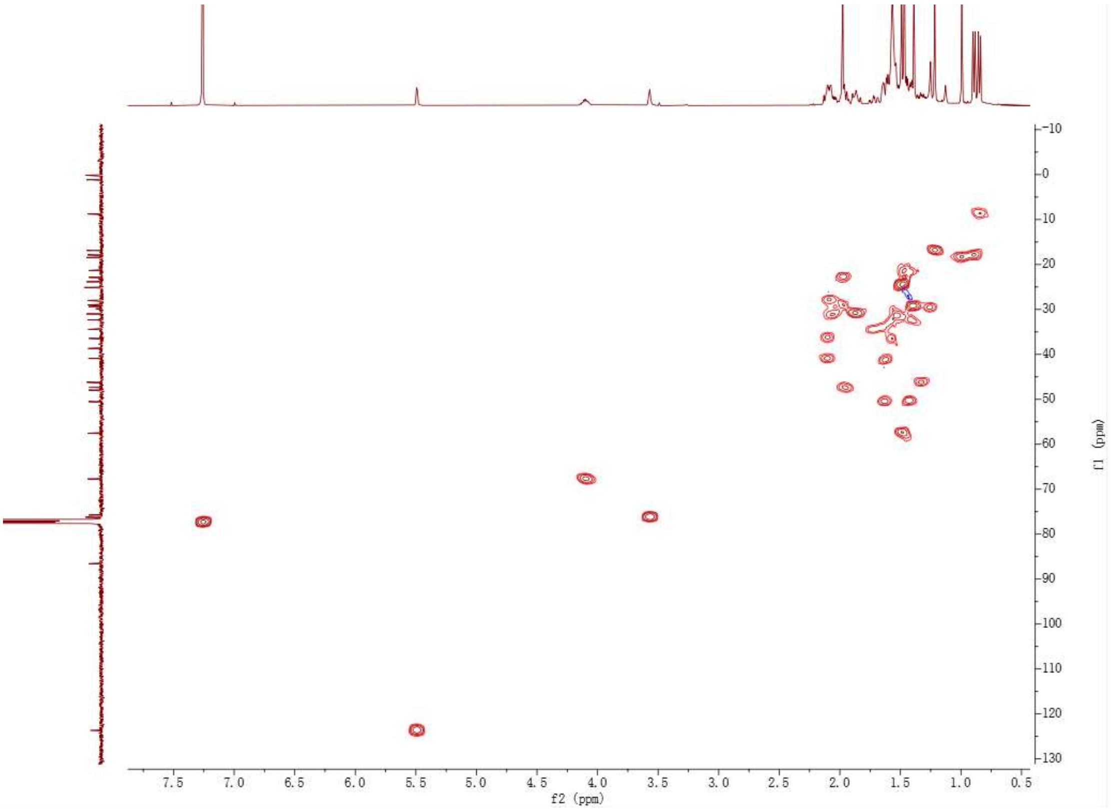

Figure S9.3. HSQC spectrum (600 MHz) of compound **12** in $CDCl_3$.

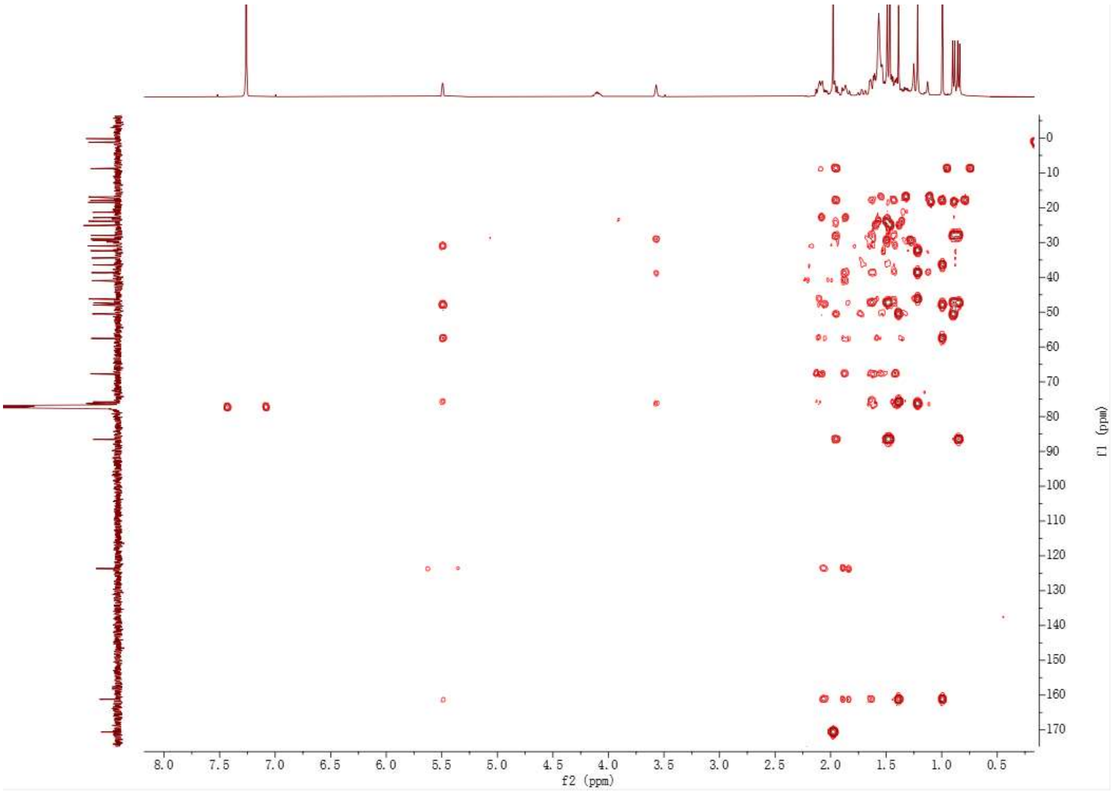

Figure S9.4. HMBC spectrum (600 MHz) of compound **12** in $CDCl_3$.

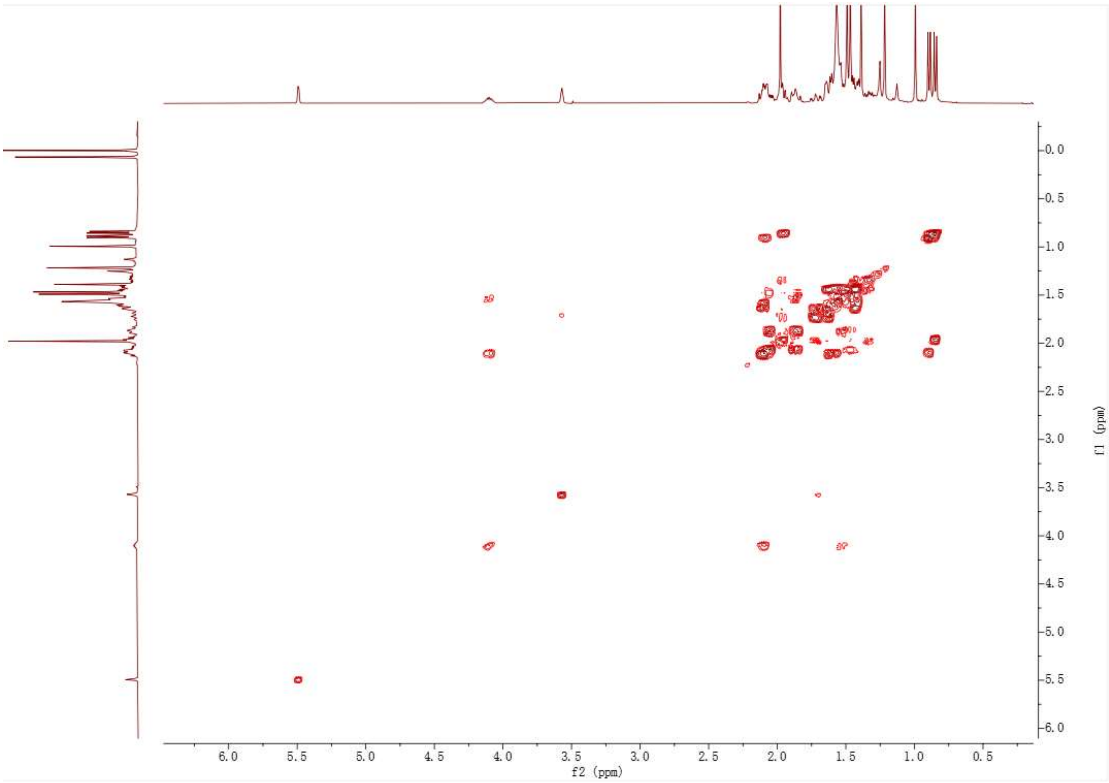

Figure S9.5. ^{1}H-^{1}H COSY spectrum (600 MHz) of compound **12** in $CDCl_3$.

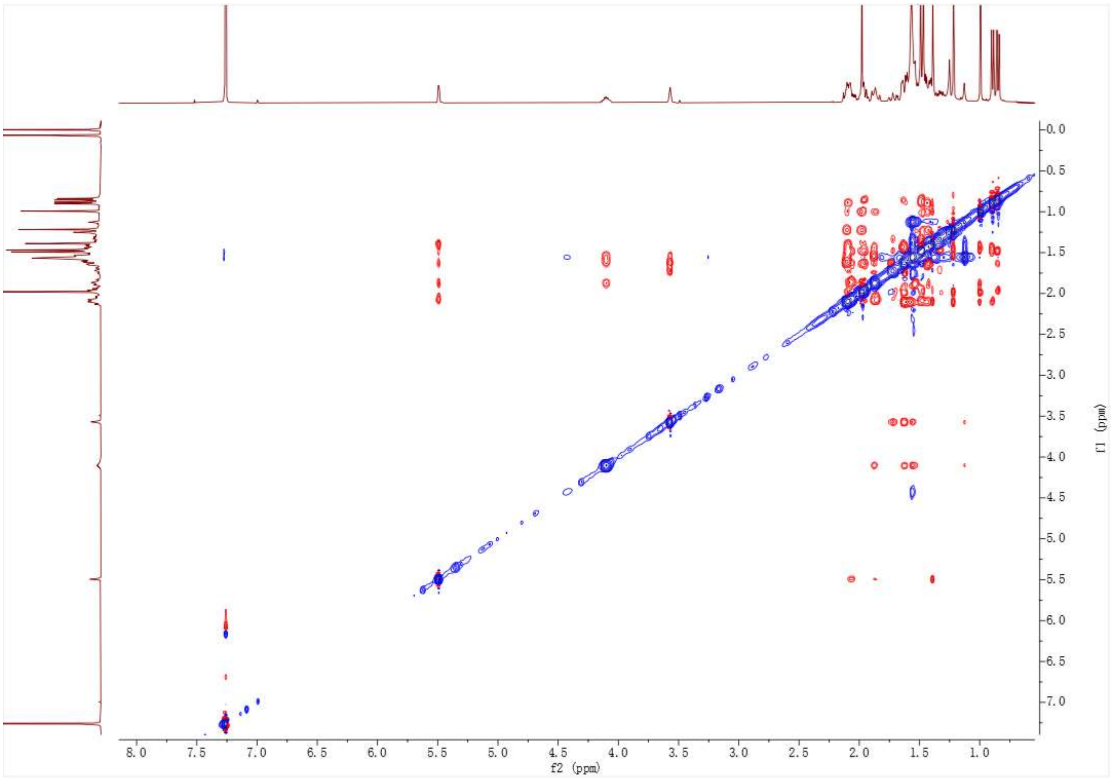

Figure S9.6. NOESY spectrum (600 MHz) of compound **12** in $CDCl_3$.

**Data Filename**	ESIH202205698.d	**Sample Name**	A8-Y9AECA
**Sample ID**		**Position**	P1-D8
**Instrument Name**	Agilent G6520 Q-TOF	**Acq Method**	20160322_MS_ESIH_POS_1min.m
**Acquired Time**	12/16/2022 12:36:15	**IRM Calibration Status**	Success
**DA Method**	small molecular data analysis method.m	**Comment**	ESIH by fangsu

User Spectra

Fragmentor Voltage	Collision Energy	Ionization Mode
170	0	ESI

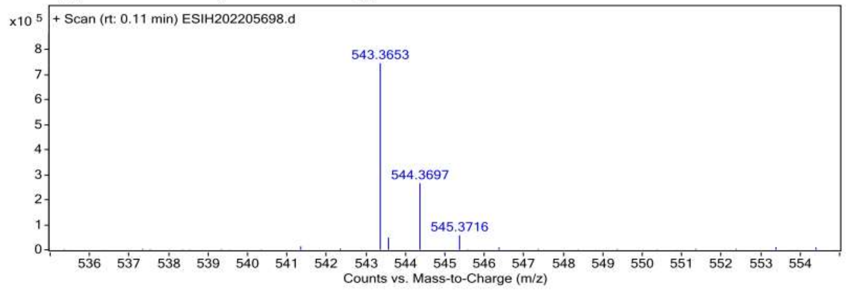

Formula Calculator Results

m/z	Calc m/z	Diff (mDa)	Diff (ppm)	Ion Formula	Ion
543.3653	543.3656	0.26	0.48	C31 H52 Na O6	(M+Na)+

Figure S9.7. HR-ESIMS spectrum of compound **12**.

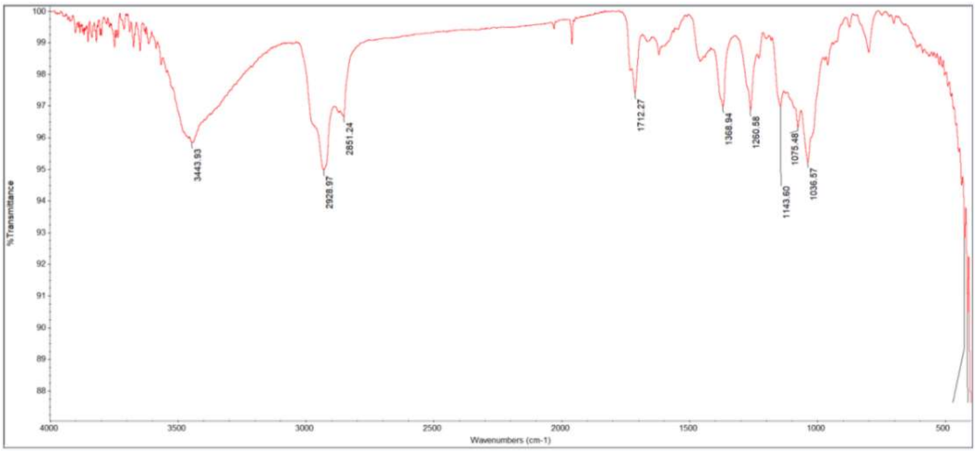

Figure S9.8. IR spectrum of compound **12**.

11.Spectra of compound 15a

**Data Filename**	ESIH202300996.d	**Sample Name**	A8-Y9BCCC
**Sample ID**		**Position**	P1-A2
**Instrument Name**	Agilent G6520 Q-TOF	**Acq Method**	20160322_MS_ESIH_POS_1min.m
**Acquired Time**	2/16/2023 15:05:15	**IRM Calibration Status**	Success
**DA Method**	small molecular data analysis method.m	**Comment**	ESIH by fangsu

User Spectra

Fragmentor Voltage	Collision Energy	Ionization Mode
175	0	ESI

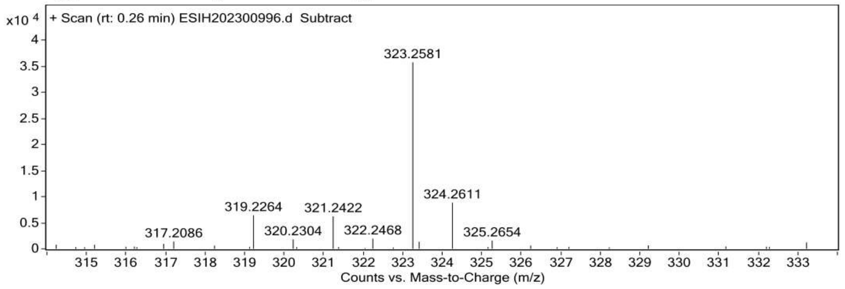

Formula Calculator Results

m/z	Calc m/z	Diff (mDa)	Diff (ppm)	Ion Formula	Ion
323.2581	323.2581	-0.01	-0.02	C20 H35 O3	(M+H)+

Figure S10.1. HR-ESIMS spectrum of compound **15a**.

12.QM-NMR calculation

Torsional sampling (MCMM) conformational searches using OPLS_2005 force field were carried out by the means of conformational search module in Macro model 9.9.223 software (Schrodinger, http://www.schrodinger.com/MacroModel), applying an energy window of 21 kJ/mol (5.02 kcal/mol) for saving structures. Conformers above 1% population were re-optimized with Gaussian 09 at the B3LYP/6-311G(d,p) level with IEFPCM solvent model for acetonitrile.

The following DFT calculations were performed using Gaussian 09, and the dominant conformers were re-optimized at B3LYP/6-311G(d,p) level of theory. Magnetic shielding constants (σ) were calculated by means of the gauge including atomic orbitals (GIAO) method at mPW1PW91/6–31 G(d) level of theory as recommended for DP4+ analysis.

12.1. Structure of studied isomers of compound **3**.

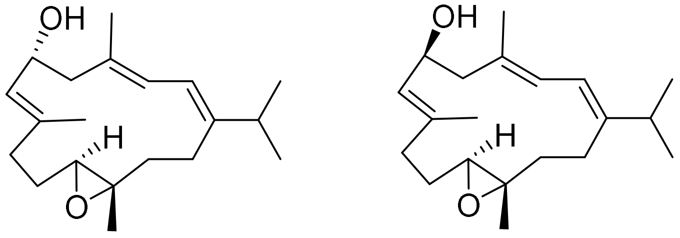

3a
isomer 1

3b
isomer 2

Figure S11.1. Two possible configurations of compound **3**.

12.2. SCF energies computed at the PCM/mPW1PW91/6-31G* level of theory for all conformers of studied isomers.

3a_6R11S12S_1: SCF energy (PCM/mPW1PW91/6-31G*) = -931.56192291 a. u.

3a_6R11S12S_2: SCF energy (PCM/mPW1PW91/6-31G*) = -931.56473866 a. u.

3a_6R11S12S_3: SCF energy (PCM/mPW1PW91/6-31G*) = -931.56474077 a. u.

3a_6R11S12S_4: SCF energy (PCM/mPW1PW91/6-31G*) = -931.56150640 a. u.

3a_6R11S12S_5: SCF energy (PCM/mPW1PW91/6-31G*) = -931.56244883 a. u.

3a_6R11S12S_6: SCF energy (PCM/mPW1PW91/6-31G*) = -931.56399453 a. u.

3a_6R11S12S_7: SCF energy (PCM/mPW1PW91/6-31G*) = -931.56239668 a. u.

3a_6R11S12S_8: SCF energy (PCM/mPW1PW91/6-31G*) = -931.56355852 a. u.

3a_6R11S12S_9: SCF energy (PCM/mPW1PW91/6-31G*) = -931.55897310 a. u.

3b_6S11S12S_1: SCF energy (PCM/mPW1PW91/6-31G*) = -931.55843435 a. u.

3b_6S11S12S_2: SCF energy (PCM/mPW1PW91/6-31G*) = -931.55519468 a. u.

3b_6S11S12S_3: SCF energy (PCM/mPW1PW91/6-31G*) = -931.55217040 a. u.

3b_6S11S12S_4: SCF energy (PCM/mPW1PW91/6-31G*) = -931.55473068 a. u.

3b_6S11S12S_5: SCF energy (PCM/mPW1PW91/6-31G*) = -931.55773570 a. u.

3b_6S11S12S_6: SCF energy (PCM/mPW1PW91/6-31G*) = -931.55806553 a. u.

3b_6S11S12S_7: SCF energy (PCM/mPW1PW91/6-31G*) = -931.55198124 a. u.

3b_6S11S12S_8: SCF energy (PCM/mPW1PW91/6-31G*) = -931.55451696 a. u.

3b_6S11S12S_9: SCF energy (PCM/mPW1PW91/6-31G*) = -931.55131077 a. u.

3b_6S11S12S_10: SCF energy (PCM/mPW1PW91/6-31G*) = -931.55806506 a. u.

12.3. DP4+ results obtained using experimental ^{1}H and ^{13}C data of compound **3** versus isomers **3a-3b**.

Functional	Solvent?		Basis Set		Type of Data	
mPW1PW91	PCM		6-31G(d)		Shielding Tensors	
	Isomer 1	Isomer 2	Isomer 3	Isomer 4	Isomer 5	Isomer 6
sDP4+ (H data)	100.00%	0.00%	-	-	-	-
sDP4+ (C data)	67.24%	32.76%	-	-	-	-
sDP4+ (all data)	100.00%	0.00%	-	-	-	-
uDP4+ (H data)	100.00%	0.00%	-	-	-	-
uDP4+ (C data)	55.50%	44.50%	-	-	-	-
uDP4+ (all data)	100.00%	0.00%	-	-	-	-
DP4+ (H data)	100.00%	0.00%	-	-	-	-
DP4+ (C data)	71.91%	28.09%	-	-	-	-
DP4+ (all data)	100.00%	0.00%	-	-	-	-

12.4. Structure of studied isomers of compound **4**.

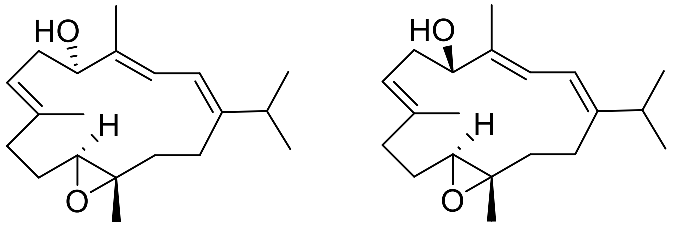

4a
isomer 1

4b
isomer 2

Figure S11.2. Two possible configurations of compound **4**.

12.5. SCF energies computed at the PCM/mPW1PW91/6-31G* level of theory for all conformers of studied isomers.

4a_5R11S12S_1: SCF energy (PCM/mPW1PW91/6-31G*) = -931.56095132 a. u.

4a_5R11S12S_2: SCF energy (PCM/mPW1PW91/6-31G*) = -931.56479510 a. u.

4a_5R11S12S_3: SCF energy (PCM/mPW1PW91/6-31G*) = -931.56033711 a. u.

4a_5R11S12S_4: SCF energy (PCM/mPW1PW91/6-31G*) = -931.56528750 a. u.

4a_5R11S12S_5: SCF energy (PCM/mPW1PW91/6-31G*) = -931.55940219 a. u.

4a_5R11S12S_6: SCF energy (PCM/mPW1PW91/6-31G*) = -931.56486809 a. u.

4a_5R11S12S_7: SCF energy (PCM/mPW1PW91/6-31G*) = -931.56362976 a. u.

4a_5R11S12S_8: SCF energy (PCM/mPW1PW91/6-31G*) = -931.56321472 a. u.

4a_5R11S12S_9: SCF energy (PCM/mPW1PW91/6-31G*) = -931.55994482 a. u.

4a_5R11S12S_10: SCF energy (PCM/mPW1PW91/6-31G*) = -931.56227741 a. u.

4b_5S11S12S_1: SCF energy (PCM/mPW1PW91/6-31G*) = -931.56559011 a. u.

4b_5S11S12S_2: SCF energy (PCM/mPW1PW91/6-31G*) = -931.56612488 a. u.

4b_5S11S12S_3: SCF energy (PCM/mPW1PW91/6-31G*) = -931.56559008 a. u.

4b_5S11S12S_4: SCF energy (PCM/mPW1PW91/6-31G*) = -931.56513833 a. u.

4b_5S11S12S_5: SCF energy (PCM/mPW1PW91/6-31G*) = -931.56568095 a. u.

4b_5S11S12S_6: SCF energy (PCM/mPW1PW91/6-31G*) = -931.55884239 a. u.

4b_5S11S12S_7: SCF energy (PCM/mPW1PW91/6-31G*) = -931.56066577 a. u.

4b_5S11S12S_8: SCF energy (PCM/mPW1PW91/6-31G*) = -931.55451696 a. u.

4b_5S11S12S_9: SCF energy (PCM/mPW1PW91/6-31G*) = -931.56230067 a. u.

4b_5S11S12S_10: SCF energy (PCM/mPW1PW91/6-31G*) = -931.56376243 a. u.

12.6. DP4+ results obtained using experimental ^{1}H and ^{13}C data of compound **4** versus isomers **4a-4b**.

Functional	Solvent?	Basis Set	Type of Data		
mPW1PW91	PCM	6-31G(d)	Shielding Tensors		

	Isomer 1	Isomer 2	Isomer 3	Isomer 4	Isomer 5	Isomer 6
sDP4+ (H data)	96.82%	3.18%	–	–	–	–
sDP4+ (C data)	99.17%	0.83%	–	–	–	–
sDP4+ (all data)	99.97%	0.03%	–	–	–	–
uDP4+ (H data)	97.65%	2.35%	–	–	–	–
uDP4+ (C data)	98.70%	1.30%	–	–	–	–
uDP4+ (all data)	99.97%	0.03%	–	–	–	–
DP4+ (H data)	99.92%	0.08%	–	–	–	–
DP4+ (C data)	99.99%	0.01%	–	–	–	–
DP4+ (all data)	100.00%	0.00%	–	–	–	–

12.7. Structure of studied isomers of compound **5**.

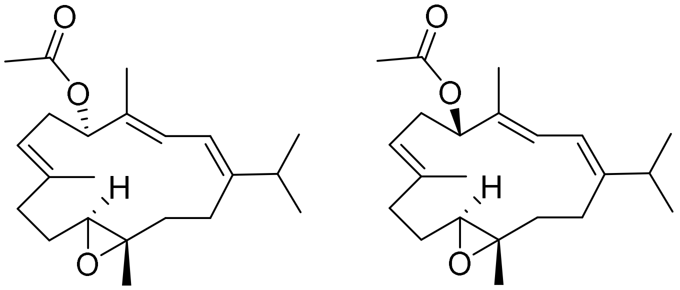

5a
isomer 1

5b
isomer 2

Figure S11.3. Two possible configurations of compound **5**.

12.8. SCF energies computed at the PCM/mPW1PW91/6-31G* level of theory for all conformers of studied isomers.

5a_5R11S12S_1: SCF energy (PCM/mPW1PW91/6-31G*) = -1084.18727565 a. u.

5a_5R11S12S_2: SCF energy (PCM/mPW1PW91/6-31G*) = -1084.19113432 a. u.

5a_5R11S12S_4: SCF energy (PCM/mPW1PW91/6-31G*) = -1084.19102676 a. u.

5a_5R11S12S_5: SCF energy (PCM/mPW1PW91/6-31G*) = -1084.19029245 a. u.

5a_5R11S12S_6: SCF energy (PCM/mPW1PW91/6-31G*) = -1084.18339270 a. u.

5a_5R11S12S_7: SCF energy (PCM/mPW1PW91/6-31G*) = -1084.18710949 a. u.

5a_5R11S12S_8: SCF energy (PCM/mPW1PW91/6-31G*) = -1084.18545285 a. u.

5a_5R11S12S_9: SCF energy (PCM/mPW1PW91/6-31G*) = -1084.18849334 a. u.

5a_5R11S12S_10: SCF energy (PCM/mPW1PW91/6-31G*) = -1084.18228246 a. u.

5b_5S11S12S_1: SCF energy (PCM/mPW1PW91/6-31G*) = -1084.18629387 a. u.

5b_5S11S12S_2: SCF energy (PCM/mPW1PW91/6-31G*) = -1084.18791705 a. u.

5b_5S11S12S_3: SCF energy (PCM/mPW1PW91/6-31G*) = -1084.18759467 a. u.

5b_5S11S12S_4: SCF energy (PCM/mPW1PW91/6-31G*) = -1084.18796802a. u.

5b_5S11S12S_5: SCF energy (PCM/mPW1PW91/6-31G*) = -1084.18761750 a. u.

5b_5S11S12S_6: SCF energy (PCM/mPW1PW91/6-31G*) = -1084.18735303 a. u.

5b_5S11S12S_7: SCF energy (PCM/mPW1PW91/6-31G*) = -1084.18695579 a. u.

5b_5S11S12S_8: SCF energy (PCM/mPW1PW91/6-31G*) = -1084.18614398 a. u.

5b_5S11S12S_9: SCF energy (PCM/mPW1PW91/6-31G*) = -1084.19250519 a. u.

5b_5S11S12S_10: SCF energy (PCM/mPW1PW91/6-31G*) = -1084.18269693 a. u.

5b_5S11S12S_11: SCF energy (PCM/mPW1PW91/6-31G*) = -1084.19269867 a. u.

5b_5S11S12S_12: SCF energy (PCM/mPW1PW91/6-31G*) = -1084.18521306 a. u

12.9. DP4+ results obtained using experimental ^{13}C data of compound **5** versus isomers **5a-5b**.

Functional	Solvent?		Basis Set		Type of Data	
mPW1PW91	PCM		6-31G(d)		Shielding Tensors	
	Isomer 1	Isomer 2	Isomer 3	Isomer 4	Isomer 5	Isomer 6
sDP4+ (H data)	–	–	–	–	–	–
sDP4+ (C data)	81.76%	18.24%	–	–	–	–
sDP4+ (all data)	81.76%	18.24%	–	–	–	–
uDP4+ (H data)	–	–	–	–	–	–
uDP4+ (C data)	97.89%	2.11%	–	–	–	–
uDP4+ (all data)	97.89%	2.11%	–	–	–	–
DP4+ (H data)	–	–	–	–	–	–
DP4+ (C data)	99.52%	0.48%	–	–	–	–
DP4+ (all data)	99.52%	0.48%	–	–	–	–

13.TDDFT-ECD calculation

Conformational searches were done following the general protocols previously described for DP4+ calculation. For the resulting geometries, ECD spectra were obtained by TDDFT calculations performed with the same functional, basis set and solvent model as the energy optimization. Finally, the Boltzmann-averaged ECD spectra of **3**, **4**, **5** was obtained with SpecDis1.62 software.

Re-optimized conformers of (6*R*, 11*S*, 12*S*)-**3**, (5S, 11*R*, 12*R*)-**4** and (5S, 11*R*, 12*R*)-**5** calculated at the B3LYP/6-311G(d,p) level with IEFPCM solvent model for acetonitrile.

(6*R*,11*S*,*12S*)-**3:**

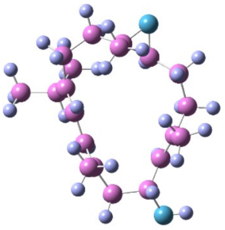

Conf.1
46.351%
-932.02316965

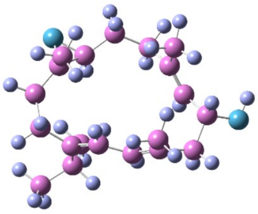

Conf.2
6.996%
-932.02637025

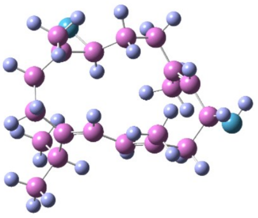

Conf.3
6.069%
-932.02637081

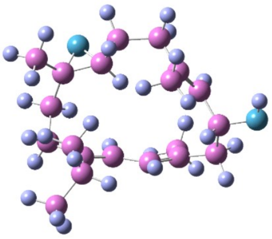

Conf.4
4.414%
-932.02275117

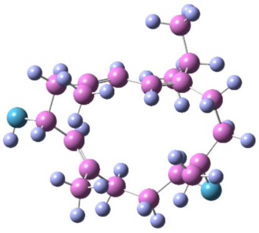

Conf.5
3.548%
-932.02360346

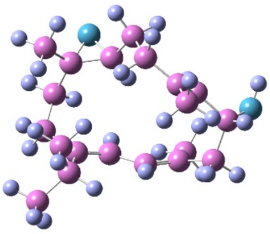

Conf.6
2.465%
-932.02549192

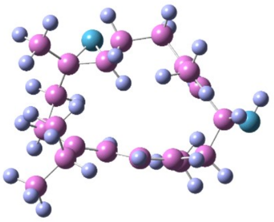

Conf.7
2.238%
-932.02367638

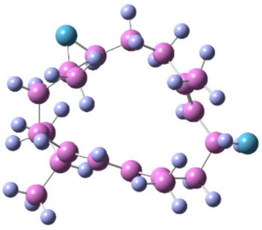

Conf.8
1.970%
-932.02536473

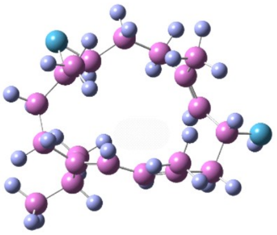

Conf.9
1.928%
-932.02536480

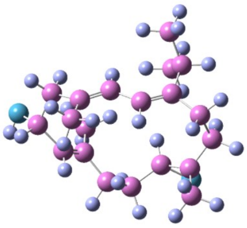

Conf.10
1.809%
-932.02014682

(5S,11*R,12R*)-**4:**

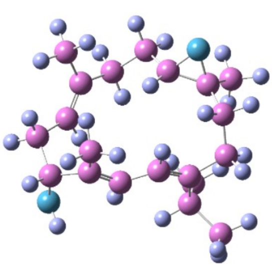

Conf.1
18.344%
-932.02021594

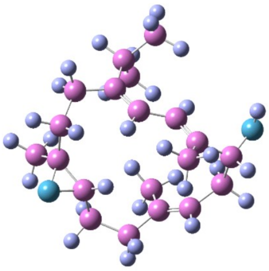

Conf.2
8.240%
-932.01125249

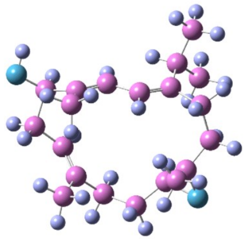

Conf.3
7.269%
-932.01818844

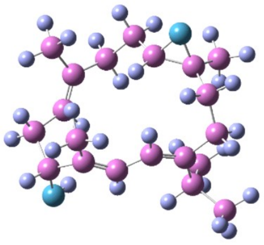

Conf.4
6.894%
-932.01742412

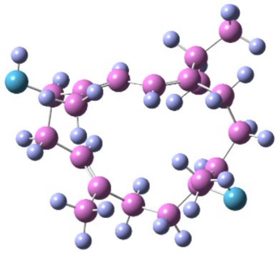

Conf.5
6.381%
-932.01768800

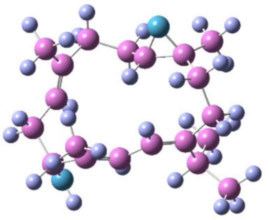

Conf.6
6.102%
-932.01914903

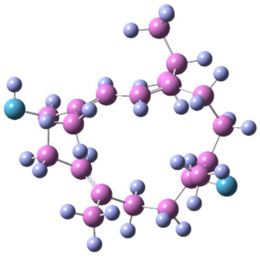

Conf.7
3.780%
-932.01663065

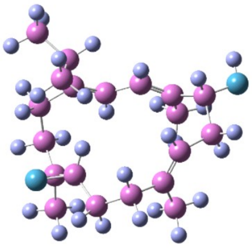

Conf.8
3.253%
-932.01873298

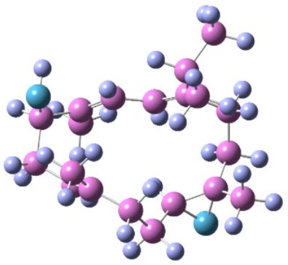

Conf.9
3.038%
-932.01509060

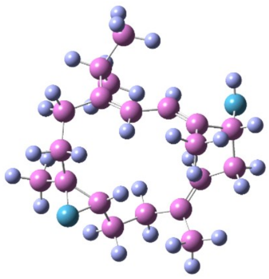

Conf.10
1.887%
-932.01638942

(5S,11*R*,*12R*)-**5:**

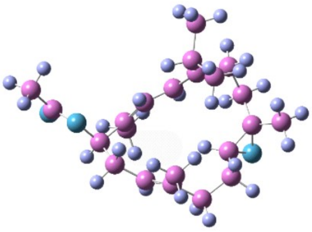

Conf.1
29.706%
-1084.72465736

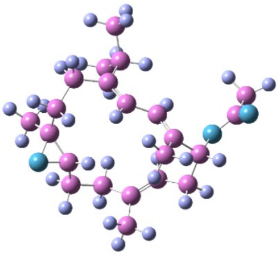

Conf.2
26.304%
-1084.72820327

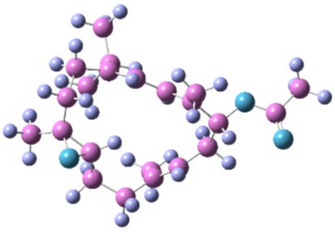

Conf.3
7.695%
-1084.72453048

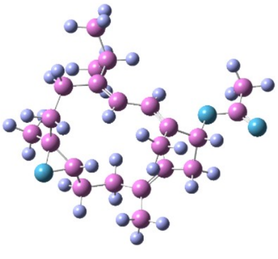

Conf.4
7.200%
-1084.72770455

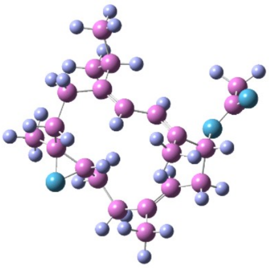

Conf.5
2.739%
-1084.72713683

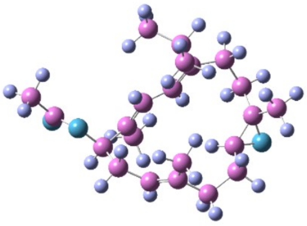

Conf.6
1.926%
-1084.72086174

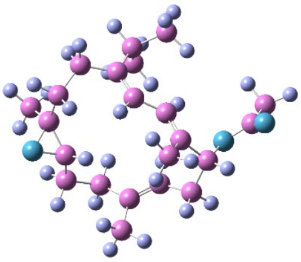

Conf.7
1.655%
-1084.72431105

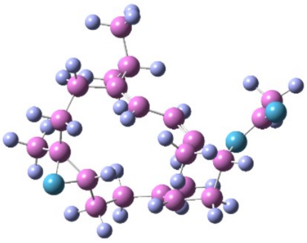

Conf.8
1.584%
-1084.72264945

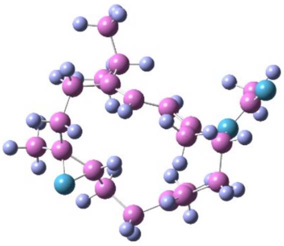

Conf.9
1.537%
-1084.72579464

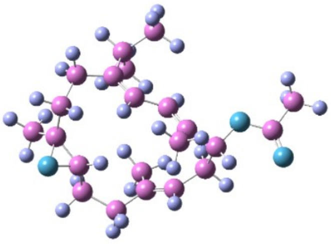

Conf.10
1.065%
-1084.71985734
